# Supplementary material for: Architecture of gene regulatory networks controlling flower development in Arabidopsis thaliana
Source: Nat Commun. 2018 Oct 31;9:4534. doi: 10.1038/s41467-018-06772-3 (PMC6208445; doi:10.1038/s41467-018-06772-3)
Supplement: Supplementary file 1 — Supplementary Information [file 41467_2018_6772_MOESM1_ESM.pdf]

Supplementary Information

for "**Architecture of gene regulatory networks controlling flower development in *Arabidopsis thaliana*"** by **Chen *et al.***

Supplementary Figures

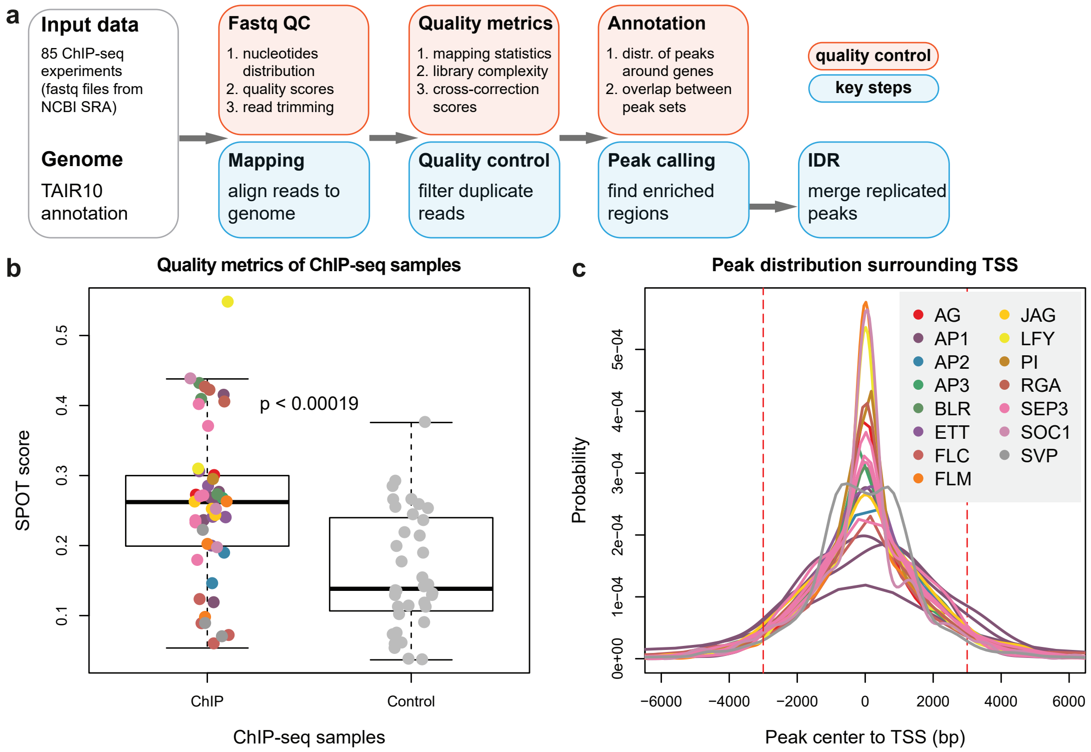

Supplementary Fig. 1: ChIP-seq data analysis pipeline and quality control of ChIP-seq datasets. (**a**) The pipeline used for the ChIP-seq data analysis in this study, adapted from ref.[1]. (**b**) The SPOT score (signal portion of tags) characterizing the enrichment of signal for each library, calculated by the hotspot algorithm[2] by subsampling 10 million reads. For the ChIP experiments, libraries are colored according to the factors used. Color legend as in (**c**). P-value was calcuated based on a Student's t-test (two tailed). (**c**) Distribution of ChIP-seq peaks around the transcription start site (TSS). Dash lines indicate the threshold for target identification.

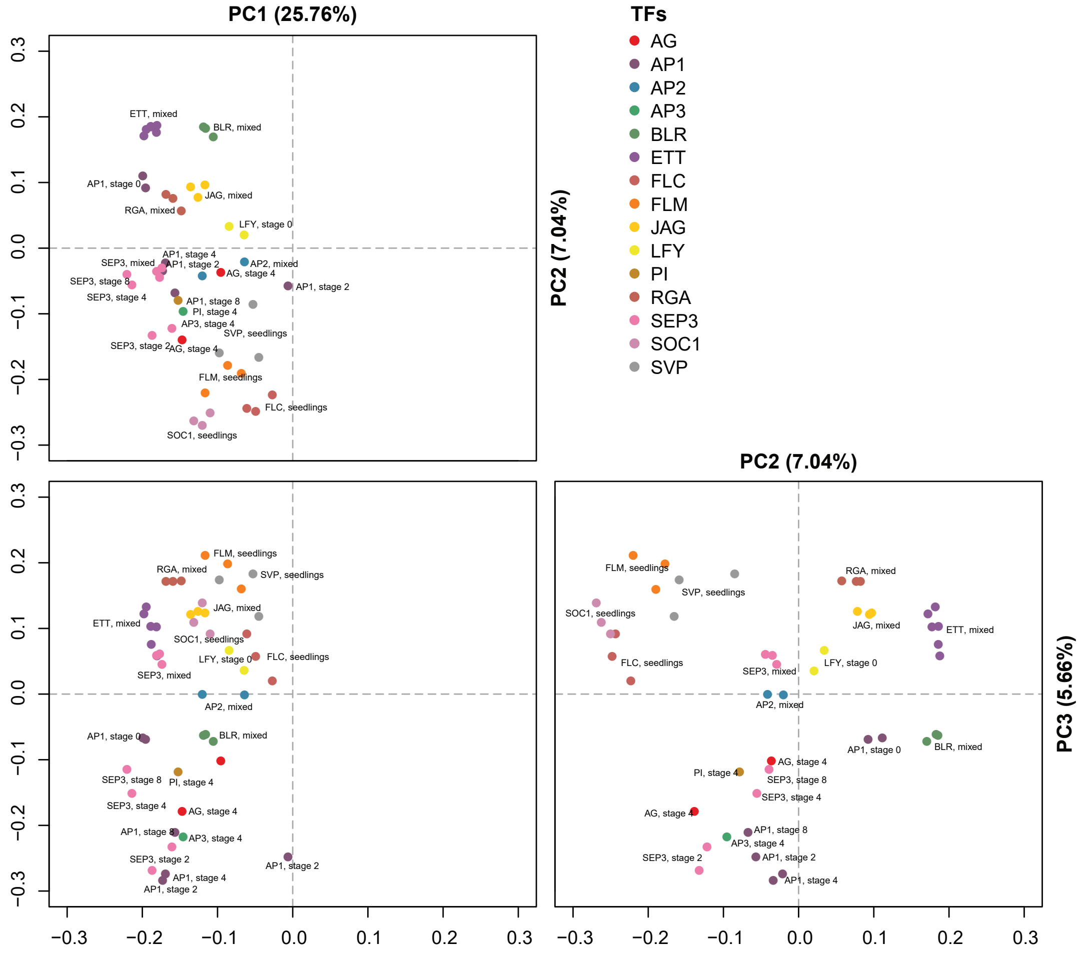

Supplementary Fig. 2: Assessment of public ChIP-seq experiments. Principal component analysis (PCA) of binding profiles of the investigated transcription factors (TFs) in this study. Peak scores generated by MACS2 were used in the analysis. Missing values were assigned to 1 (the lowest peak score). The top three principle components (PCs) were shown. Percentage values in parentheses indicate percentage of variance explained by each PC.

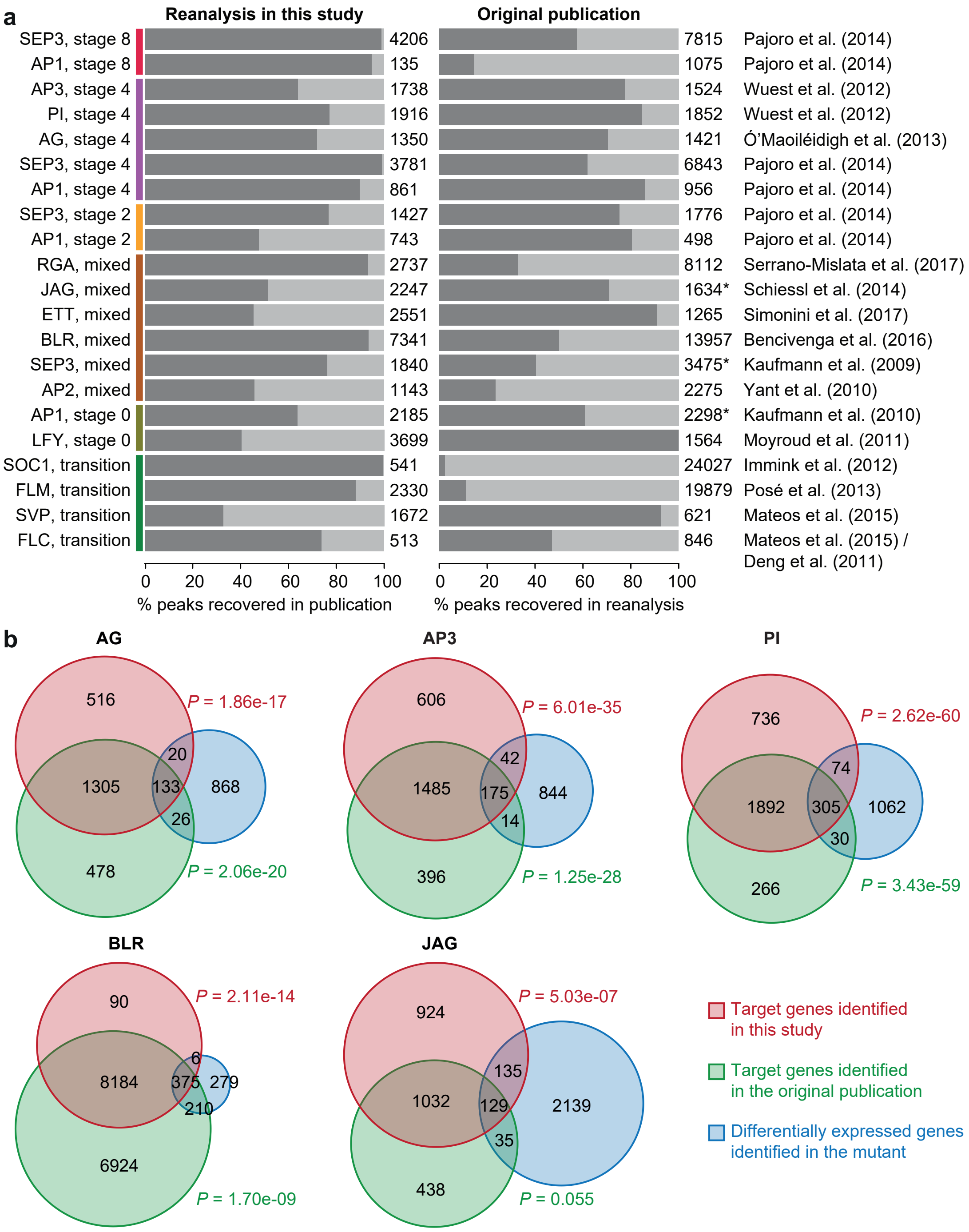
a
Reanalysis in this study
Original publication
SEP3, stage 8 4206 7815 Pajoro et al. (2014)
AP1, stage 8 135 1075 Pajoro et al. (2014)
AP3, stage 4 1738 1524 Wuest et al. (2012)
PI, stage 4 1916 1852 Wuest et al. (2012)
AG, stage 4 1350 1421 Ó'Maoiléidigh et al. (2013)
SEP3, stage 4 3781 6843 Pajoro et al. (2014)
AP1, stage 4 861 956 Pajoro et al. (2014)
SEP3, stage 2 1427 1776 Pajoro et al. (2014)
AP1, stage 2 743 498 Pajoro et al. (2014)
RGA, mixed 2737 8112 Serrano-Mislata et al. (2017)
JAG, mixed 2247 1634* Schiessl et al. (2014)
ETT, mixed 2551 1265 Simonini et al. (2017)
BLR, mixed 7341 13957 Bencivenga et al. (2016)
SEP3, mixed 1840 3475* Kaufmann et al. (2009)
AP2, mixed 1143 2275 Yant et al. (2010)
AP1, stage 0 2185 2298* Kaufmann et al. (2010)
LFY, stage 0 3699 1564 Moyroud et al. (2011)
SOC1, transition 541 24027 Immink et al. (2012)
FLM, transition 2330 19879 Posé et al. (2013)
SVP, transition 1672 621 Mateos et al. (2015)
FLC, transition 513 846 Mateos et al. (2015) / Deng et al. (2011)
0 20 40 60 80 100
% peaks recovered in publication
% peaks recovered in reanalysis
b
AG
516 20 1305 133 868 26 478
P = 1.86e-17
P = 2.06e-20
AP3
606 42 1485 175 844 14 396
P = 6.01e-35
P = 1.25e-28
PI
736 74 1892 305 1062 30 266
P = 2.62e-60
P = 3.43e-59
BLR
90 6 8184 375 279 210 6924
P = 2.11e-14
P = 1.70e-09
JAG
924 135 1032 129 2139 35 438
P = 5.03e-07
P = 0.055
Target genes identified in this study
Target genes identified in the original publication
Differentially expressed genes identified in the mutant

Supplementary Fig. 3 ***(preceding page)*****: Comparison of our re-analysis with published results.** (**a**) Overlap of TF ChIP-seq peaks identified in this study and in the original publications. Left bar charts show the percentage of reanalyzed peaks supported by the corresponding studies, and vice versa on the right. The number of peaks are labeled on the right side of bars. Data sources used in the analysis are listed on the right. Numbers with stars (*) indicate the original peak numbers are not available so the number of target genes were used instead. (**b**) Comparison of the number of target genes identified in this study (red), identified in the original studies (green), and differentially expressed genes identified in the corresponding mutants (blue). P-values are calculated by hypergeometric tests to analyze the significance of overlap between TF-bound genes and differentially expressed genes.

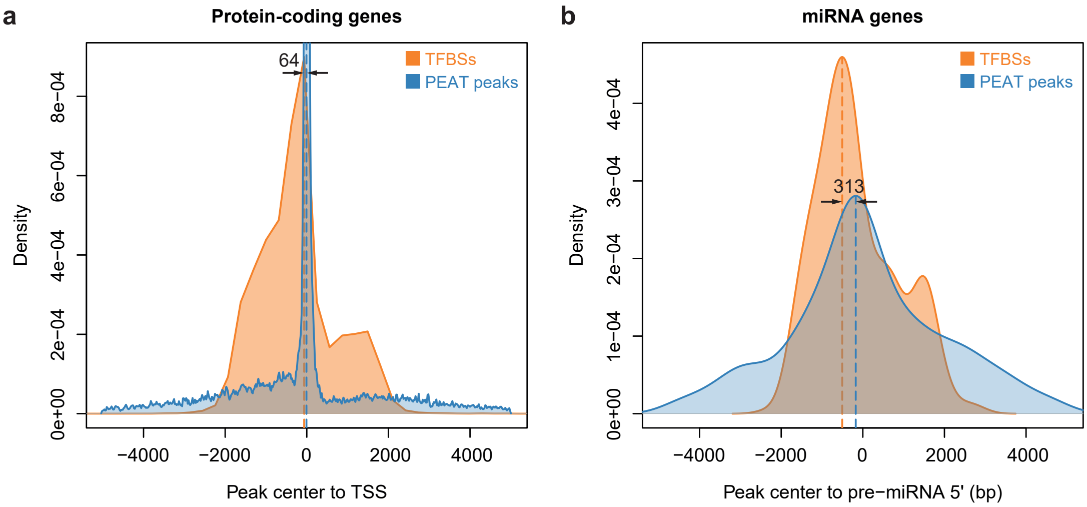

Supplementary Fig. 4: Enrichment of TFBSs around the TSSs of protein-coding or miRNA genes. (**a**) Distribution of TFBSs (orange) and PEAT peaks (blue) around the TSSs of protein-coding genes. (**b**) Distribution of TFBSs and PEAT peaks around the 5' end of pre-miRNAs. PEAT data were taken from ref.[3]. TFBS: transcription factor binding site; TSS: transcription start site; PEAT: paired-end analysis of transcription start sites.

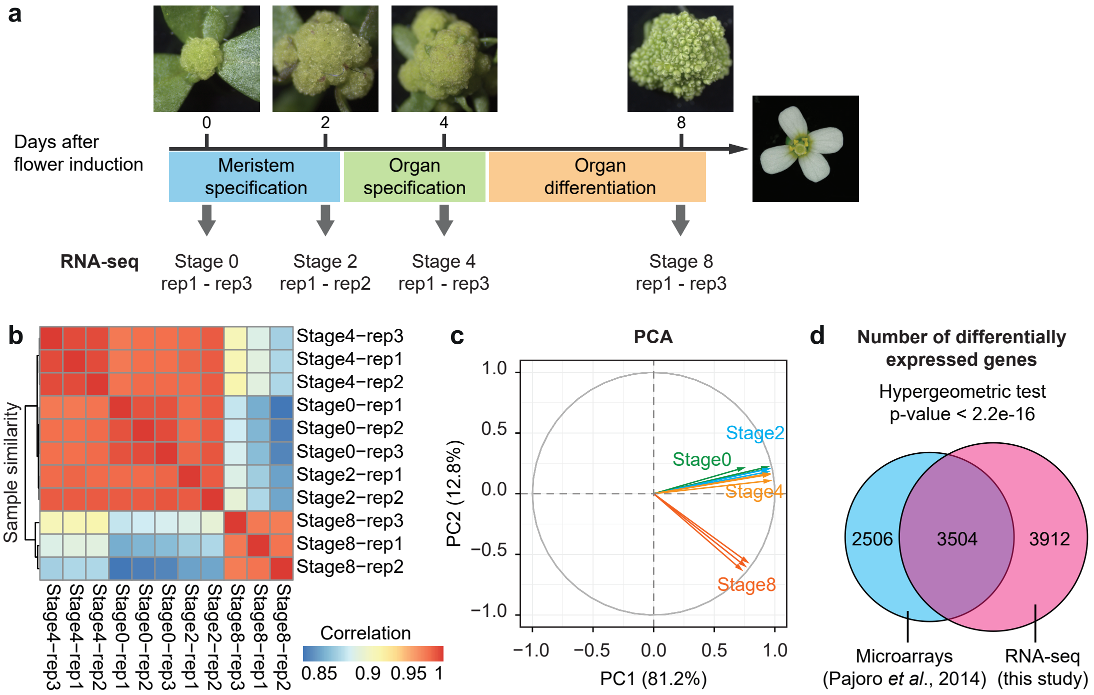

Supplementary Fig. 5: Transcriptome profiling by RNA-seq. (**a**) Strategy for RNA-seq sample preparation. Cartoon is adapted based on ref.[4]. (**b**) Spearman's rank correlation of RNA-seq samples based on their FPKM (fragments per kilobase of exon per million fragments mapped) values. (**c**) Principal component analysis (PCA) of RNA-seq experiments (as labeled in different colors) using all the expressed protein-coding genes, as measured in FPKM (fragments per kilobase of transcript per million mapped reads). Percentage values in parentheses indicate percentage of variance explained by each PC. (**d**) Comparison of the number of differentially expressed genes identified in microarray ref.[4] and RNA-seq data (this study). P-value was calculated by a hypergeometric test.

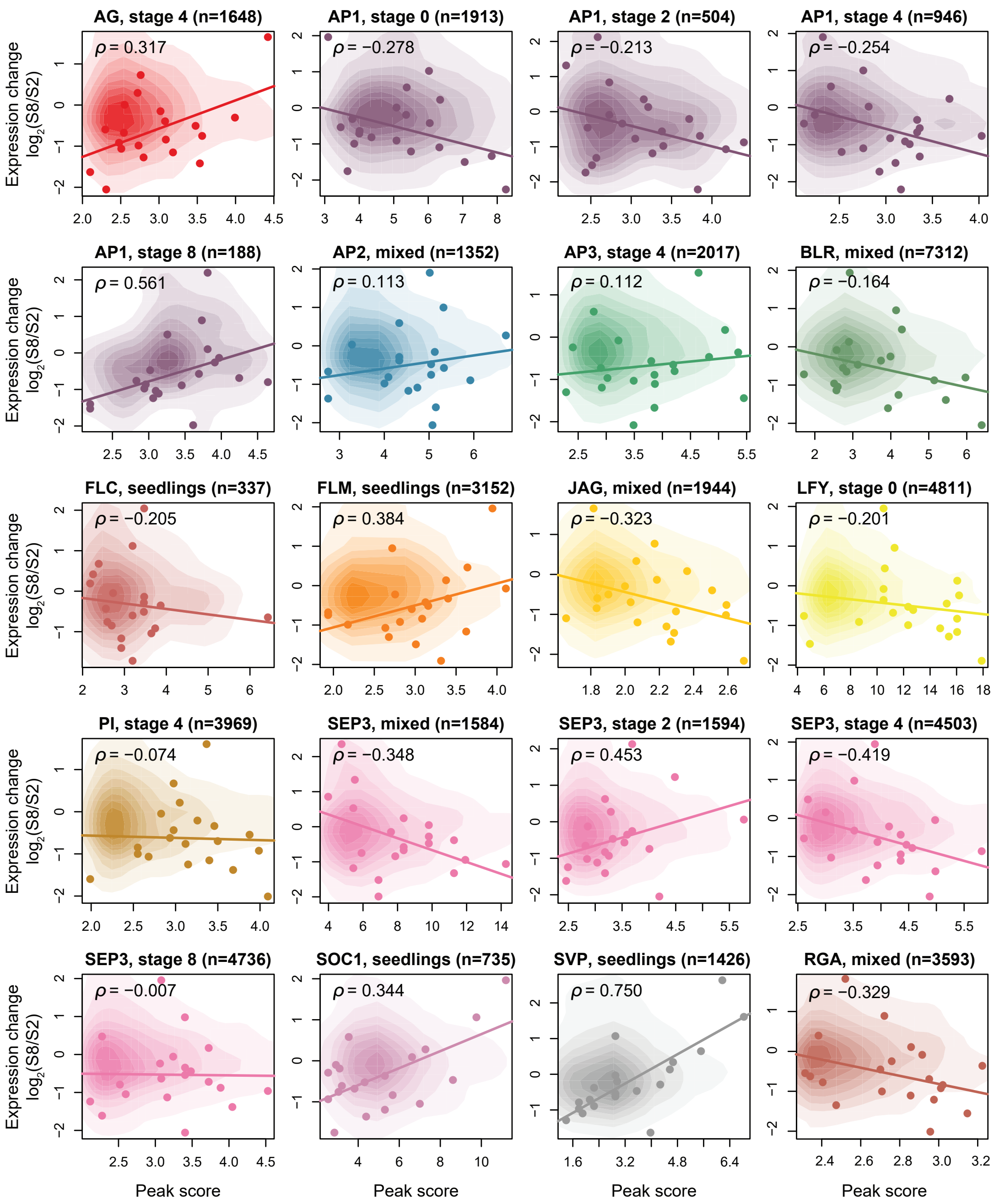
AG, stage 4 (n=1648)
ρ = 0.317
AP1, stage 0 (n=1913)
ρ = −0.278
AP1, stage 2 (n=504)
ρ = −0.213
AP1, stage 4 (n=946)
ρ = −0.254
AP1, stage 8 (n=188)
ρ = 0.561
AP2, mixed (n=1352)
ρ = 0.113
AP3, stage 4 (n=2017)
ρ = 0.112
BLR, mixed (n=7312)
ρ = −0.164
FLC, seedlings (n=337)
ρ = −0.205
FLM, seedlings (n=3152)
ρ = 0.384
JAG, mixed (n=1944)
ρ = −0.323
LFY, stage 0 (n=4811)
ρ = −0.201
PI, stage 4 (n=3969)
ρ = −0.074
SEP3, mixed (n=1584)
ρ = −0.348
SEP3, stage 2 (n=1594)
ρ = 0.453
SEP3, stage 4 (n=4503)
ρ = −0.419
SEP3, stage 8 (n=4736)
ρ = −0.007
SOC1, seedlings (n=735)
ρ = 0.344
SVP, seedlings (n=1426)
ρ = 0.750
RGA, mixed (n=3593)
ρ = −0.329
Expression change log2(S8/S2)
Peak score

Supplementary Fig. 6 ***(preceding page)*****: Correlation of gene expression change and the binding intensity of different transcription factors (TFs).** For each TF, contour plot shows the relationship between the TF binding intensity (in terms of peak score from MACS2) and the expression changes of its target genes. Expression change was measured as the difference of gene expression levels (in terms of FPKM) between stage 8 (S8) and S2. For the scatter plots, genes were binned into percentiles (n=20) based on their expression level, and the median expression and median binding intensity of each bin were plotted. Spearman's rank correlation is shown. The number of genes included in the analysis is indicated in parentheses.

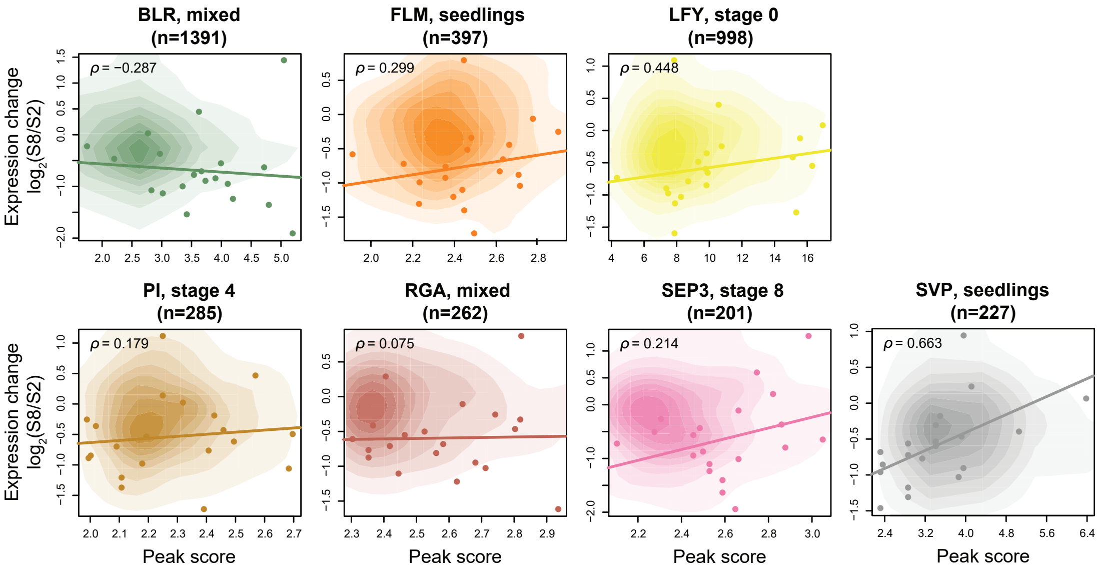

Supplementary Fig. 7 ***(preceding page)*****: Correlation of TF binding intensity and expression change of their specific target genes.** Similar to **Supplementary Fig. 6**, only TFs with more than 200 specific target genes were considered in the analysis. When compared to the observations in **Supplementary Fig. 6**, similar results were found for BLR, FLM, PI, and SVP, but not for LFY, RGA and SEP3. This might indicate that the regulatory effects of LFY, RGA and SEP3 are more determined by their interacting partners.

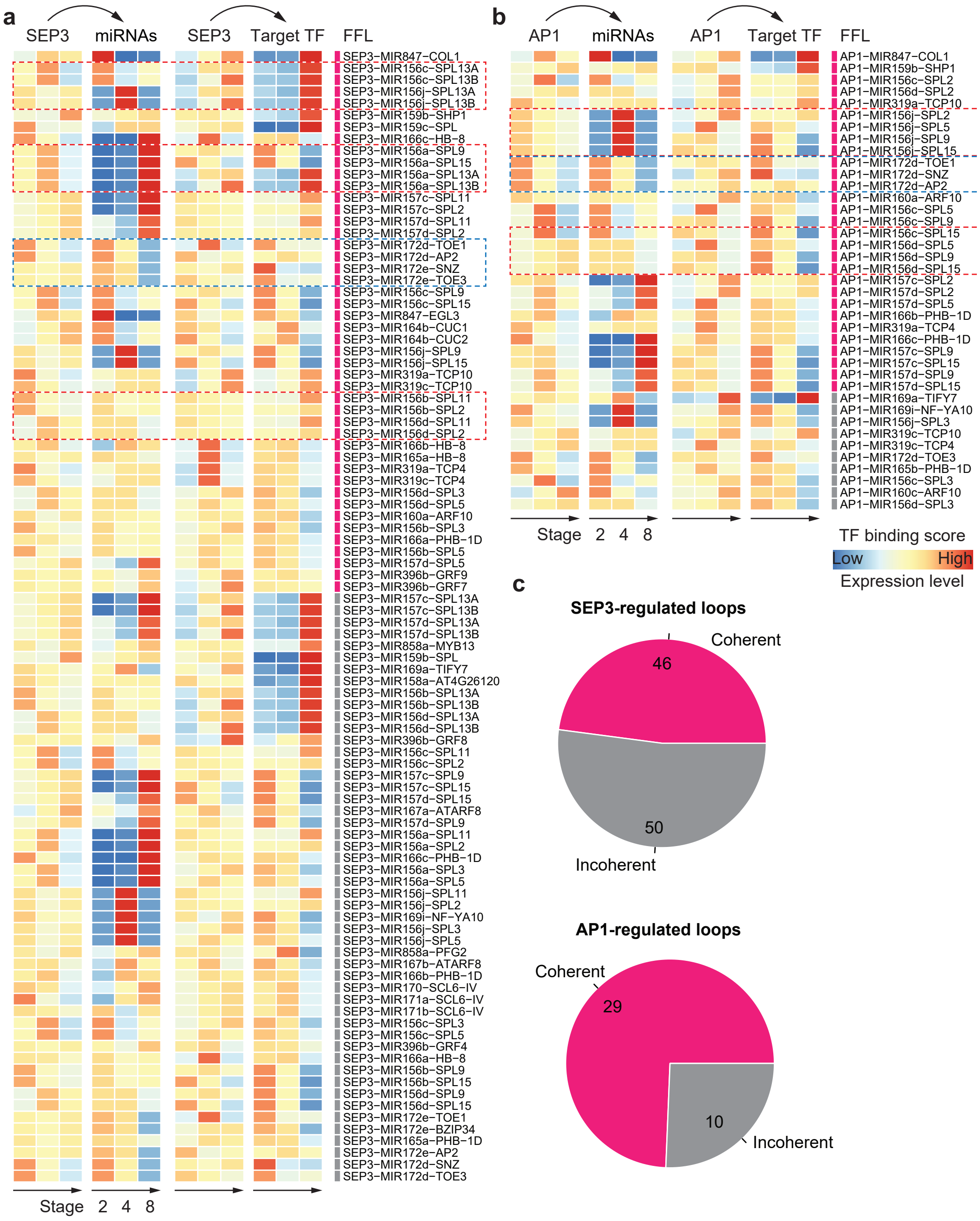
a
SEP3
miRNAs
SEP3
Target TF
FFL
SEP3−MIR847−COL1
SEP3−MIR156c−SPL13A
SEP3−MIR156c−SPL13B
SEP3−MIR156j−SPL13A
SEP3−MIR156j−SPL13B
SEP3−MIR159b−SHP1
SEP3−MIR159c−SPL
SEP3−MIR166c−HB−8
SEP3−MIR156a−SPL9
SEP3−MIR156a−SPL15
SEP3−MIR156a−SPL13A
SEP3−MIR156a−SPL13B
SEP3−MIR157c−SPL11
SEP3−MIR157c−SPL2
SEP3−MIR157d−SPL11
SEP3−MIR157d−SPL2
SEP3−MIR172d−TOE1
SEP3−MIR172d−AP2
SEP3−MIR172e−SNZ
SEP3−MIR172e−TOE3
SEP3−MIR156c−SPL9
SEP3−MIR156c−SPL15
SEP3−MIR847−EGL3
SEP3−MIR164b−CUC1
SEP3−MIR164b−CUC2
SEP3−MIR156j−SPL9
SEP3−MIR156j−SPL15
SEP3−MIR319a−TCP10
SEP3−MIR319c−TCP10
SEP3−MIR156b−SPL11
SEP3−MIR156b−SPL2
SEP3−MIR156d−SPL11
SEP3−MIR156d−SPL2
SEP3−MIR166b−HB−8
SEP3−MIR165a−HB−8
SEP3−MIR319a−TCP4
SEP3−MIR319c−TCP4
SEP3−MIR156d−SPL3
SEP3−MIR156d−SPL5
SEP3−MIR160a−ARF10
SEP3−MIR156b−SPL3
SEP3−MIR166a−PHB−1D
SEP3−MIR156b−SPL5
SEP3−MIR157d−SPL5
SEP3−MIR396b−GRF9
SEP3−MIR396b−GRF7
SEP3−MIR157c−SPL13A
SEP3−MIR157c−SPL13B
SEP3−MIR157d−SPL13A
SEP3−MIR157d−SPL13B
SEP3−MIR858a−MYB13
SEP3−MIR159b−SPL
SEP3−MIR169a−TIFY7
SEP3−MIR158a−AT4G26120
SEP3−MIR156b−SPL13A
SEP3−MIR156b−SPL13B
SEP3−MIR156d−SPL13A
SEP3−MIR156d−SPL13B
SEP3−MIR396b−GRF8
SEP3−MIR156c−SPL11
SEP3−MIR156c−SPL2
SEP3−MIR157c−SPL9
SEP3−MIR157c−SPL15
SEP3−MIR157d−SPL15
SEP3−MIR167a−ATARF8
SEP3−MIR157d−SPL9
SEP3−MIR156a−SPL11
SEP3−MIR156a−SPL2
SEP3−MIR166c−PHB−1D
SEP3−MIR156a−SPL3
SEP3−MIR156a−SPL5
SEP3−MIR156j−SPL11
SEP3−MIR156j−SPL2
SEP3−MIR169i−NF−YA10
SEP3−MIR156j−SPL3
SEP3−MIR156j−SPL5
SEP3−MIR858a−PFG2
SEP3−MIR167b−ATARF8
SEP3−MIR166b−PHB−1D
SEP3−MIR170−SCL6−IV
SEP3−MIR171a−SCL6−IV
SEP3−MIR171b−SCL6−IV
SEP3−MIR156c−SPL3
SEP3−MIR156c−SPL5
SEP3−MIR396b−GRF4
SEP3−MIR166a−HB−8
SEP3−MIR156b−SPL9
SEP3−MIR156b−SPL15
SEP3−MIR156d−SPL9
SEP3−MIR156d−SPL15
SEP3−MIR172e−TOE1
SEP3−MIR172e−BZIP34
SEP3−MIR165a−PHB−1D
SEP3−MIR172e−AP2
SEP3−MIR172d−SNZ
SEP3−MIR172d−TOE3
Stage 2 4 8
b
AP1
miRNAs
AP1
Target TF
FFL
AP1−MIR847−COL1
AP1−MIR159b−SHP1
AP1−MIR156c−SPL2
AP1−MIR156d−SPL2
AP1−MIR319a−TCP10
AP1−MIR156j−SPL2
AP1−MIR156j−SPL5
AP1−MIR156j−SPL9
AP1−MIR156j−SPL15
AP1−MIR172d−TOE1
AP1−MIR172d−SNZ
AP1−MIR172d−AP2
AP1−MIR160a−ARF10
AP1−MIR156c−SPL5
AP1−MIR156c−SPL9
AP1−MIR156c−SPL15
AP1−MIR156d−SPL5
AP1−MIR156d−SPL9
AP1−MIR156d−SPL15
AP1−MIR157c−SPL2
AP1−MIR157d−SPL2
AP1−MIR157d−SPL5
AP1−MIR166b−PHB−1D
AP1−MIR319a−TCP4
AP1−MIR166c−PHB−1D
AP1−MIR157c−SPL9
AP1−MIR157c−SPL15
AP1−MIR157d−SPL9
AP1−MIR157d−SPL15
AP1−MIR169a−TIFY7
AP1−MIR169i−NF−YA10
AP1−MIR156j−SPL3
AP1−MIR319c−TCP10
AP1−MIR319c−TCP4
AP1−MIR172d−TOE3
AP1−MIR165b−PHB−1D
AP1−MIR156c−SPL3
AP1−MIR160c−ARF10
AP1−MIR156d−SPL3
Stage 2 4 8
TF binding score
Low
High
Expression level
c
SEP3-regulated loops
Coherent
46
50
Incoherent
AP1-regulated loops
Coherent
29
10
Incoherent

Supplementary Fig. 8 ***(preceding page)*****: Predicted AP1- and SEP3-regulated feed-forward loops (FFLs).** Heatmaps showing the regulatory relationships between the master regulators ((**a**) for SEP3, and (**b**) for AP1) and their miRNA targets or TF targets. We used time-series genomic binding (by ChIP-seq) and gene expression data (by mRNA-seq or miRNA-seq) to derive the relationships between the master regulator and its targets, as positive or negative regulation. Given that miRNAs generally repress their target genes, 'regulator-miRNA-TF' feed-forward loop is considered as coherent (in pink color) when the 'regulator' regulates 'miRNA' and 'TF' targets with opposing effect. Otherwise, it is considered as an incoherent loop (grey). Examples of AP1/SEP3-miR156-SPL (in red boxes) and AP1/SEP3-miR172-AP2 FFLs are discussed in the main text. (**c**) The number of coherent and incoherent FFLs controlled by SEP3 and AP1. A full list of these FFLs can be found in **Supplementary Data 6**. Experimental validation of candidate genes can be found in **Supplementary Fig. 9**.

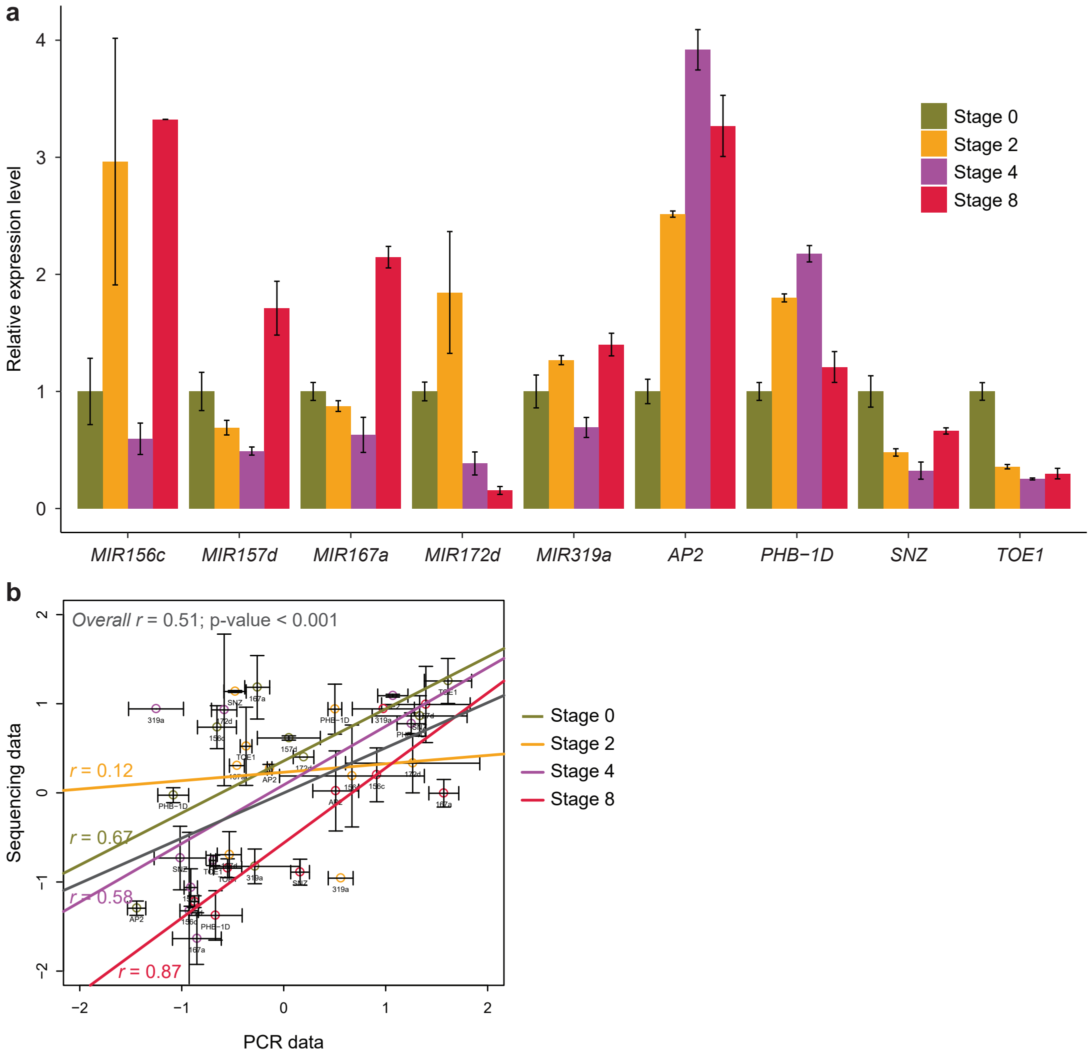

Supplementary Fig. 9: Validation of miRNA-seq and RNA-seq data. (**a**) Validation of expression levels during flower development for selected genes, either by stem-loop RT-qPCR (for miRNA genes) or by qRT-PCR (for TFs). Tissues representing four different developmental stages flowers were harvested from pAP1::AP1-GR *ap1 cal* plants according days of DEX induction (DAI) for expression analysis. U6 and Tip41 were taken as internal control to normalize the expression level of miRNA genes and other TF genes, respectively. Result shown here represents data from three independent biological replicates (n=3). Candidate genes were selected from the **Supplementary Fig. 8**. (**b**) Correlation of gene expression levels by experimental validation and sequencing results. For visualization purpose, expression data for each gene were centered to the mean value and scaled as s.t.d.=1. Pearson correlation coefficients (r) between experimental data and sequencing data for the selected genes were shown for each stage. Bar=mean ±s.d..

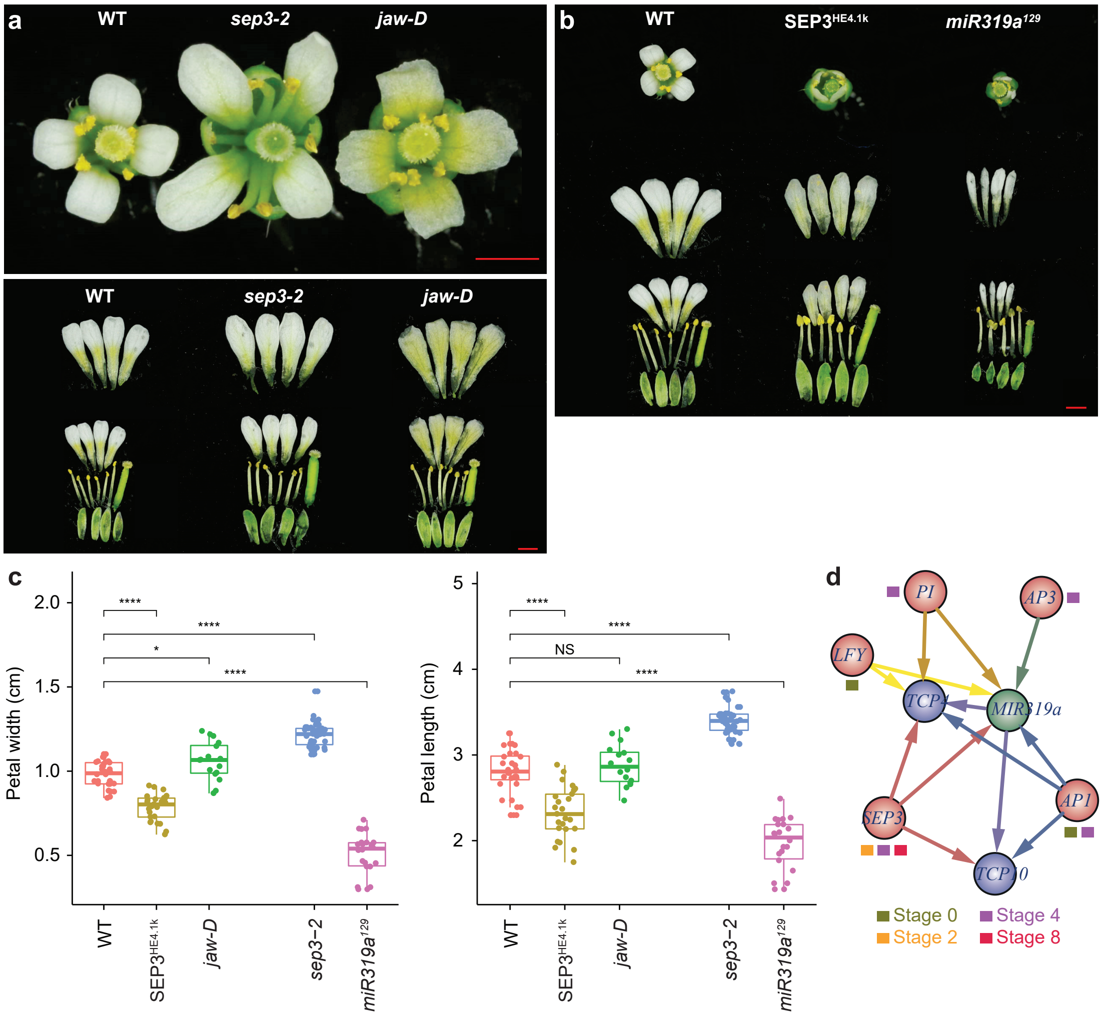

Supplementary Fig. 10: *SEP3* and *MIR319a* have opposite role in petal deveopment. (**a**) Pictures of single flower and separated flower organs of wild type (WT), *sep3-2* mutant and *MIR319a* overexpression line (*jaw-D*). (**b**) Performance of single flower and each whorl of flower organs of WT, SEP3 enhanced expression line ($SEP3^{HE4.1k}$) and *MIR319a* mutant line ($miR319a^{129}$). (**c**) Boxplots showing the petal width (left) and petal length (right) of WT, *sep3-2*, *jaw-D*, $SEP3^{HE4.1k}$ and $miR319a^{129}$ plants. Flowers from five to eight plants with the same genotype were used for data collection. Five flowers were harvested from the main inflorescences of one single plant. Data represents the mean ± s.e.. Significance codes, ****: p-value $<$ 0.0001, *: p-value $<$ 0.05, and NS: not significant, by Mann-Whitney tests. (**d**) The sub network centered by *MIR319a*. The regulation relationships detected by ChIP-seq data in different stages are indicated. Scale bar=1 mm.

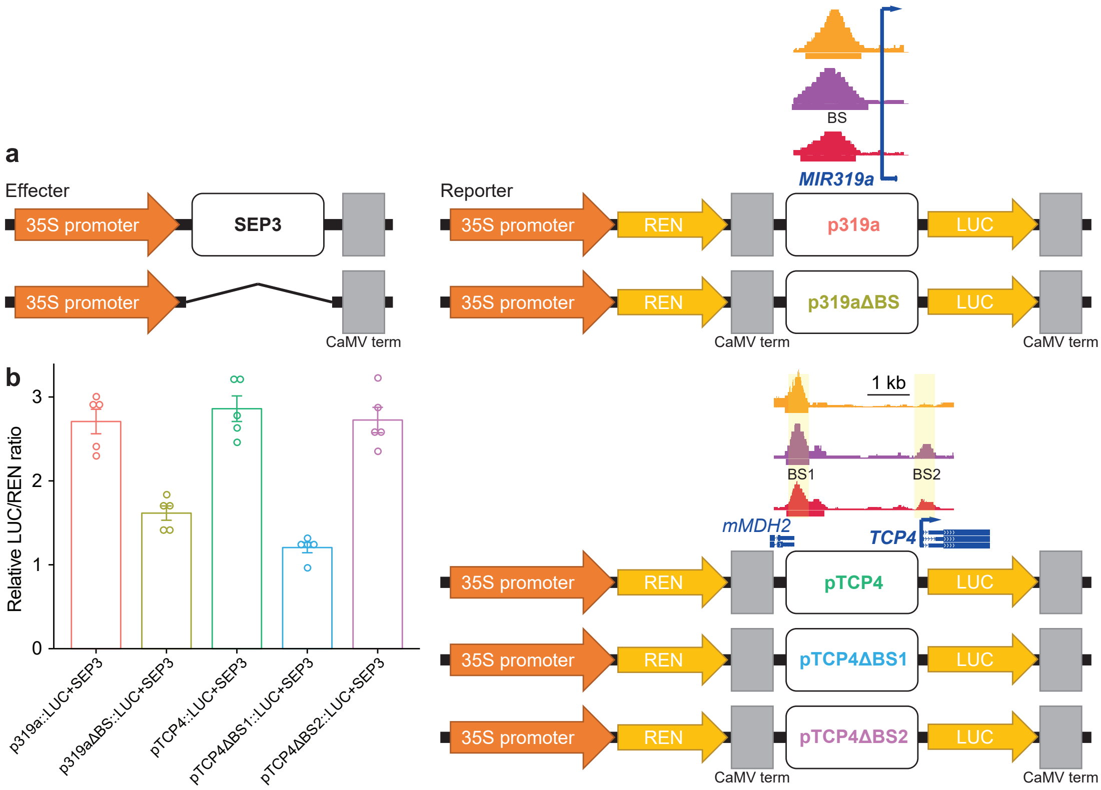

Supplementary Fig. 11: Effects of SEP3 direct binding to *TCP4* and *MIR319a* promoters. (**a**) Diagrams showing the reporter and effector constructs of dual-luciferase reporter based transient assay. CaMV35S promoter driven SEP3 is the effector and different versions of *TCP4* or *MIR319a* promoters (with or without a SEP3 site peak (BS)) driven firefly luciferase (LUC) are used as reporters. CaMV35S promoter driven Renilla luciferase(REN) was simultaneously expressed with test promoters in the same reporter construct to serve as internal control. (**b**) Relative LUC to REN ratio of co-transfection of SEP3 and different reporters. The ratio of LUC/REN of the empty vector plus corresponding promoter (CaMV35S promoter alone as effecter; CaMV35S promoter driven REN together with test promoter driven LUC as reporter) was considered for callibration (set as 1). Data represents the mean $\pm$ s.e. of five independent replicates. Significance codes, *: p-value $<$ 0.05 and NS: not significant, by two-tailed Student's t-tests.

Supplementary Tables

TF (gene ID)	Stage[1]	#peaks/#targets[2]	Data source[3]	Reference
AG (AT4G18960)	Stage 4	1350 / 2006	SRP020911	Ó'Maoiléidigh et al.[5]
AP1 (AT1G69120)	Stage 0	1682 / 2185	SRP002174	Kaufmann, Wellmer *et al.*[6]
	Stage 2	743 / 622	SRP022770	Pajoro *et al.*[4]
	Stage 4	861 / 1091		
	Stage 8	135 / 224		
AP2 (AT4G36920)	Mixed	1143 / 1642	SRP002328	Yant *et al.*[7]
AP3 (AT3G54340)	Stage 4	1738 / 2336	SRP013458	Wuest *et al.*[8]
BLR (AT5G02030)	Mixed	7341 / 8747	SRP070904	Bencivenga *et al.*[9]
ETT (AT2G33860)	Mixed	2551 / 3917	ERP021928	Simonini *et al.*[10]
FLC (AT5G10140)	Transition	513 / 604	SRP005412; SRP037581	Deng *et al.*[11]; Mateos *et al.*[12]
FLM (AT1G77080)	Transition	2330 / 3708	SRP026163	Posé *et al.*[13]
JAG (AT1G68480)	Mixed	1580 / 2247	SRP031786	Schiessl *et al.*[14]
LFY (AT5G61850)	Stage 0	3699 / 5776	SRP003928	Moyroud *et al.*[15]
PI (AT5G20240)	Stage 4	1916 / 3039	SRP013458	Wuest *et al.*[8]
RGA (AT2G01570)	Mixed	2737 / 4213	SRP099840	Serrano-Mislata *et al.*[16]
SEP3 (AT1G24260)	Mixed	1712 / 1840	SRP000783	Kaufmann *et al.*[17]
	Stage 2	1427 / 1878	SRP022770	Pajoro *et al.*[4]
	Stage 4	3781 / 5271		
	Stage 8	4206 / 5564		
SOC1 (AT2G45660)	Transition	541 / 896	SRP020612	Immink *et al.*[18]
SVP (AT2G22540)	Transition	1672 / 2830	SRP037581	Mateos *et al.*[12]

Supplementary Table 1: TF ChIP-seq datasets used in this study.

[1] Developmental stages as denoted in **Fig. 1a**;

[2] #: number of;

[3] Raw data were downloaded from the NCBI SRA (`http://www.ncbi.nlm.nih.gov/sra/`) database.

Supplementary Notes

Supplementary Note 1

mRNA-seq experiments and analysis For each single experiment, 0.1 gram tissue was harvested in liquid nitrogen. Total RNA was extracted using RNeasy Plant Mini Kit (Qiagen, Germany) according to manufacturer and 2 μg total RNA was used to produce libraries using TruSeq Stranded Total RNA LT Sample Prep Kit (Illumina, USA). Pooled libraries were submitted to a NextSeq-550 sequencing platform (Illumina, USA) for sequencing. Two or three biological replicates were generated for each sample (**Supplementary Fig. 5a**). FASTQ files were demultiplexed from BCL files using the 'bcl2fastq' command.

mRNA-seq reads were mapped to A. thaliana reference genome (TAIR10) using STAR (version 020201)[19]. Expression levels (in terms of FPKM, fragments per kilobase of transcript per million mapped reads) of all annotated protein-coding genes were estimated by RSEM (version 1.2.22)[20]. FPKM (fragments per kilobase of transcript per million mapped reads) values as defined by RSEM were added a pesudo-value of 1e-6 (to avoid zeros) and then log2-transformed. A gene was considered as expressed only its estimated FPKM > 0.1 at least one sample. Reproducibility of RNA-seq experiments was evaluated by a Spearman's correlation analysis (**Supplementary Fig. 5b**) and a principal component analysis (PCA; **Supplementary Fig. 5c**). Differentially expressed genes across the four developmental stages were identified by analysis of variance (ANOVA) based on FPKM values. Resulting p-values were adjusted for multiple comparisons by false discovery rate (FDR)[21]. Genes were considered as differentially expressed if they showed at least two-fold changes with FDR < 0.05 (**Supplementary Data 4**). The differentially expressed genes were further compared with those detected by the microarray experiment (**Supplementary Fig. 5d**).

miRNA-seq experiments and analysis Totally RNA was extracted from 0.1g inflorescences at different developmental stages, respectively with the miRNeasy Mini kit (Qiagen) for later miRNA purification. Small RNA libraries were constructed using the TruSeq Small RNA kit (Illumina) per the manufacturer's instructions. Three biological replicates each sample were used for sequencing.

miRNA-seq reads were firstly subjected to quality and adapter trimming using the Trim

Galore tool (`https://github.com/FelixKrueger/TrimGalore`) with parameters "`-length 17 -phred33`". Clean reads were mapped to A. thaliana genome using the STAR algorithm with the following parameters: "`-outSAMunmapped Within -outSAMtype BAM SortedByCoordinate-quantMode GeneCounts -outFilterMultimapNmax 10 -outFilterMismatchNoverLmax 0.03 -outFilterScoreMinOverLread 0 -outFilterMatchNminOverLread 0 -outFilterMatchNmin 16 -alignSJDBoverhangMin 1000 -alignIntronMax 1 -genomeLoad NoSharedMemory`". miRNAs were quantified in RPM (reads per million mapped reads) values based on the count number from STAR output.

Prediction of miRNA target genes Target genes of miRNAs were predicted by the TargetFinder tool[22], with a prediction score cut-off value set to 4.

Supplementary Note 2

ChIP-seq datasets and analysis We collected 106 ChIP-seq datasets for 15 flowering-related TFs from 16 studies (**Supplementary Data 1**). Based on initial assessment, the binding enrichment for SVP in two older ChIP-seq experiments[23] and for FLM-β and FLM-δ[13] was relatively low, and thus the corresponding datasets were removed from further analysis. Besides, ChIP-seq experiments that were performed in specific mutants[12,17] were also removed. In this manner, the final ChIP-seq datasets used in the study include 85 datasets (49 for ChIP and 36 for input control) for 15 floral TFs (**Supplementary Data 1**) can be assigned to five representative developmental stages of mixed tissues (**Supplementary Table 1**; **Fig. 1a**). The raw FASTQ files for these ChIP-seq experiments were downloaded from the NCBI Sequence Read Archive (SRA; accession IDs are listed in **Supplementary Data 1**).

We followed the ChIP-seq data analysis guidelines[24,25] recommended by the ENCODE project and have developed an analysis pipeline[1] consisting of quality control, read mapping, peak calling, assessment of reproducibility among biological replicates, and peak annotation to preprocess all raw data in a standardized and uniform manner. Specifically, the quality of the raw data (FASTQ files) was evaluated by FastQC (`http://www.bioinformatics.babraham.ac.uk/projects/fastqc/`). Reads were then mapped to the A. thaliana genome (TAIR10) using Bowtie (version 1.1.2)[26] with parameters "`-threads 8 -n 2 -m 3 -k 1 -best -chunkmbs 256 -q`". Redundant reads were removed using Picard tools (v2.60; `http://broadinstitute.`

github.io/picard/). Peak calling was performed using MACS2 (version 2.1.0)[27]. Duplicated reads were not considered (-keep-dup=1) during peak calling in order to achieve a better specificity[25]. The "-mfold" parameter was set as "2-20" to build the model. The parameter '-call-summits' was used in peak calling. A relaxed threshold of p-value (p-value $<$ 1e-2) was suggested in order to enable the correct computation of IDR (irreproducible discovery rate) values[24]. Following the recommendations for the analysis of self-consistency and reproducibility between replicates (https://sites.google.com/site/anshulkundaje/projects/idr)[28], control samples were combined into one single control among the replicated experiments. Peaks across replicates with an IDR $<$ 0.05 were retained. For visualization purpose, wiggle tracks (using pooled data across biological replicates) were generated by DeepTools[29] with the command "bamCoverage"; read coverage was normalized as RPKM (Reads Per Kilobase per Million reads). ChIP-seq tracks were visualized in the WashU Epigenome Browser[30].

To define target genes, annotated protein-coding genes were obtained from TAIR10 (http://www.arabidopsis.org/) and miRNA genes from miRBase Release 21 (http://www.mirbase.org/)[31]. For each TF, target genes were assigned when their transcription start sites (TSSs) are positioned within 3 kb from the peak regions. If multiple peaks can be assigned to a same gene, only the closest peak was considered as its putative binding site. For miRNA genes, the 5'end of their precursors (pre-miRNAs) were considered as the start.

To perform a quantitative analysis of SEP3 binding and target gene expression across flower developmental stages (**Fig. 4b**), SEP3 binding intensity was quantified as normalized peak scores as described in ref.[32].

Annotation of transcription factors To annotate Arabidopsis TFs a comprehensive and unbiased manner, an extensive list of TFs was compiled by combining all TFs predicted in the databases of PlantTFDB[33], PlnTFDB[34], DBD[35] and ref.[36]. In total 2348 TFs were included in the analysis.

Supplementary References

1. Chen, D. & Kaufmann, K. in *Methods in molecular biology (Clifton, NJ)* 239–269 (Humana Press, New York, NY, 2017). ISBN: 978-1-4939-7124-4. doi:10.1007/978-1-4939-7125-1_16. <http://link.springer.com/10.1007/978-1-4939-7125-1{_}16>.

2. John, S. *et al. Chromatin accessibility pre-determines glucocorticoid receptor binding patterns* 2011. doi:10.1038/ng.759.

3. Morton, T. *et al.* Paired-end analysis of transcription start sites in Arabidopsis reveals plant-specific promoter signatures. *The Plant cell* **26,** 2746–60. ISSN: 1532-298X (2014).

4. Pajoro, A. *et al.* Dynamics of chromatin accessibility and gene regulation by MADS-domain transcription factors in flower development. *Genome Biology* **15,** R41. ISSN: 1474760X (2014).

5. ÓMaoiléidigh, D. S. *et al.* Control of Reproductive Floral Organ Identity Specification in Arabidopsis by the C Function Regulator AGAMOUS. *Plant Cell* **25,** 2482–2503. ISSN: 1040-4651 (2013).

6. Kaufmann, K., Pajoro, A. & Angenent, G. C. *Regulation of transcription in plants: Mechanisms controlling developmental switches* 2010. doi:10.1038/nrg2885. arXiv:arXiv:1507.02142v2.

7. Yant, L. *et al.* Orchestration of the Floral Transition and Floral Development in Arabidopsis by the Bifunctional Transcription Factor APETALA2. *Plant Cell* **22,** 2156–2170. ISSN: 1040-4651 (2010).

8. Wuest, S. E. *et al.* Molecular basis for the specification of floral organs by APETALA3 and PISTILLATA. *Proceedings of the National Academy of Sciences* **109,** 13452–13457. ISSN: 0027-8424 (2012).

9. Bencivenga, S., Serrano-Mislata, A., Bush, M., Fox, S. & Sablowski, R. Control of Oriented Tissue Growth through Repression of Organ Boundary Genes Promotes Stem Morphogenesis. *Developmental Cell* **39,** 198–208. ISSN: 18781551 (2016).

10. Simonini, S., Bencivenga, S., Trick, M. & Østergaard, L. Auxin-Induced Modulation of ETTIN Activity Orchestrates Gene Expression in Arabidopsis. *The Plant cell* **29,** 1864–1882. ISSN: 1532-298X (2017).

11. Deng, W. *et al.* FLOWERING LOCUS C (FLC) regulates development pathways throughout the life cycle of Arabidopsis. *Proceedings of the National Academy of Sciences* **108,** 6680–6685. ISSN: 0027-8424 (2011).

12. Mateos, J. L. *et al.* Combinatorial activities of SHORT VEGETATIVE PHASE and FLOWERING LOCUS C define distinct modes of flowering regulation in Arabidopsis. *Genome Biology* **16,** 31. ISSN: 1474760X (2015).

13. Posé, D. *et al.* Temperature-dependent regulation of flowering by antagonistic FLM variants. *Nature* **503,** 414–417. ISSN: 00280836 (2013).

14. Schiessl, K., Muino, J. M. & Sablowski, R. Arabidopsis JAGGED links floral organ patterning to tissue growth by repressing Kip-related cell cycle inhibitors. *Proceedings of the National Academy of Sciences* **111,** 2830–2835. ISSN: 0027-8424 (2014).

15. Moyroud, E. *et al.* Prediction of regulatory interactions from genome sequences using a biophysical model for the Arabidopsis LEAFY transcription factor. *The Plant cell* **23,** 1293–306. ISSN: 1532-298X (2011).

16. Serrano-Mislata, A. *et al.* DELLA genes restrict inflorescence meristem function independently of plant height. *Nature Plants* **3,** 749–754. ISSN: 20550278 (2017).

17. Kaufmann, K. *et al.* Target genes of the MADS transcription factor sepallata3: Integration of developmental and hormonal pathways in the arabidopsis flower. *PLoS Biology* **7,** 0854–0875. ISSN: 15449173 (2009).

18. Immink, R. G. H. *et al.* Characterization of SOC1's Central Role in Flowering by the Identification of Its Upstream and Downstream Regulators. *Plant Physiology* **160,** 433–449. ISSN: 0032-0889 (2012).

19. Dobin, A. *et al.* STAR: Ultrafast universal RNA-seq aligner. *Bioinformatics* **29,** 15–21. ISSN: 13674803 (2013).

20. Li, B. & Dewey, C. N. RSEM: Accurate transcript quantification from RNA-Seq data with or without a reference genome. *BMC Bioinformatics* **12,** 323. ISSN: 1471-2105 (2011).

21. Benjamini, Y. & Hochberg, Y. Controlling the false discovery rate: a practical and powerful approach to multiple testing. *Journal of the Royal Statistical Society B* **57,** 289–300. ISSN: 00359246 (1995).

22. Fahlgren, N. & Carrington, J. C. miRNA Target Prediction in Plants. *Methods in Molecular Biology (Clifton, N.J.)* **592,** 51–57. ISSN: 1940-6029 (2010).

23. Gregis, V. *et al.* Identification of pathways directly regulated by SHORT VEGETATIVE PHASE during vegetative and reproductive development in Arabidopsis. *Genome Biology* **14,** R56. ISSN: 1474-760X (2013).

24. Landt, S. G. *et al. ChIP-seq guidelines and practices of the ENCODE and modENCODE consortia* 2012. doi:10.1101/gr.136184.111.

25. Bailey, T. *et al.* Practical Guidelines for the Comprehensive Analysis of ChIP-seq Data. *PLoS Computational Biology* **9.** ISSN: 1553734X. doi:10.1371/journal.pcbi.1003326 (2013).

26. Langmead, B., Trapnell, C., Pop, M. & Salzberg, S. L. Ultrafast and memory-efficient alignment of short DNA sequences to the human genome. *Genome Biology* **10,** R25. ISSN: 1465-6906 (2009).

27. Zhang, Y. *et al.* Model-based Analysis of ChIP-Seq (MACS). *Genome Biology* **9,** R137. ISSN: 1465-6906 (2008).

28. Li, Q., Brown, J. B., Huang, H. & Bickel, P. J. Measuring reproducibility of high-throughput experiments. *Annals of Applied Statistics* **5,** 1752–1779. ISSN: 19326157 (2011).

29. Ramírez, F., Dündar, F., Diehl, S., Grüning, B. A. & Manke, T. DeepTools: A flexible platform for exploring deep-sequencing data. *Nucleic Acids Research* **42.** ISSN: 13624962. doi:10.1093/nar/gku365 (2014).

30. Zhou, X. *et al. The human epigenome browser at Washington University* 2011. doi:10.1038/nmeth.1772. arXiv:NIHMS150003. <http://www.nature.com/doifinder/10.1038/nmeth.1772>.

31. Griffiths-Jones, S., Saini, H. K., Van Dongen, S. & Enright, A. J. miRBase: Tools for microRNA genomics. *Nucleic Acids Research* **36.** ISSN: 03051048. doi:10.1093/nar/gkm952 (2008).

32. Bardet, A. F., He, Q., Zeitlinger, J. & Stark, A. A computational pipeline for comparative ChIP-seq analyses. *Nature Protocols* **7,** 45–61. ISSN: 1754-2189 (2011).

33. Guo, A. Y. *et al.* PlantTFDB: A comprehensive plant transcription factor database. *Nucleic Acids Research* **36.** ISSN: 03051048. doi:10.1093/nar/gkm841 (2008).

34. Pérez-Rodríguez, P. *et al.* PlnTFDB: Updated content and new features of the plant transcription factor database. *Nucleic Acids Research* **38.** ISSN: 03051048. doi:10.1093/nar/gkp805 (2009).

35. Wilson, D., Charoensawan, V., Kummerfeld, S. K. & Teichmann, S. A. DBD - Taxonomically broad transcription factor predictions: New content and functionality. *Nucleic Acids Research* **36.** ISSN: 03051048. doi:10.1093/nar/gkm964 (2008).

36. Pruneda-Paz, J. L. *et al.* A Genome-Scale Resource for the Functional Characterization of Arabidopsis Transcription Factors. *Cell Reports* **8,** 622–632. ISSN: 22111247 (2014).
