# Supplementary material for: Architecture of gene regulatory networks controlling flower development in Arabidopsis thaliana
Source: Nat Commun. 2018 Oct 31;9:4534. doi: 10.1038/s41467-018-06772-3 (PMC6208445; doi:10.1038/s41467-018-06772-3)

Supplementary Data 6: Examples of predicted AP1- or SEP3-regulated feed-forward loops (FFLs).

Developmentally dynamic FFLs were predicted based on the criteria as described below. Generally, the dynamic regulation of the common target gene could be due to either the expression change of its miRNA regulator(s) or the binding change of its upstream master regulator(s).

The barcharts summerize the number of developmentally dynamic FFLs, by 1) dynamic regulation via miRNAs (MIR), or 2) dynamic regulation in TF binding sites (BS).

The remaining plots for the list of potential AP1- or SEP3-regulated FFLs (n = 135): the left panel showing the regulatory relationship of the corresponding FFL, either in a coherent or an incoherent manner; the middle panel showing the dynamic change of miRNA expression or its TF binding across the three timepoints (i.e., stages 2, 4, and 8); the right panel showing the change for the TF target gene. Any significance in change is marked by a star (*). The significance thresholds are defined in the first FFL.

Note: threshold used in the analysis of developmentally changing FFLs:
1) expression change of miRNA genes by ANOVA: fold-change (FC) > 2 and p-vaule < 0.05
2) expression change of target TF genes by ANOVA: FC > 2 and adjusted p-vaule < 0.05
3) change of TF binding intensity for miRNA genes: FC > 1.5
4) change of TF binding intensity for target genes: FC > 1.5

Developmentally changing FFLs are summerized in the right barcharts

MIR: dynamic regulation by miRNAs
BS: dynamic regulation in TF binding sites

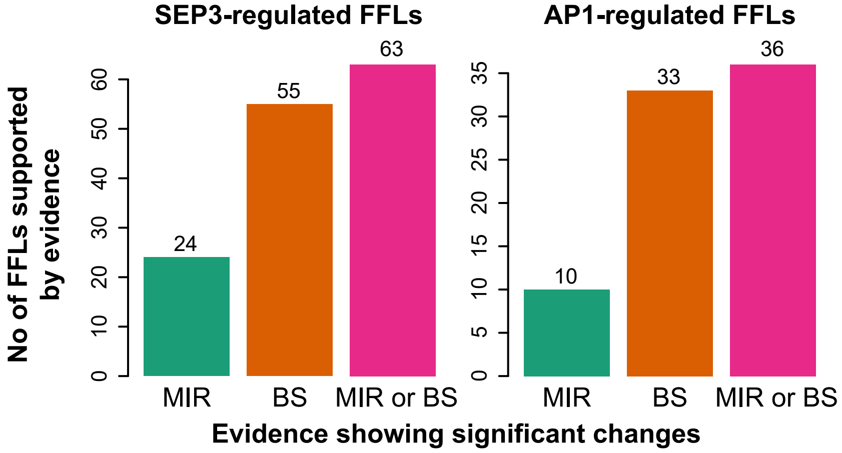

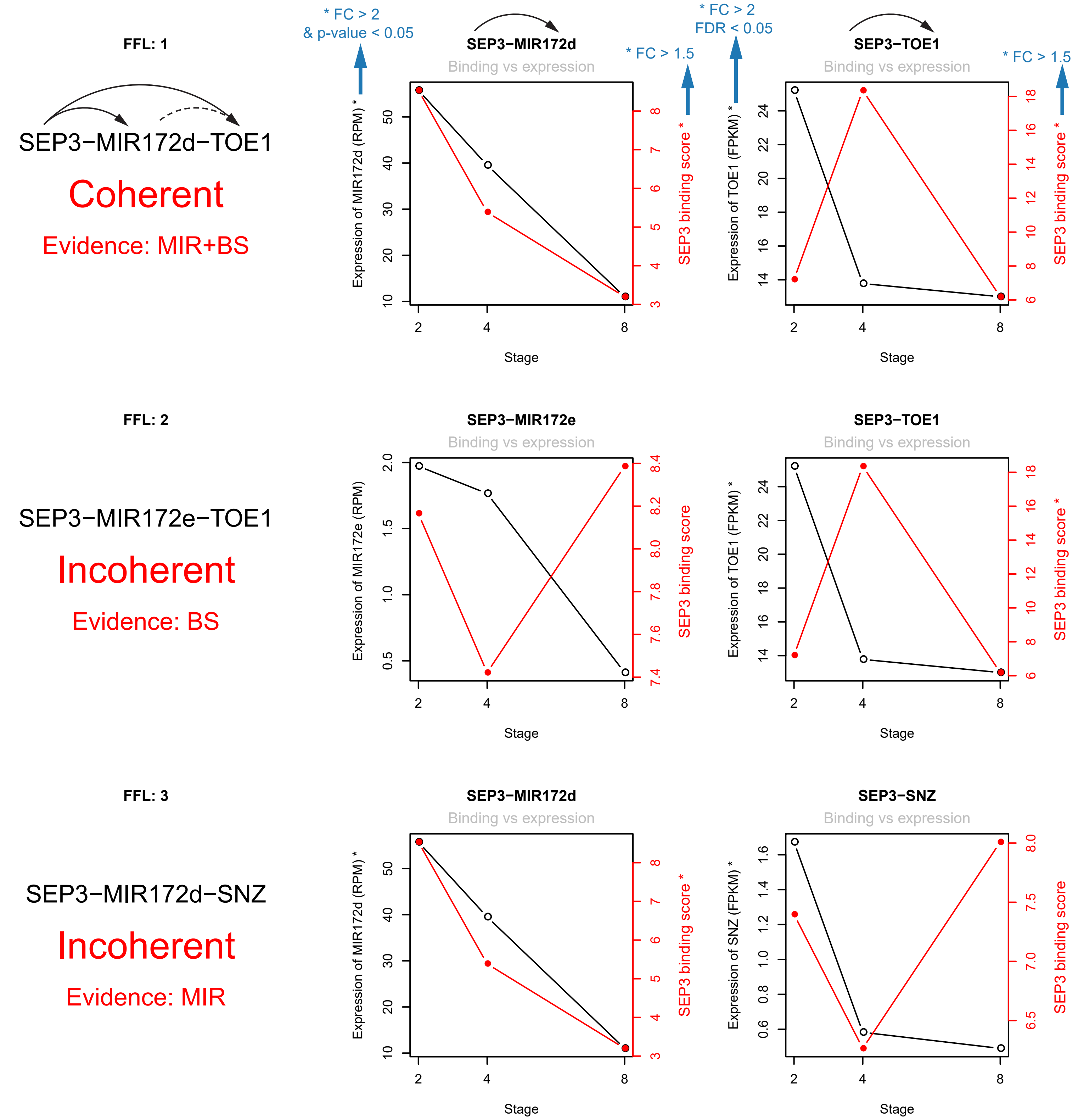

FFL: 4

SEP3−MIR172e−SNZ

Coherent

Evidence:

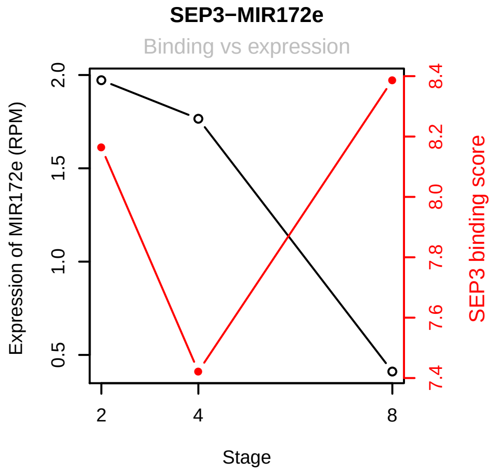

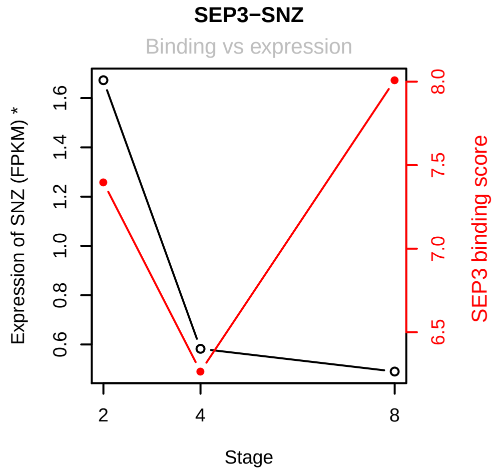

FFL: 5

SEP3−MIR172d−AP2

Coherent

Evidence: MIR+BS

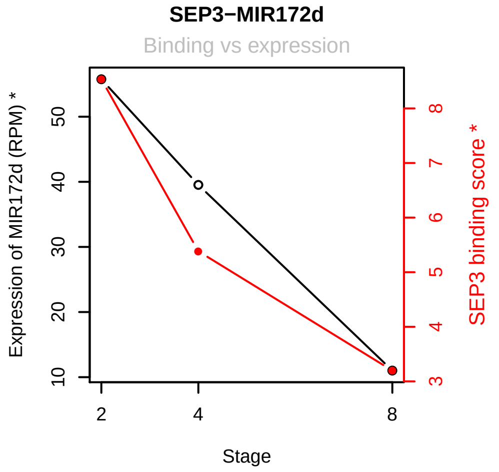

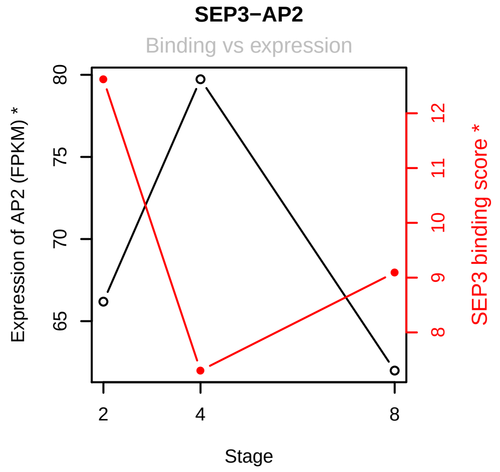

FFL: 6

SEP3−MIR172e−AP2

Incoherent

Evidence: BS

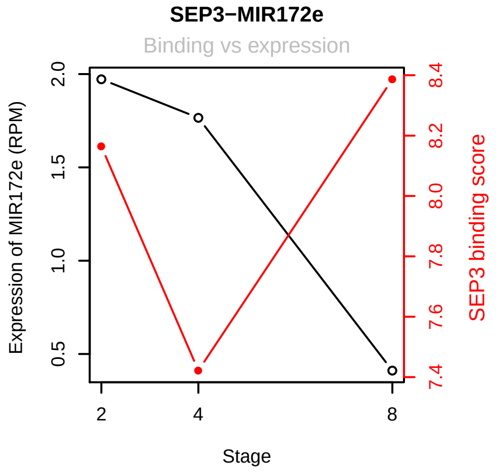

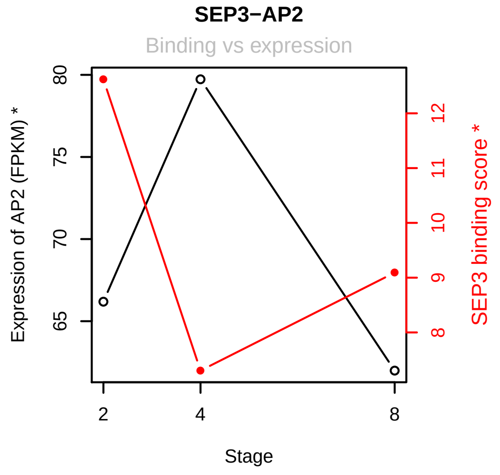

FFL: 7

SEP3−MIR172d−TOE3

Incoherent

Evidence: MIR+BS

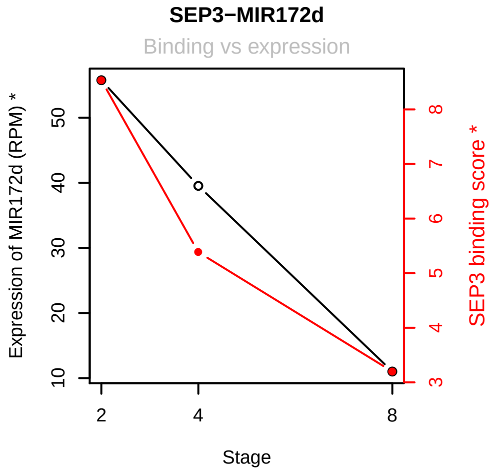

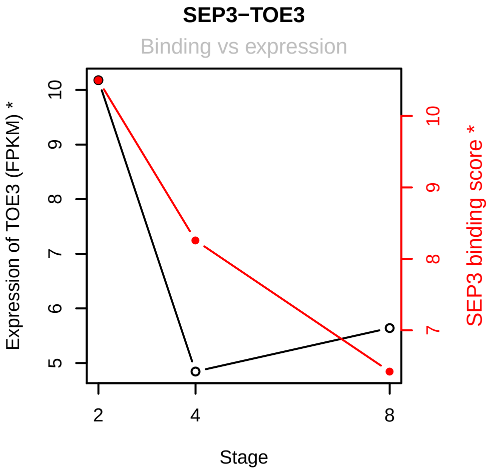

FFL: 8

SEP3−MIR172e−TOE3

Coherent

Evidence: BS

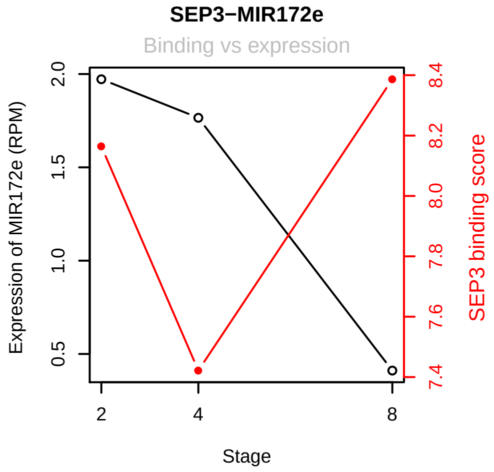

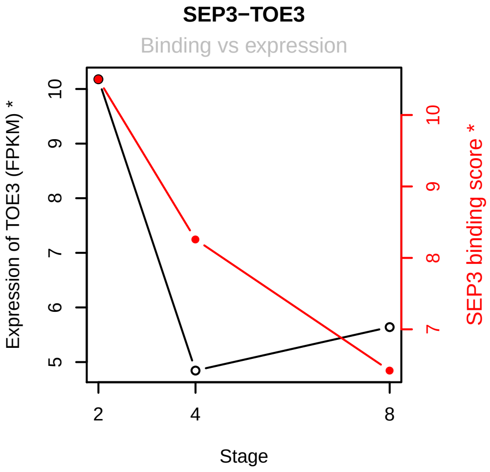

FFL: 9

SEP3−MIR160a−ARF10

Coherent

Evidence:

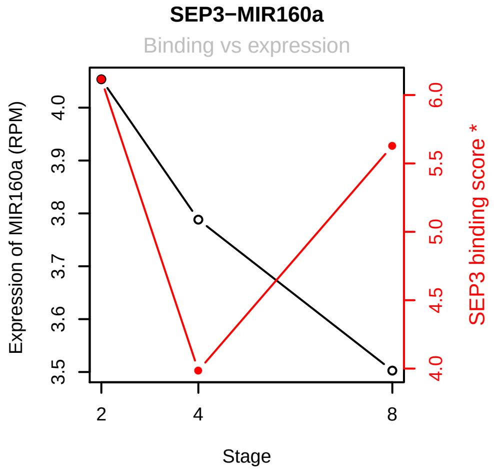

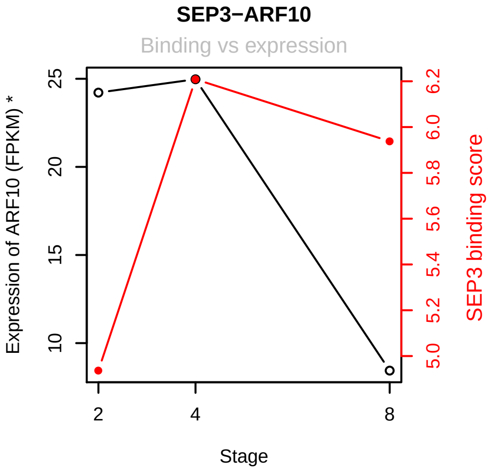

FFL: 10

SEP3−MIR167a−ATARF8

Incoherent

Evidence: MIR+BS

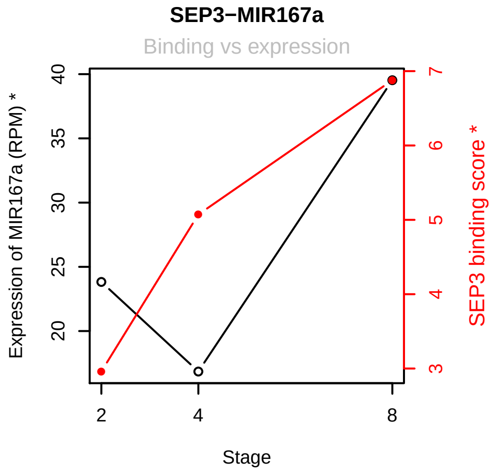

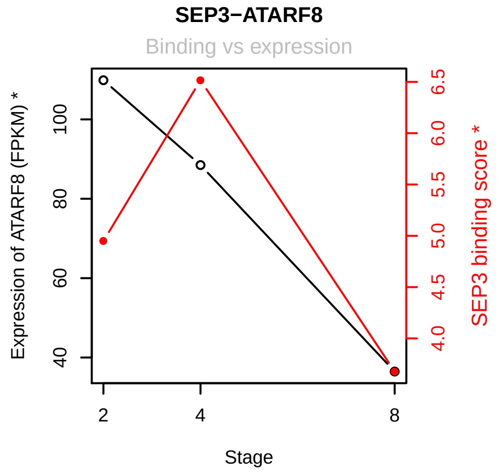

FFL: 11

SEP3−MIR167b−ATARF8

Incoherent

Evidence: BS

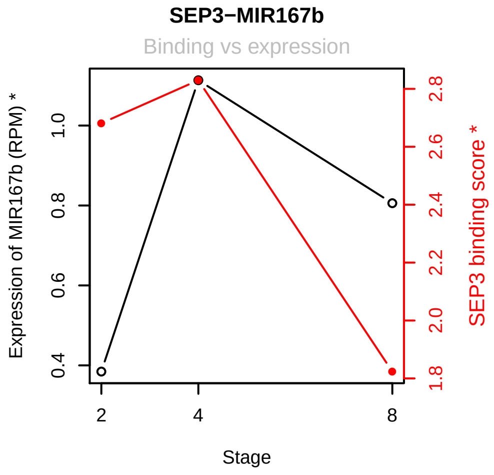

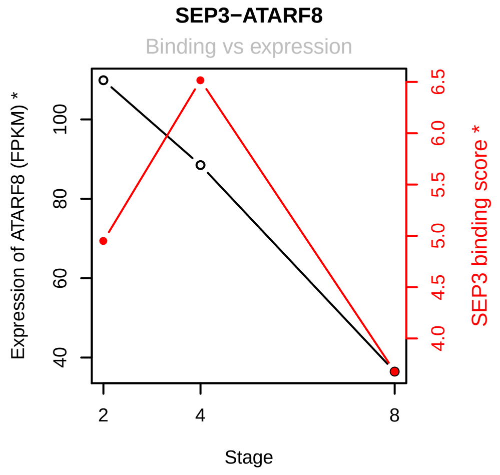

FFL: 12

SEP3−MIR847−EGL3

Coherent

Evidence: BS

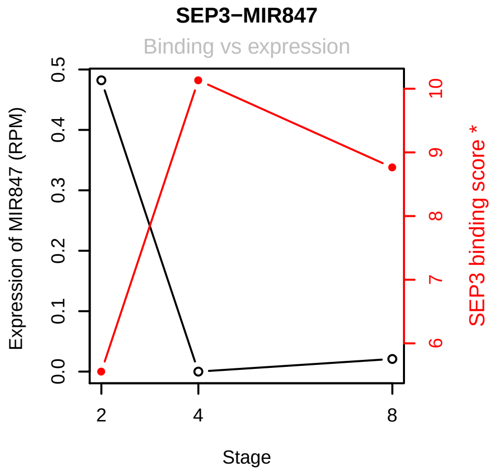

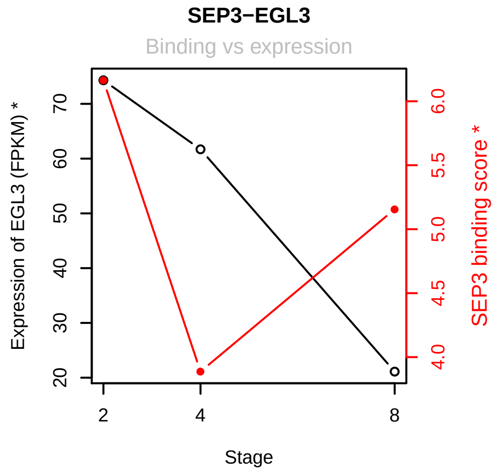

FFL: 13

SEP3−MIR172e−BZIP34

Incoherent

Evidence:

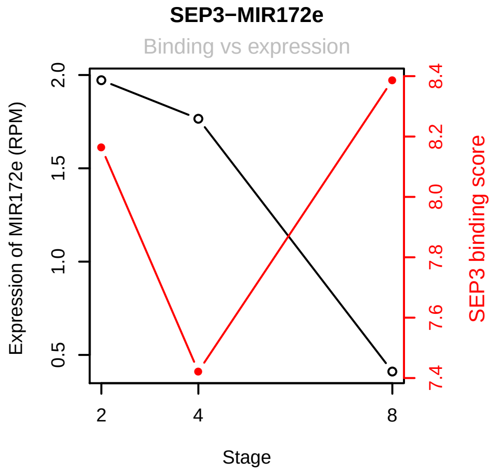

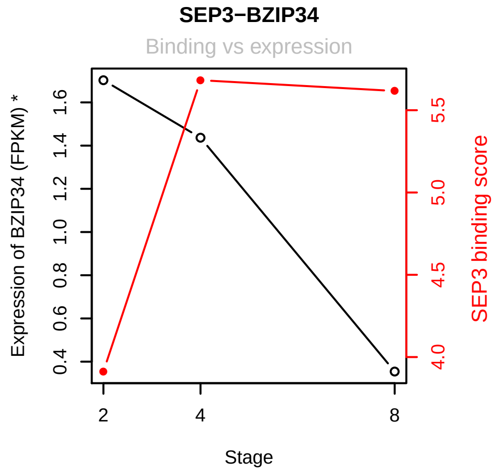

FFL: 14

SEP3−MIR847−COL1

Coherent

Evidence: BS

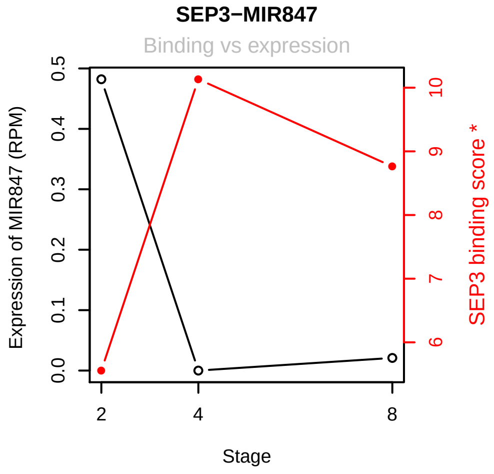

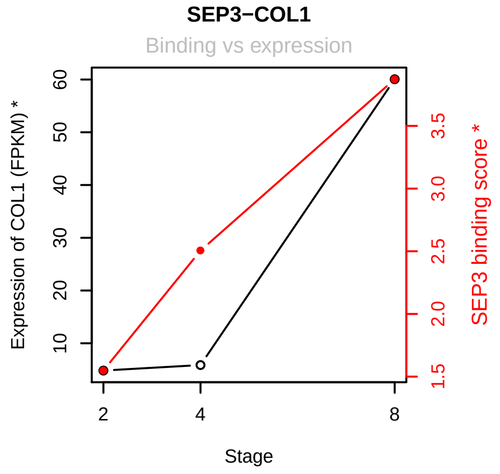

FFL: 15

SEP3−MIR169i−NF−YA10

Incoherent

Evidence: BS

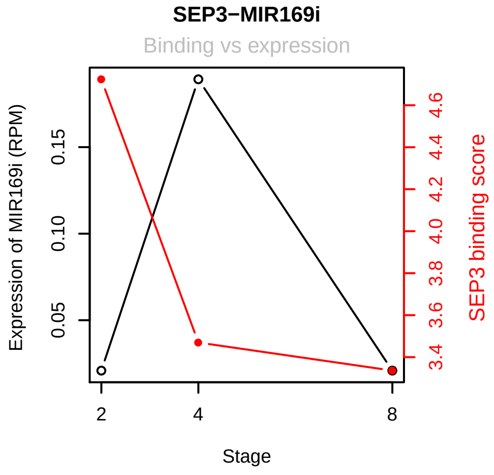

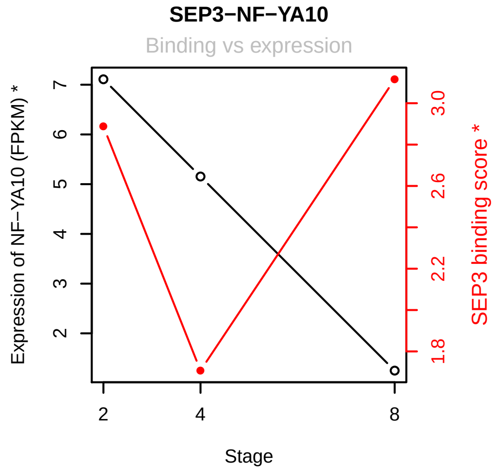

FFL: 16

SEP3−MIR170−SCL6−IV

Incoherent

Evidence: BS

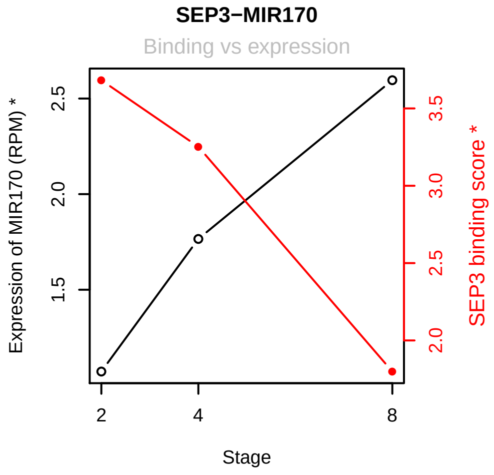

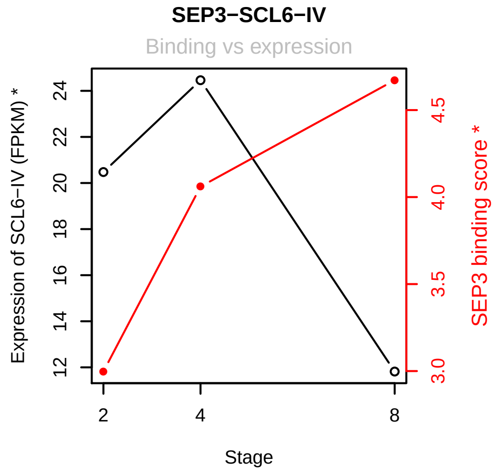

FFL: 17

SEP3−MIR171a−SCL6−IV

Incoherent

Evidence: MIR+BS

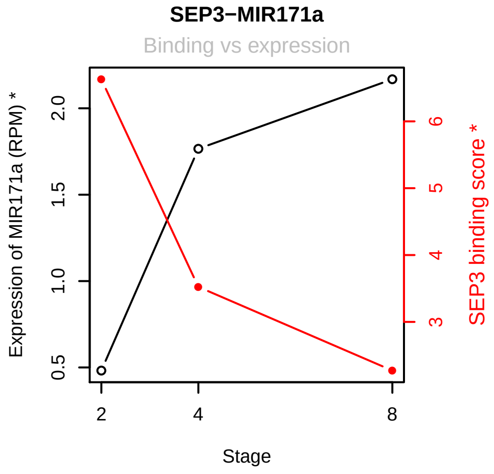

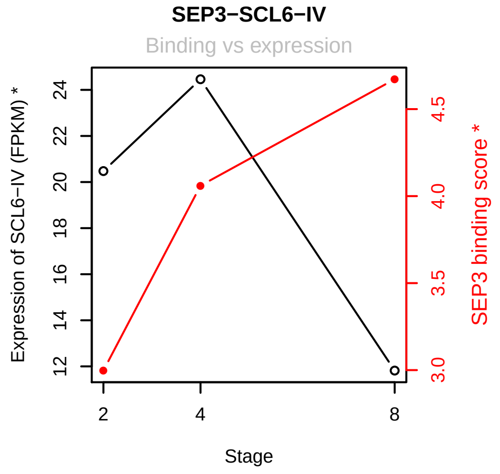

FFL: 18

SEP3−MIR171b−SCL6−IV

Incoherent

Evidence: BS

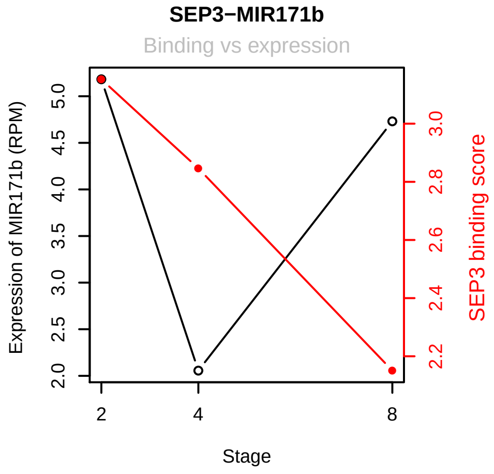

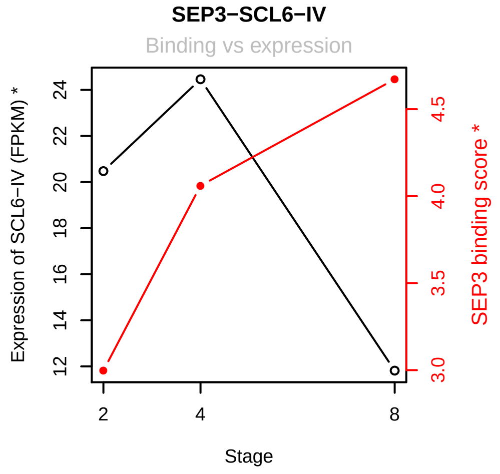

FFL: 19

SEP3−MIR396b−GRF9

Coherent

Evidence: MIR+BS

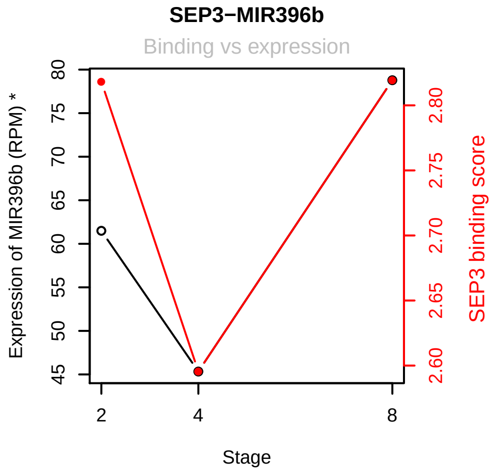

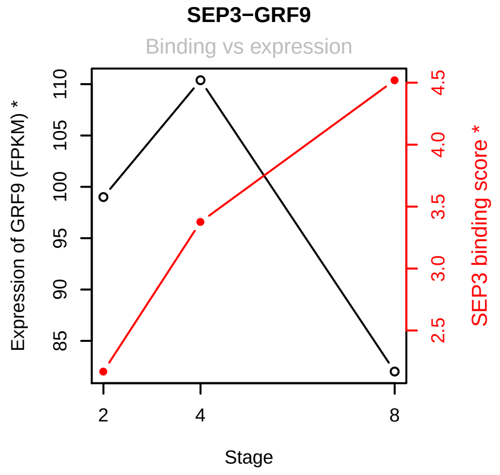

FFL: 20

SEP3−MIR396b−GRF4

Incoherent

Evidence: MIR+BS

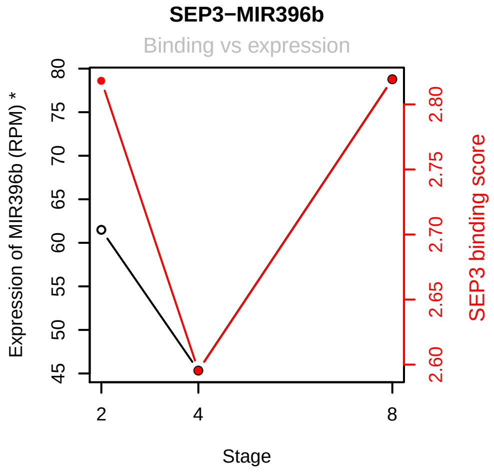

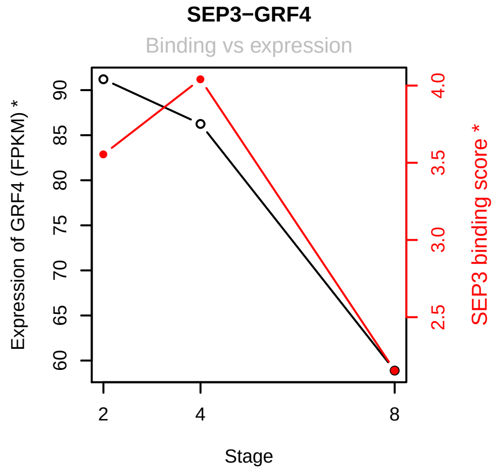

FFL: 21

SEP3−MIR396b−GRF8

Incoherent

Evidence: MIR+BS

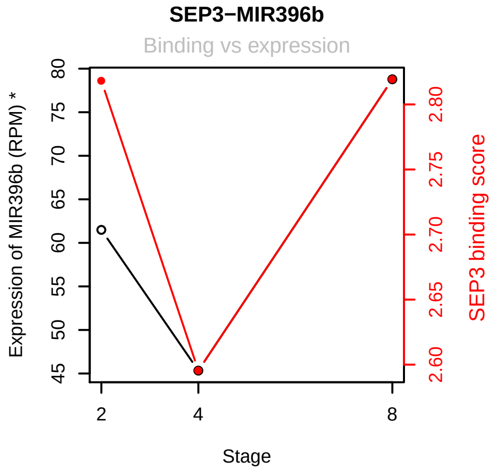

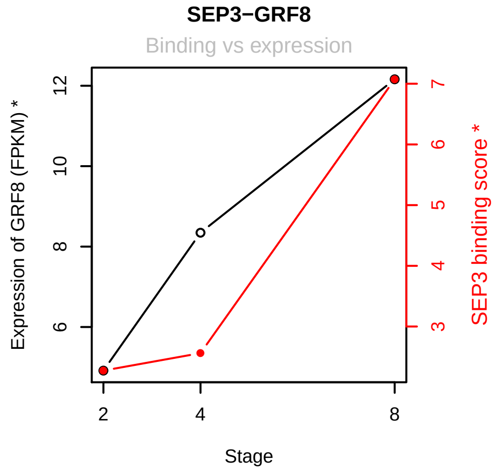

FFL: 22

SEP3−MIR396b−GRF7

Coherent

Evidence: MIR+BS

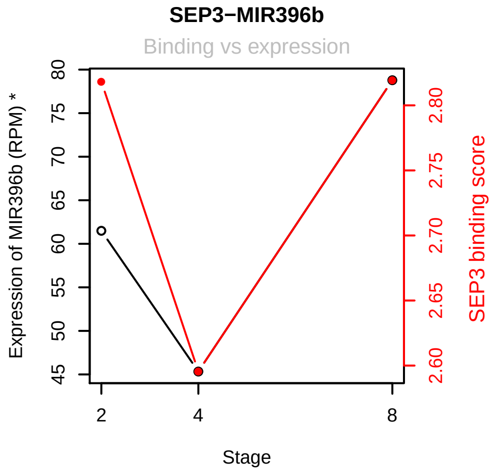

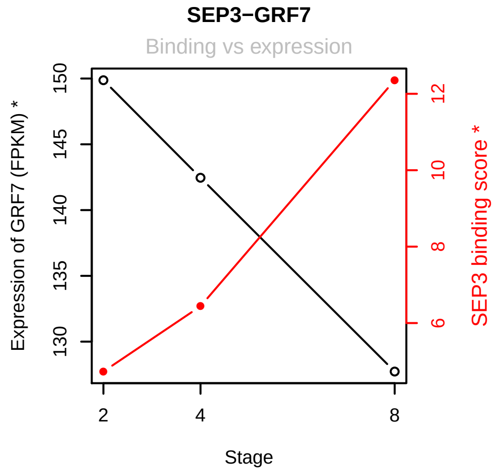

FFL: 23

SEP3−MIR165a−PHB−1D

Incoherent

Evidence:

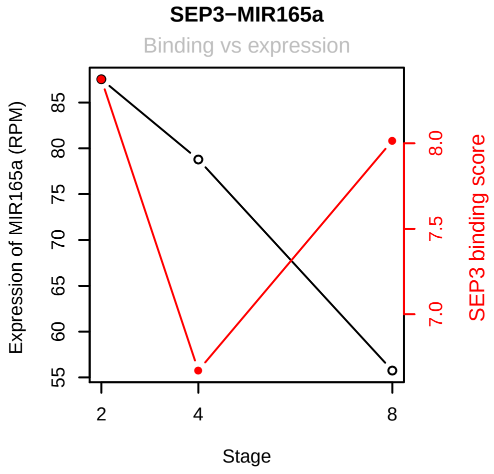

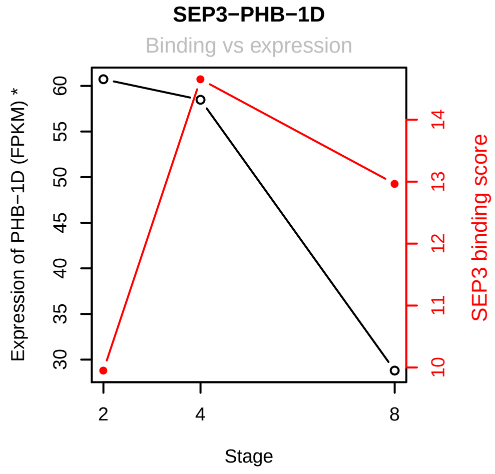

FFL: 24

SEP3−MIR166a−PHB−1D

Coherent

Evidence:

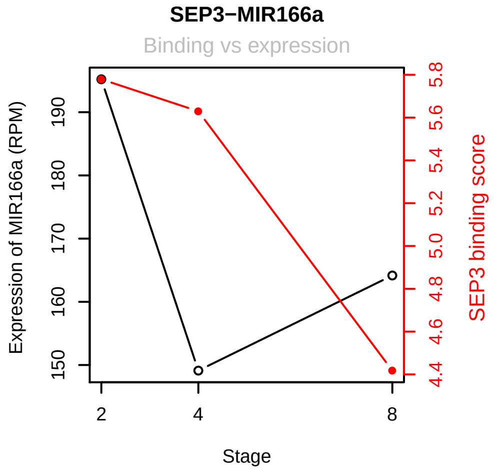

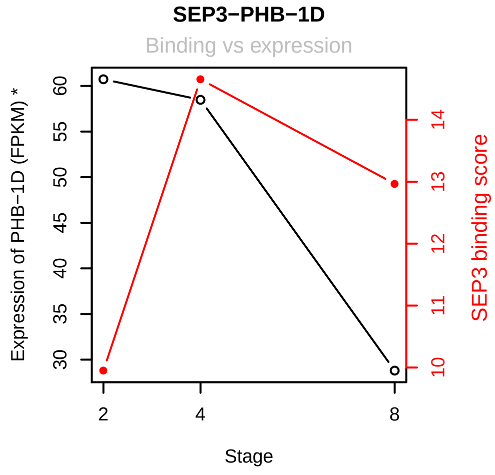

FFL: 25

SEP3−MIR166b−PHB−1D

Incoherent

Evidence:

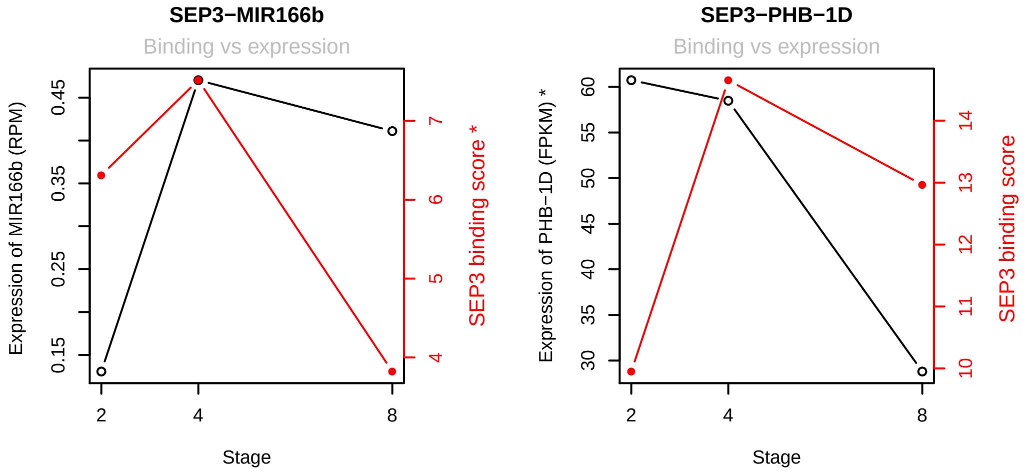

FFL: 26

SEP3−MIR166c−PHB−1D

Incoherent

Evidence:

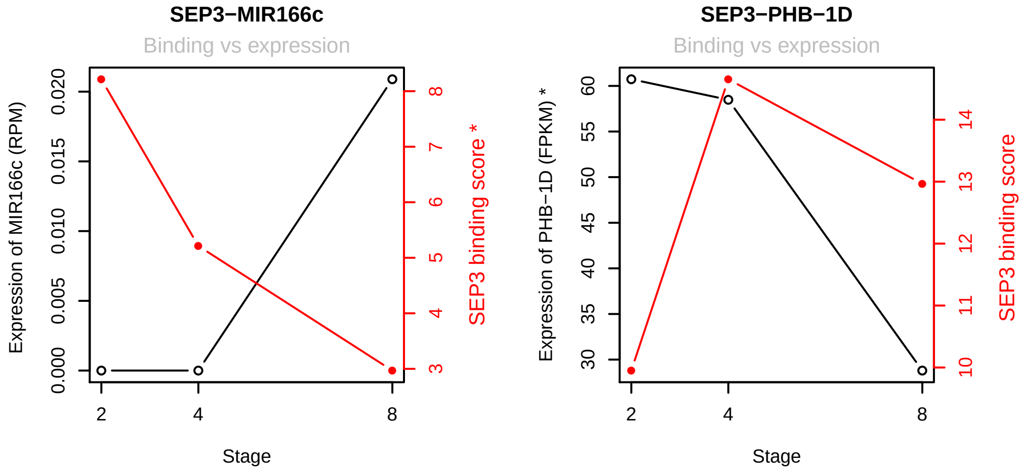

FFL: 27

SEP3−MIR165a−HB−8

Coherent

Evidence: BS

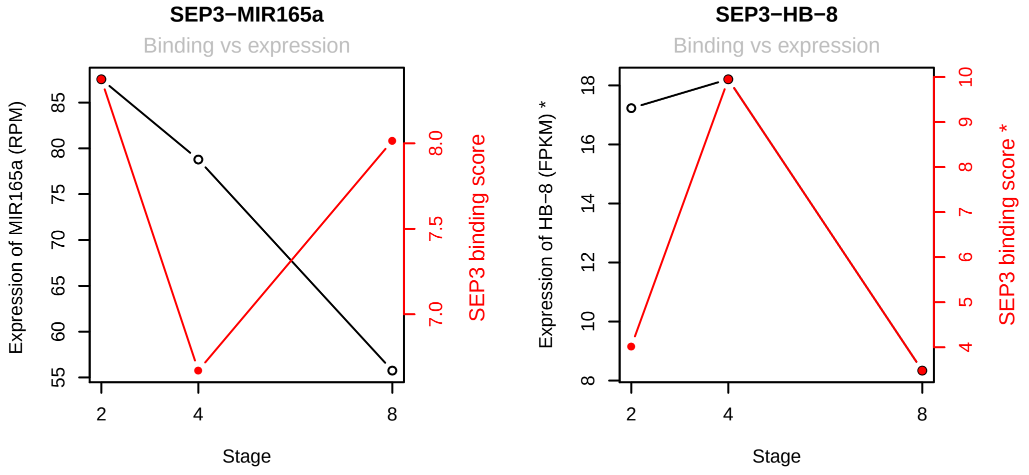

FFL: 28

SEP3−MIR166a−HB−8

Incoherent

Evidence: BS

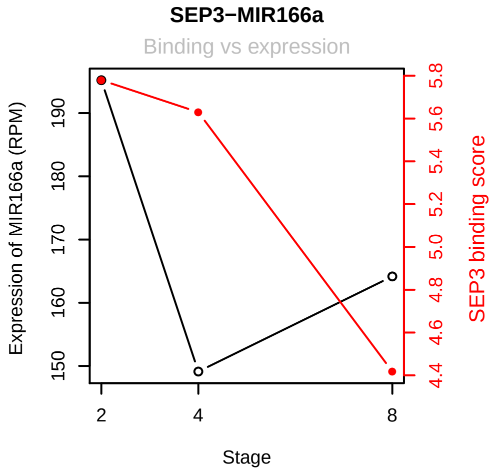

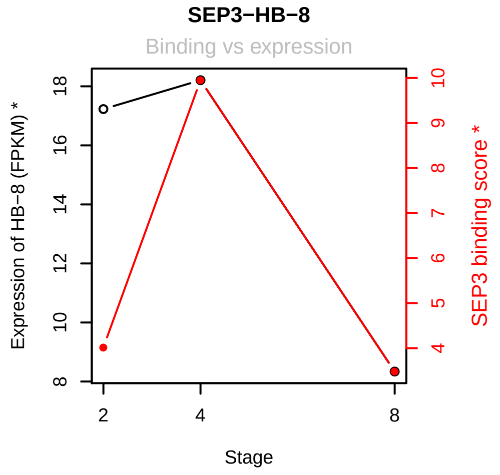

FFL: 29

SEP3−MIR166b−HB−8

Coherent

Evidence: BS

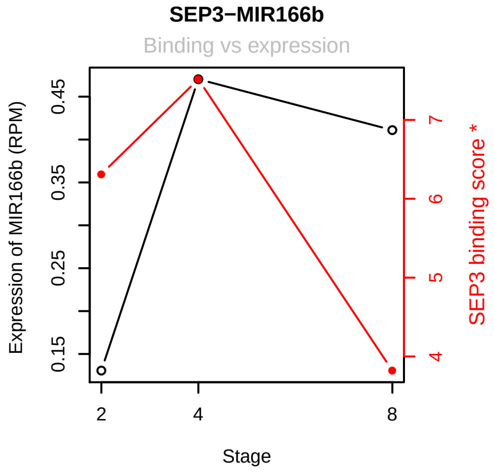

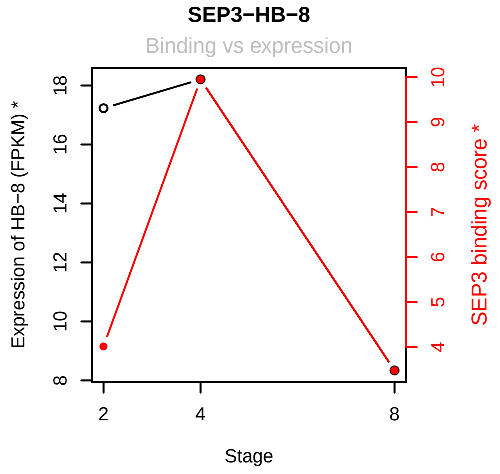

FFL: 30

SEP3−MIR166c−HB−8

Coherent

Evidence: BS

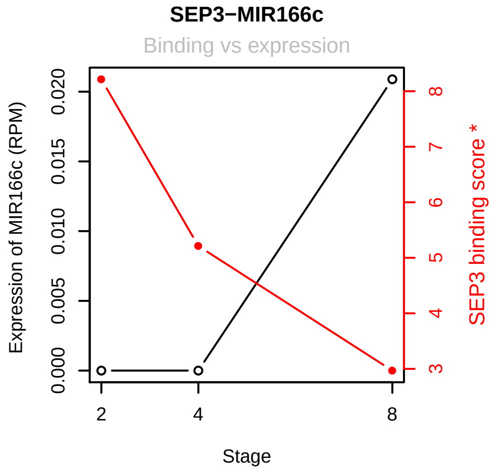

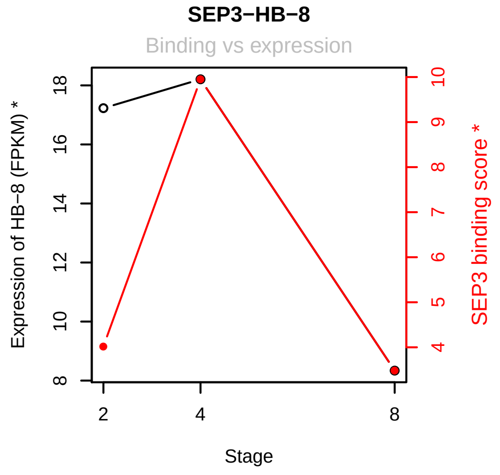

FFL: 31

SEP3−MIR159b−SHP1

Coherent

Evidence:

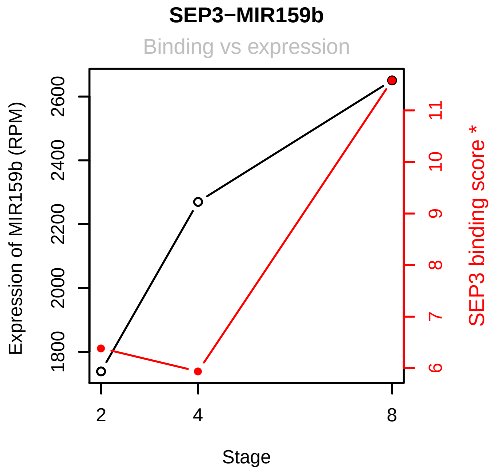

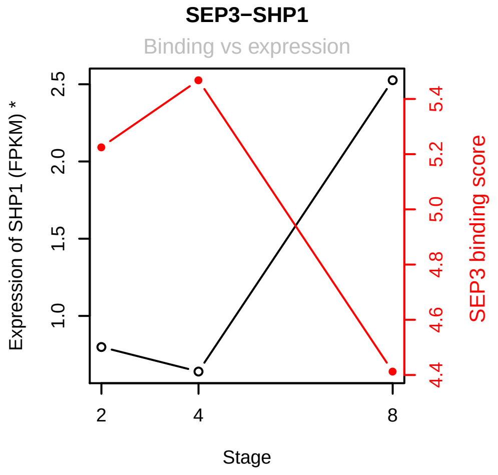

FFL: 32

SEP3−MIR858a−MYB13

Incoherent

Evidence: MIR

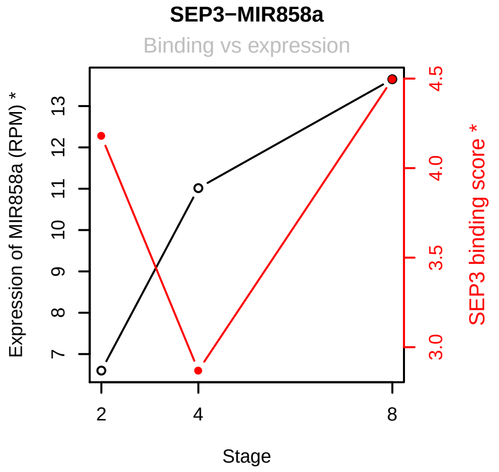

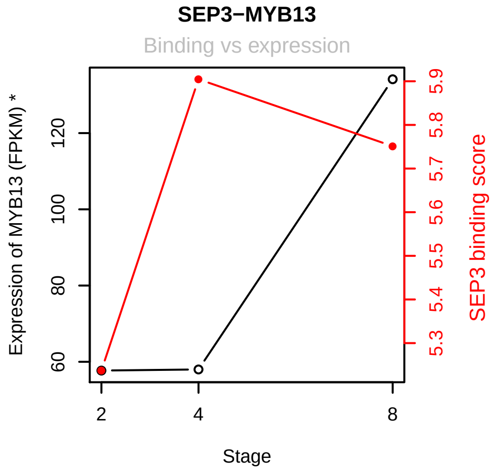

FFL: 33

SEP3−MIR858a−PFG2

Incoherent

Evidence: MIR

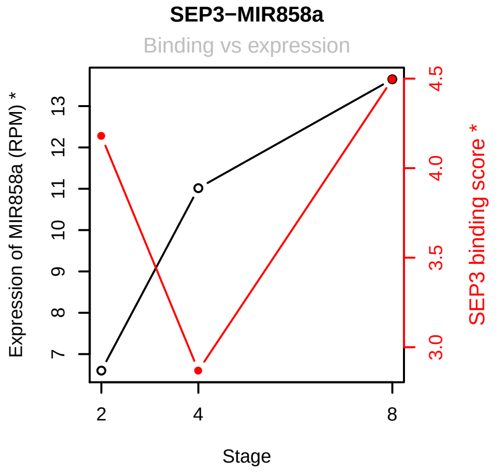

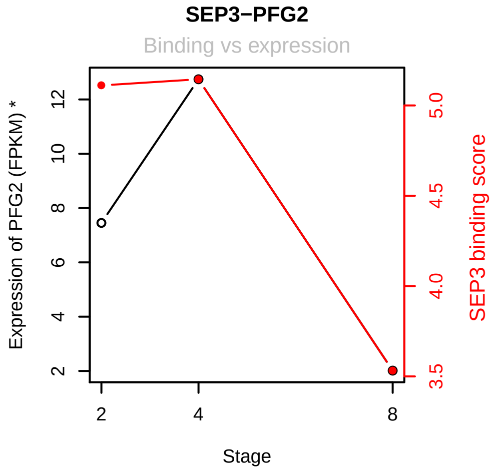

FFL: 34

SEP3−MIR164b−CUC1

Coherent

Evidence: MIR+BS

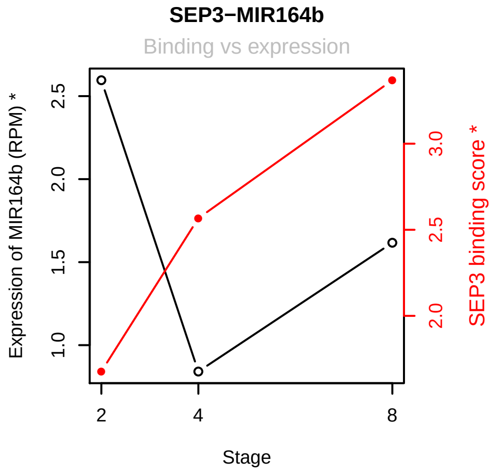

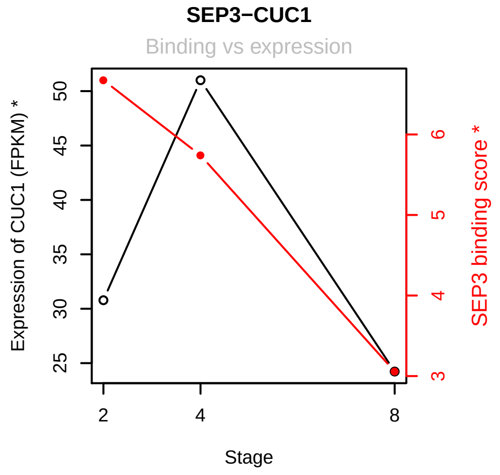

FFL: 35

SEP3−MIR164b−CUC2

Coherent

Evidence: MIR+BS

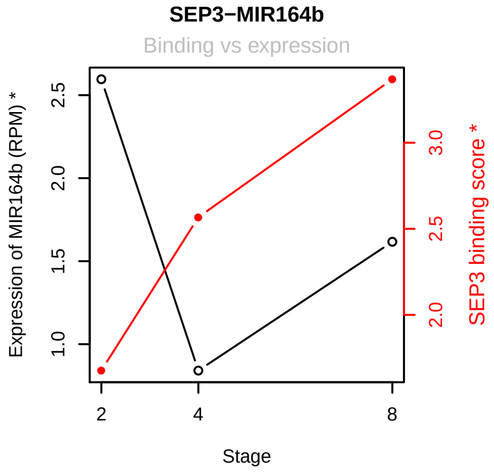

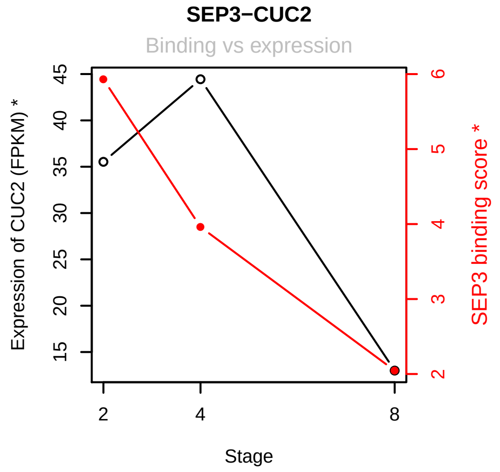

FFL: 36

SEP3−MIR159b−SPL

Incoherent

Evidence:

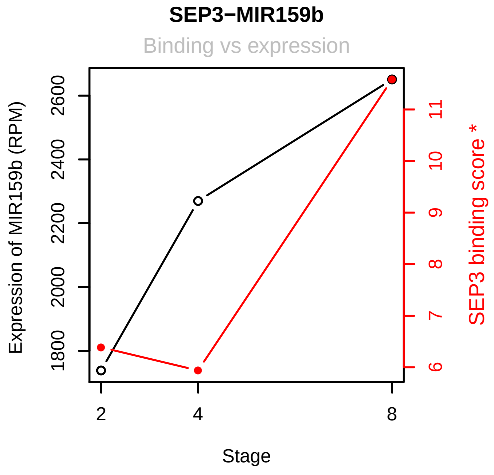

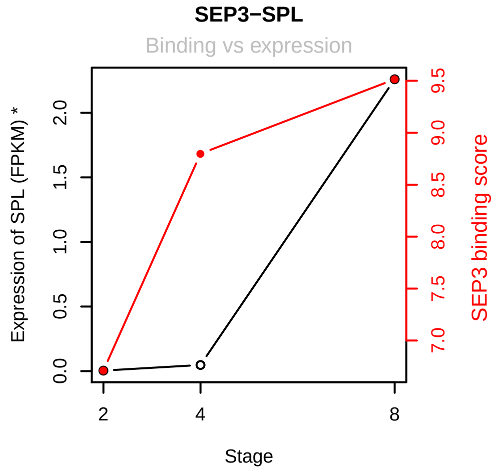

FFL: 37

SEP3−MIR159c−SPL

Coherent

Evidence: MIR

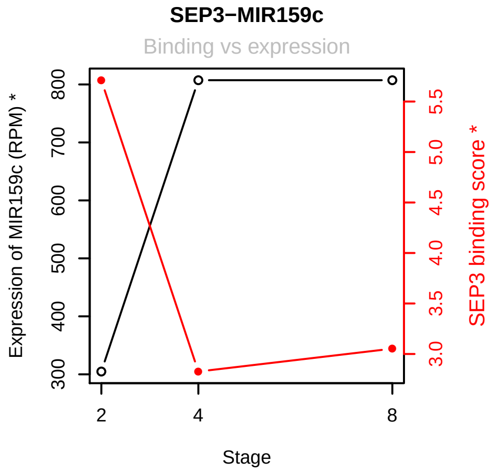

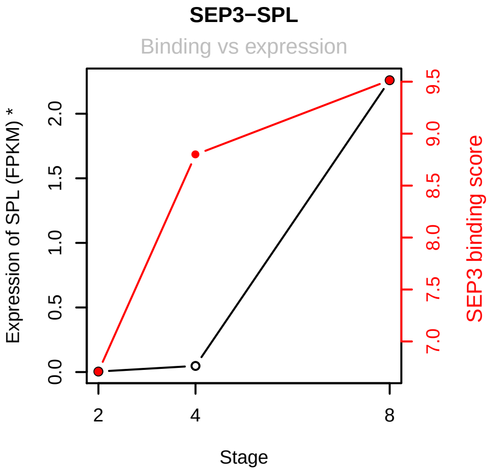

FFL: 38

SEP3−MIR156a−SPL11

Incoherent

Evidence:

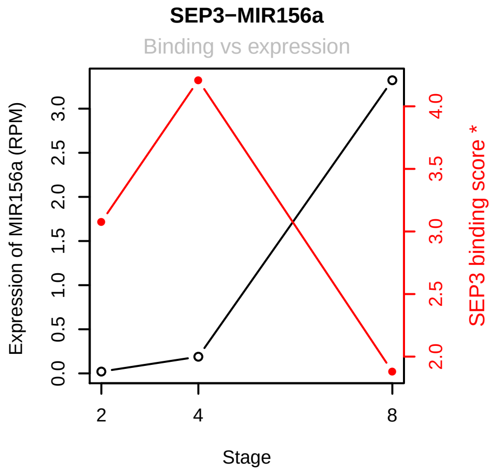

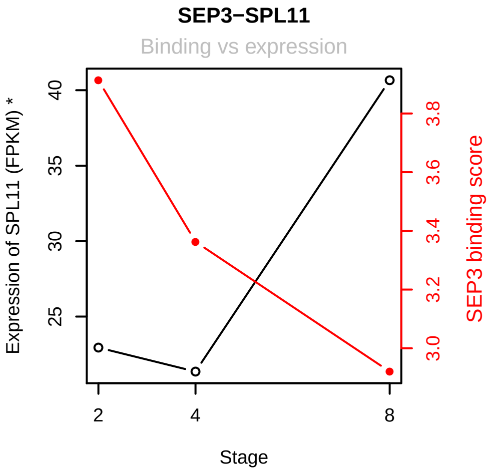

FFL: 39

SEP3−MIR156b−SPL11

Coherent

Evidence:

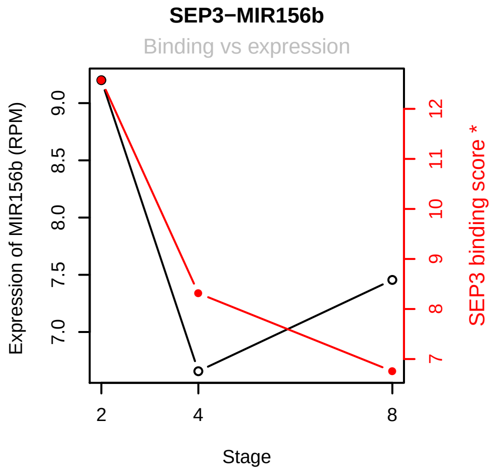

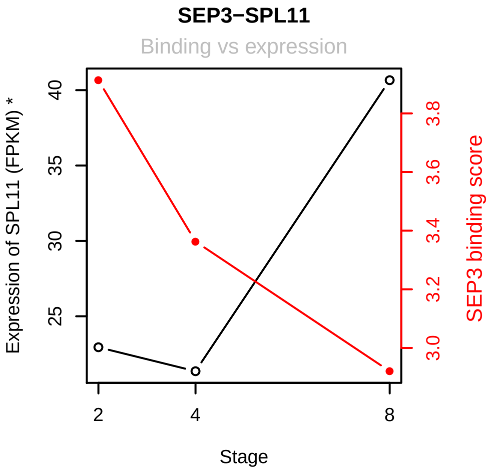

FFL: 40

SEP3−MIR156c−SPL11

Incoherent

Evidence:

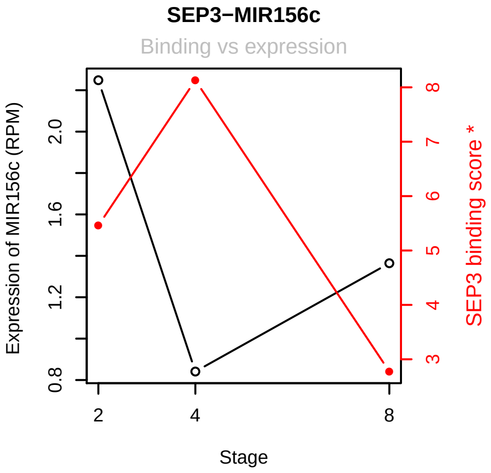

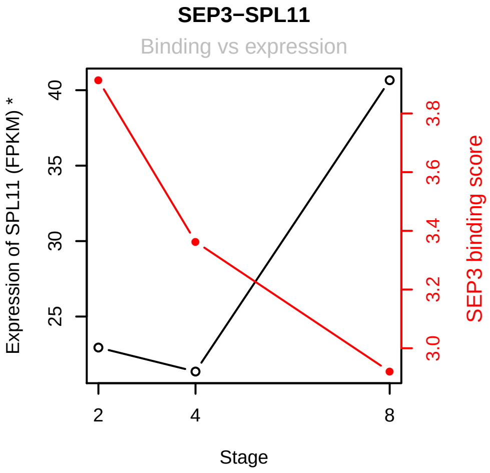

FFL: 41

SEP3−MIR156d−SPL11

Coherent

Evidence:

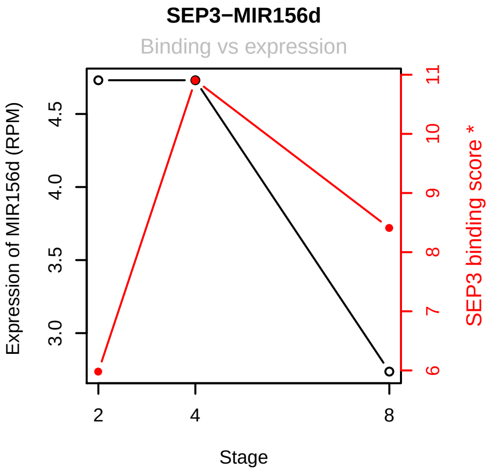

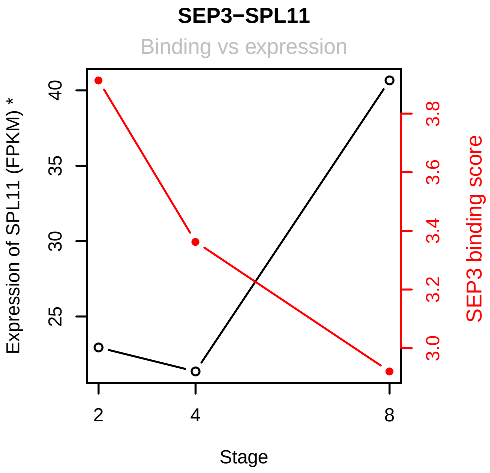

FFL: 42

SEP3−MIR156j−SPL11

Incoherent

Evidence:

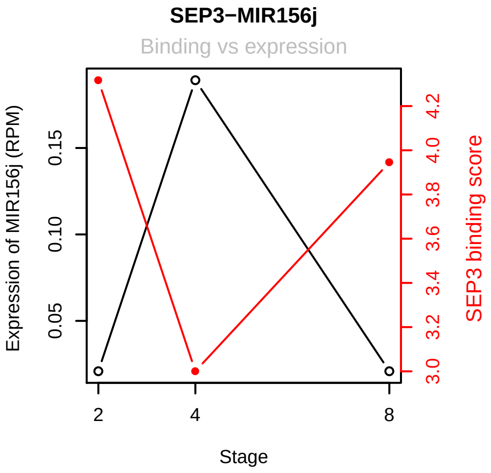

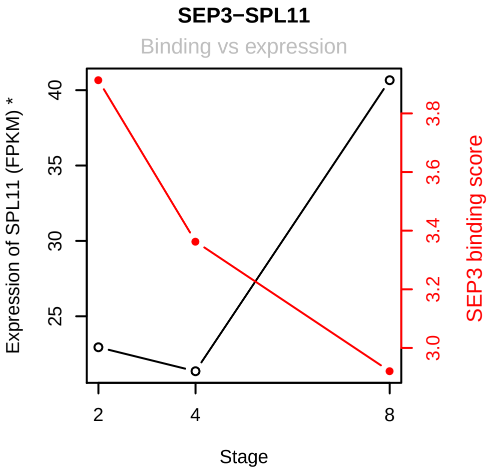

FFL: 43

SEP3−MIR157c−SPL11

Coherent

Evidence:

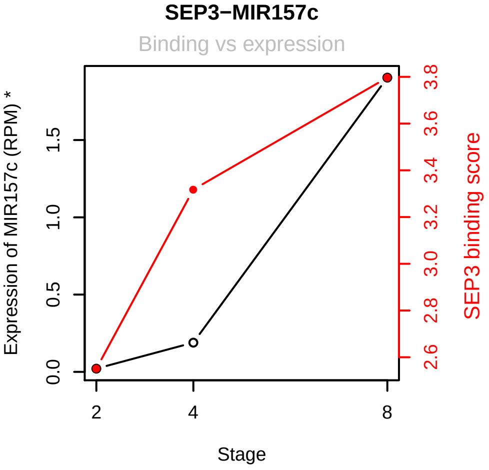

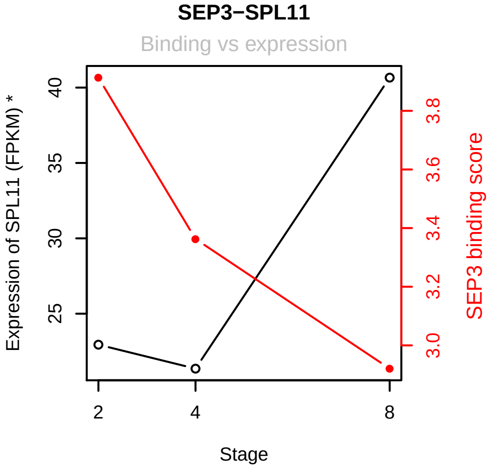

FFL: 44

SEP3−MIR157d−SPL11

Coherent

Evidence: MIR

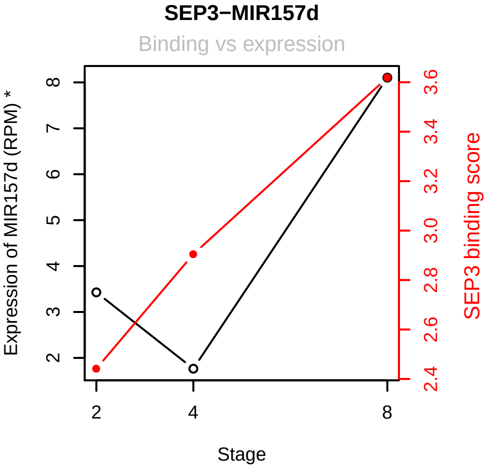

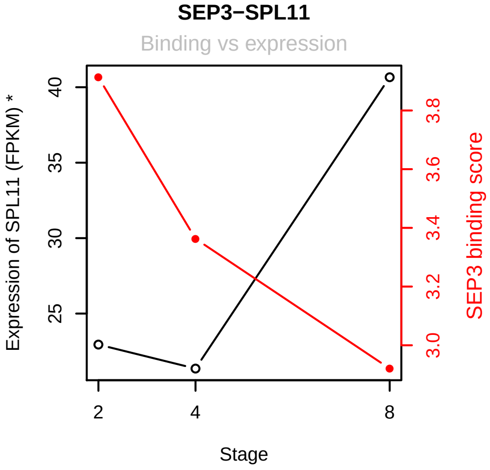

FFL: 45

SEP3−MIR156a−SPL3

Incoherent

Evidence: BS

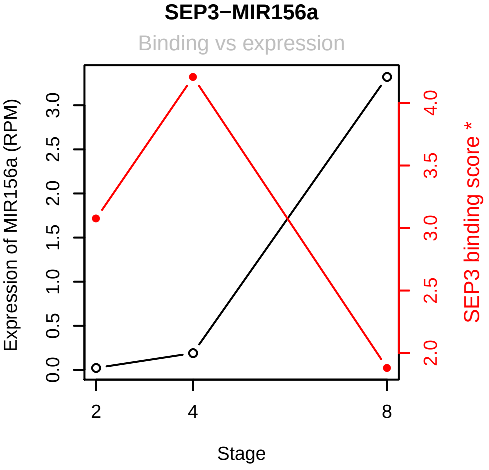

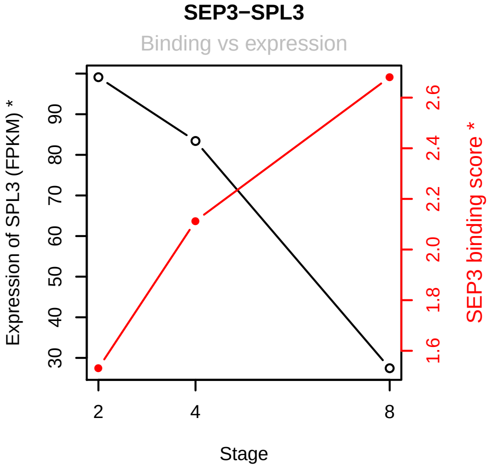

FFL: 46

SEP3−MIR156b−SPL3

Coherent

Evidence: BS

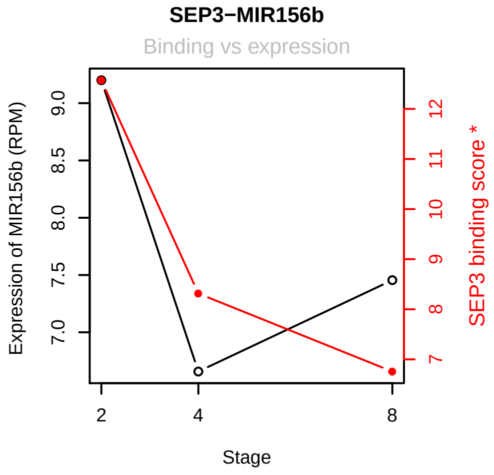

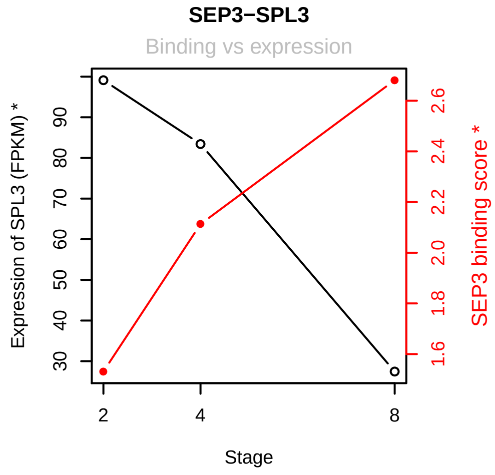

FFL: 47

SEP3−MIR156c−SPL3

Incoherent

Evidence: BS

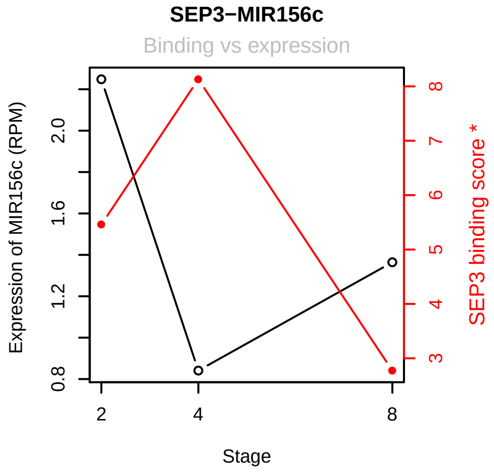

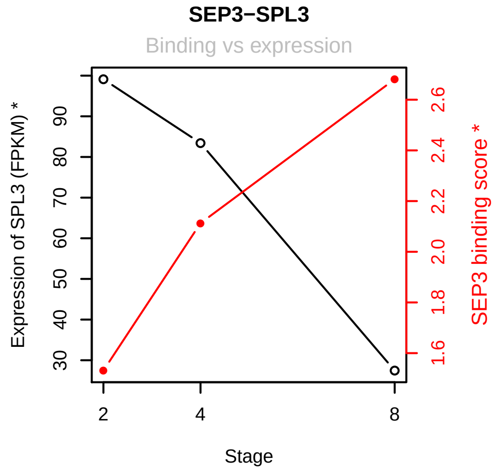

FFL: 48

SEP3−MIR156d−SPL3

Coherent

Evidence: BS

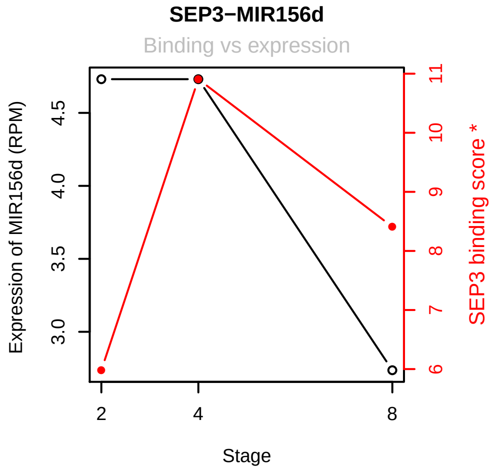

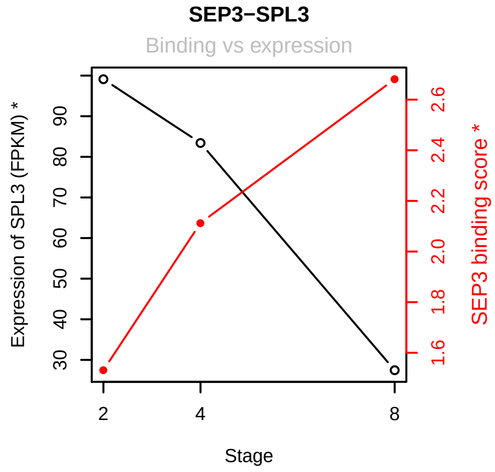

FFL: 49

SEP3−MIR156j−SPL3

Incoherent

Evidence: BS

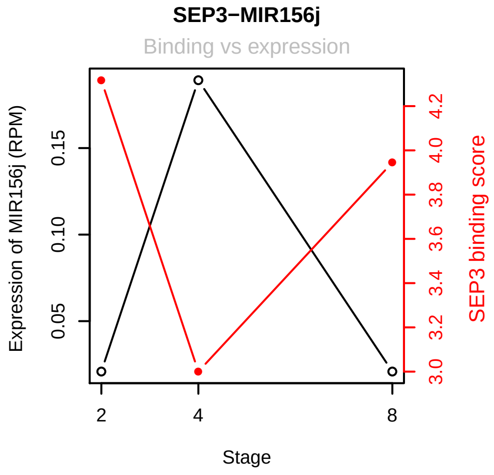

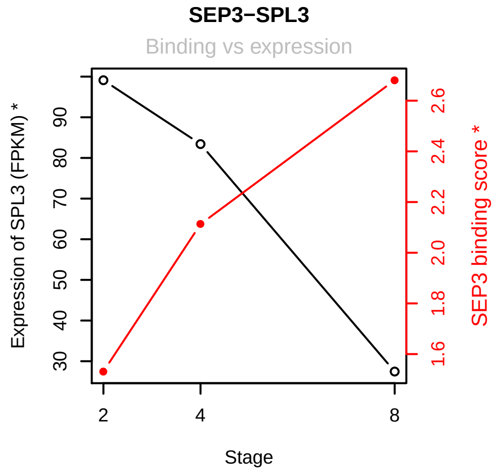

FFL: 50

SEP3−MIR156a−SPL9

Coherent

Evidence:

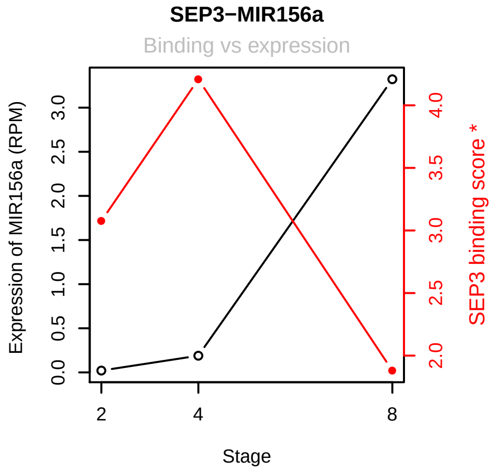

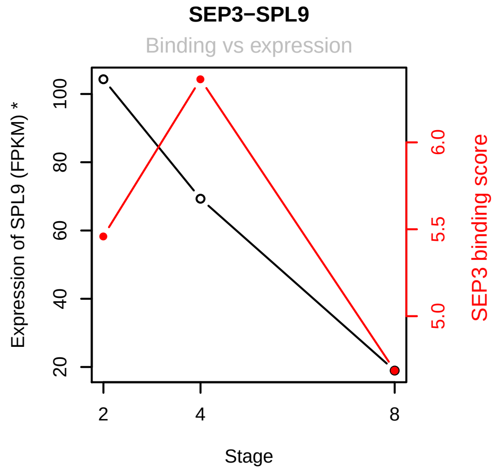

FFL: 51

SEP3−MIR156b−SPL9

Incoherent

Evidence:

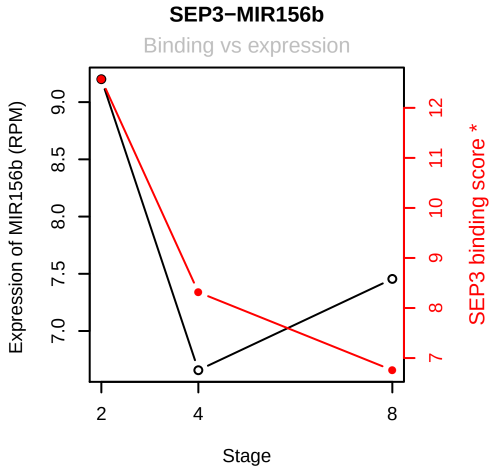

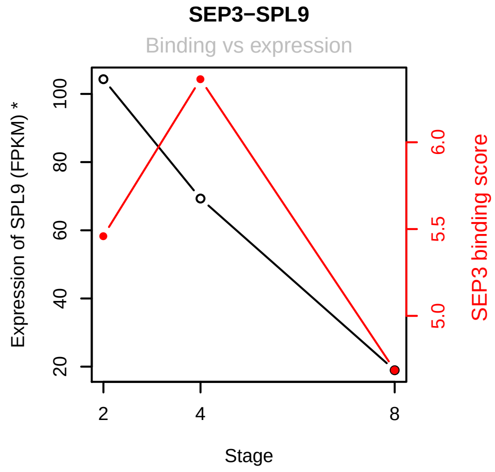

FFL: 52

SEP3−MIR156c−SPL9

Coherent

Evidence:

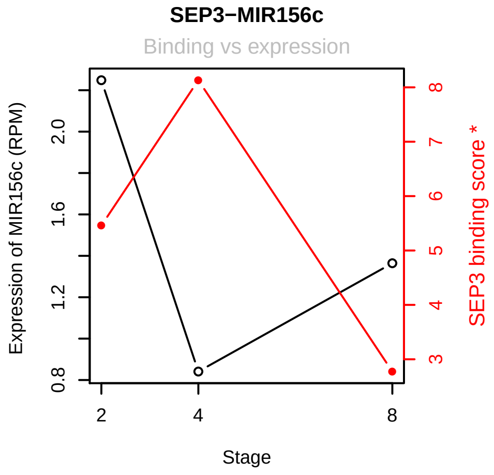

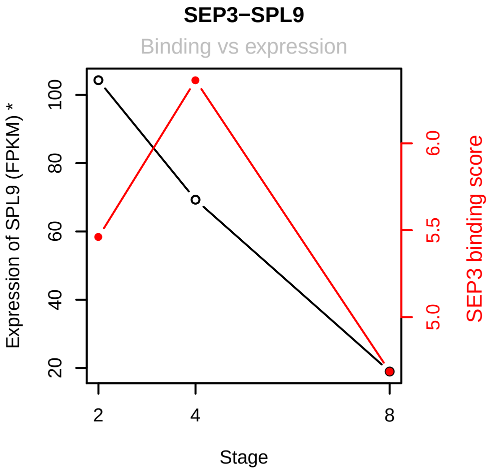

FFL: 53

SEP3−MIR156d−SPL9

Incoherent

Evidence:

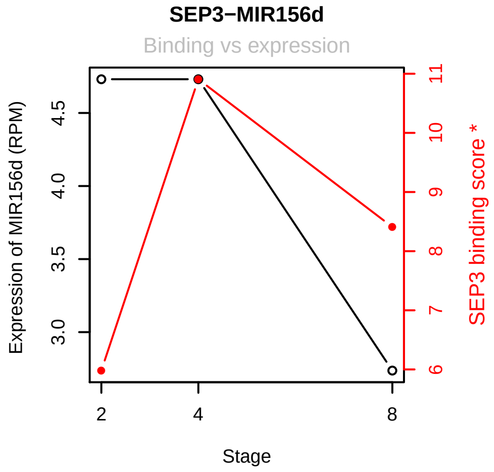

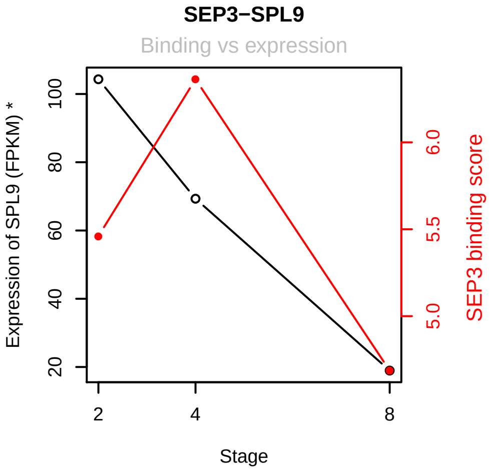

FFL: 54

SEP3−MIR156j−SPL9

Coherent

Evidence:

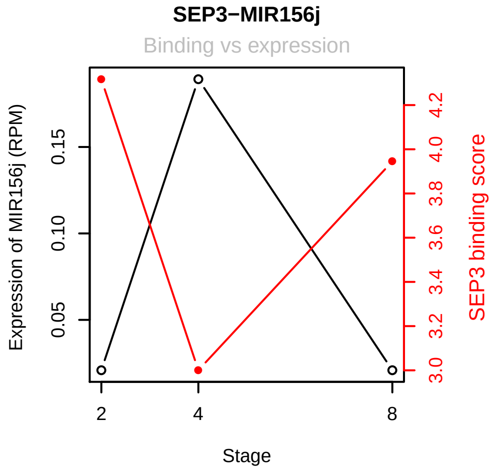

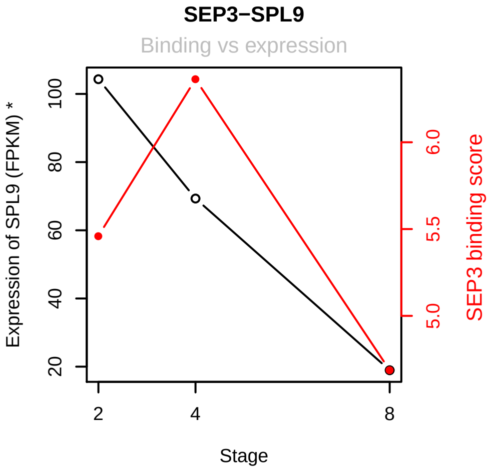

FFL: 55

SEP3−MIR157c−SPL9

Incoherent

Evidence:

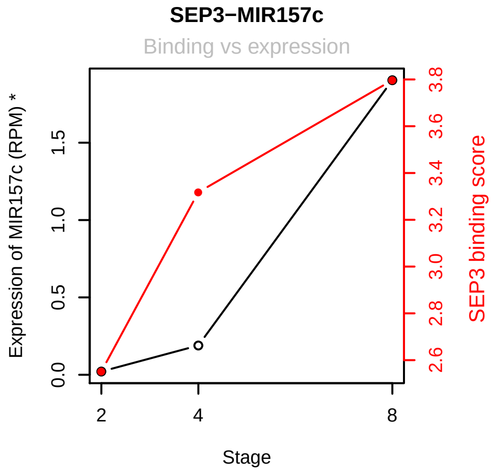

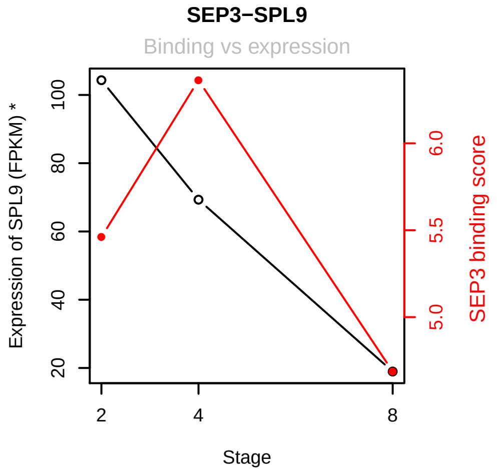

FFL: 56

SEP3−MIR157d−SPL9

Incoherent

Evidence: MIR

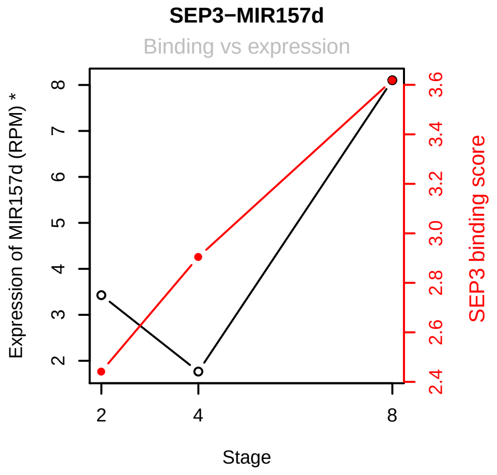

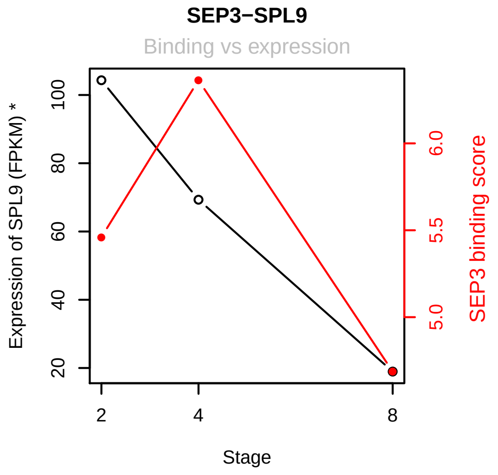

FFL: 57

SEP3−MIR156a−SPL5

Incoherent

Evidence:

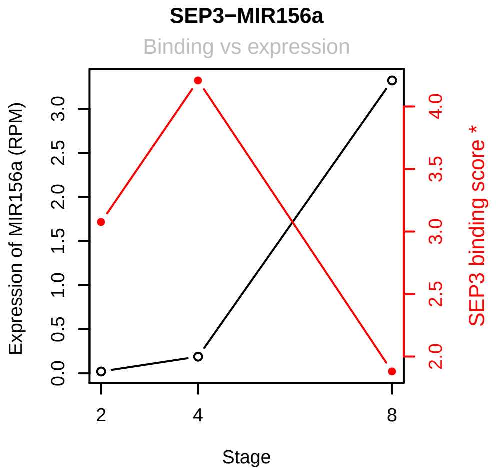

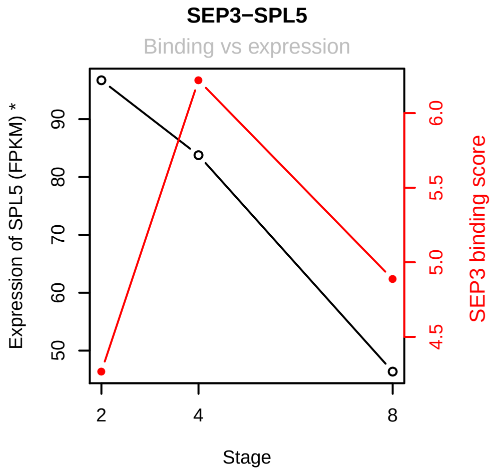

FFL: 58

SEP3−MIR156b−SPL5

Coherent

Evidence:

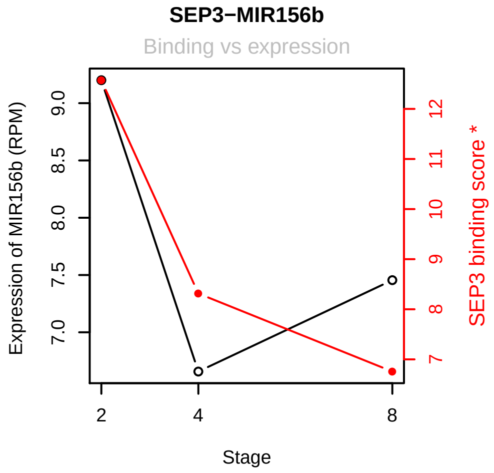

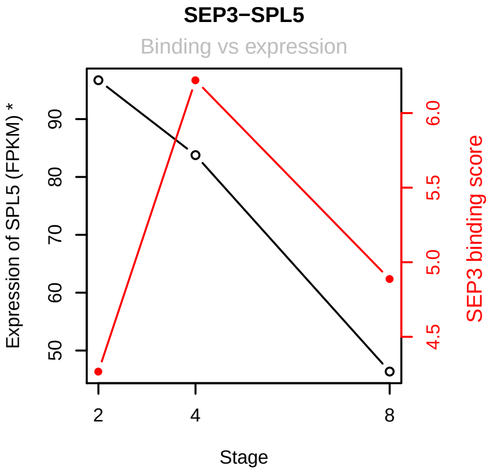

FFL: 59

SEP3−MIR156c−SPL5

Incoherent

Evidence:

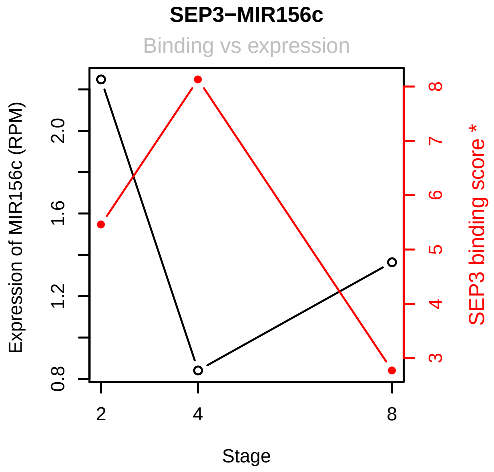

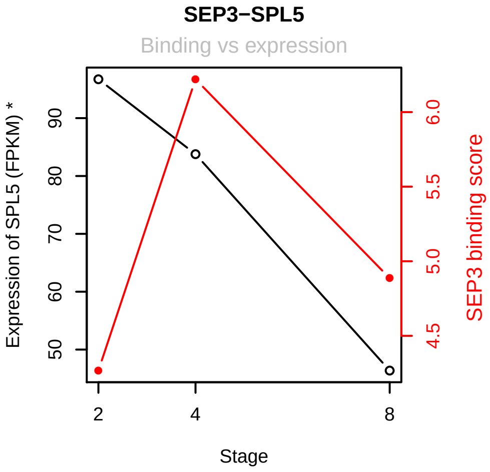

FFL: 60

SEP3−MIR156d−SPL5

Coherent

Evidence:

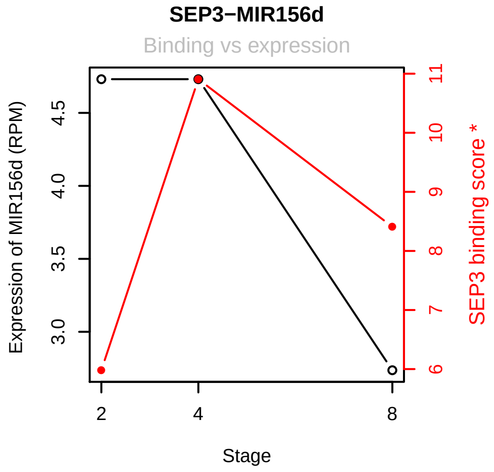

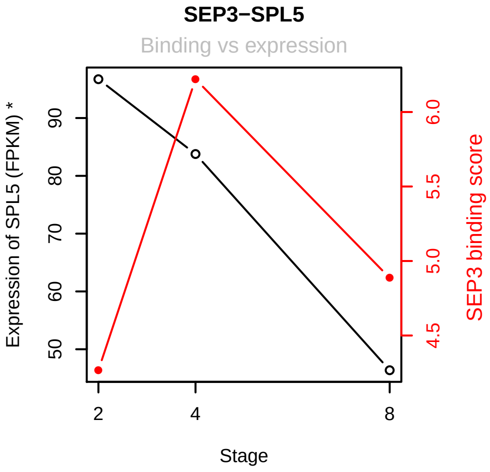

FFL: 61

SEP3−MIR156j−SPL5

Incoherent

Evidence:

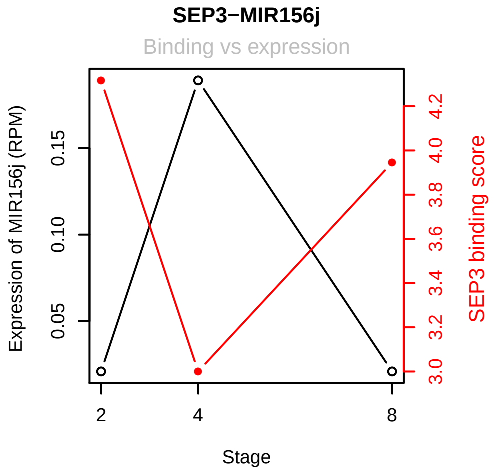

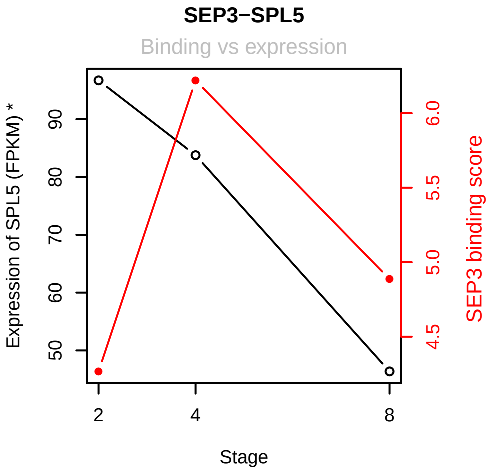

FFL: 62

SEP3−MIR157d−SPL5

Coherent

Evidence: MIR

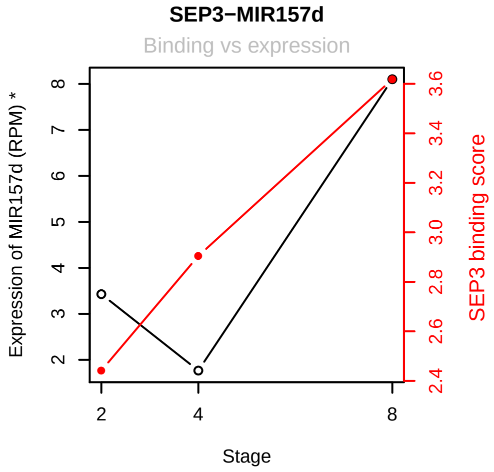

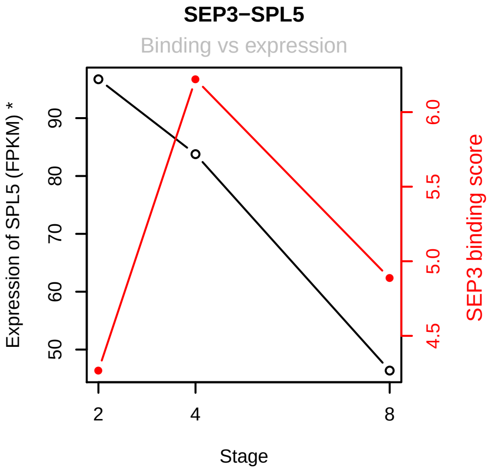

FFL: 63

SEP3−MIR156a−SPL15

Coherent

Evidence: BS

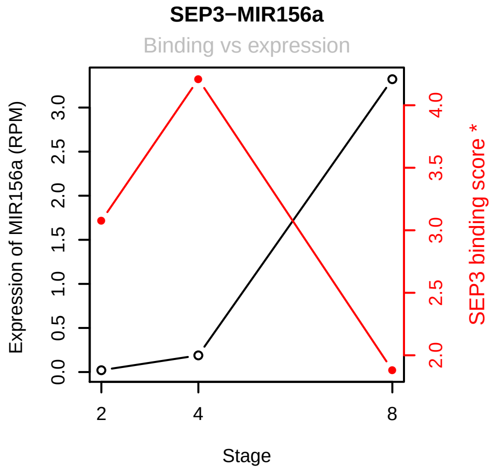

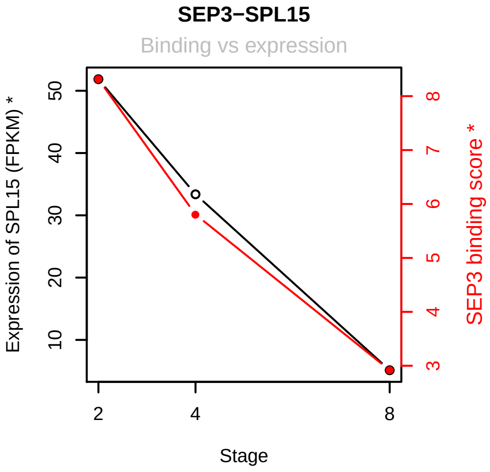

FFL: 64

SEP3−MIR156b−SPL15

Incoherent

Evidence: BS

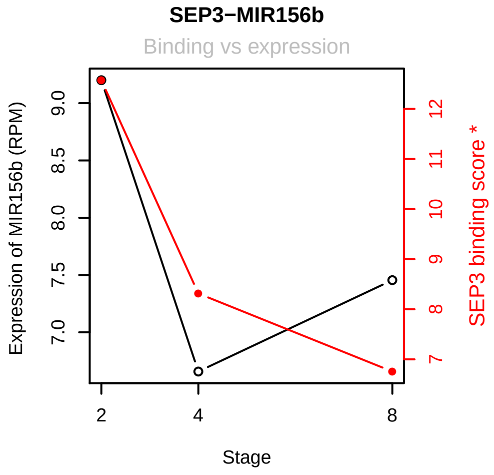

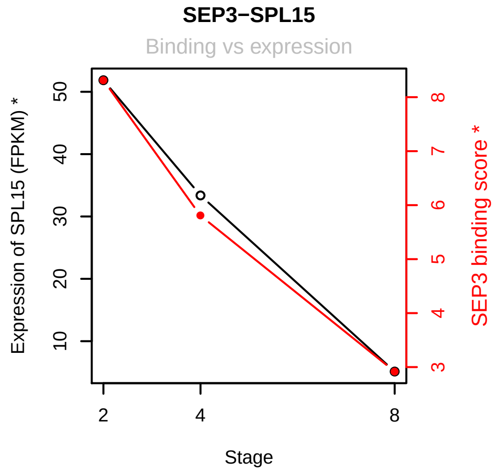

FFL: 65

SEP3−MIR156c−SPL15

Coherent

Evidence: BS

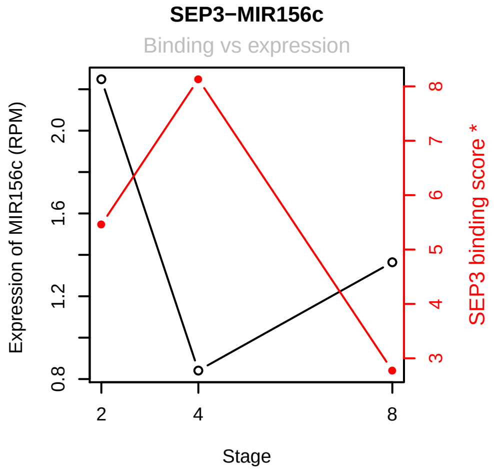

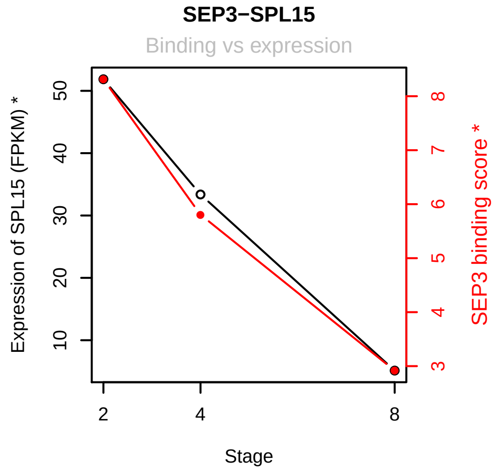

FFL: 66

SEP3−MIR156d−SPL15

Incoherent

Evidence: BS

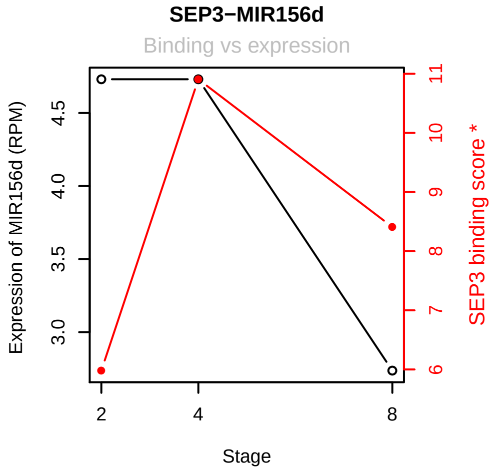

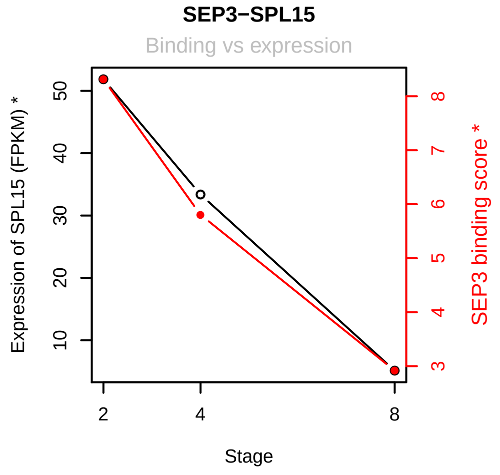

FFL: 67

SEP3−MIR156j−SPL15

Coherent

Evidence: BS

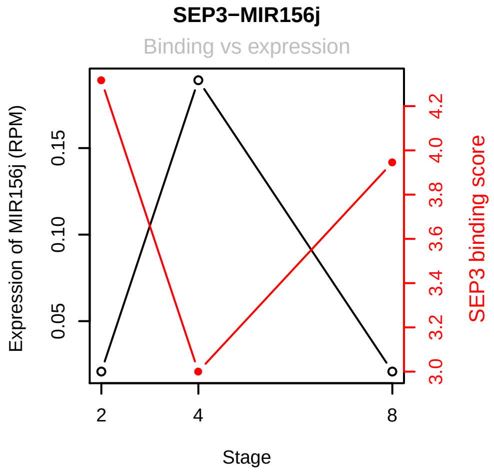

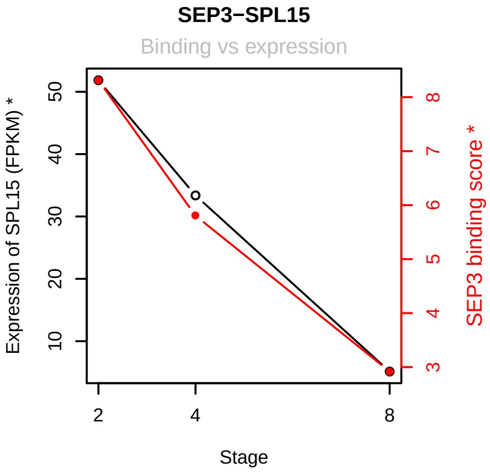

FFL: 68

SEP3−MIR157c−SPL15

Incoherent

Evidence: BS

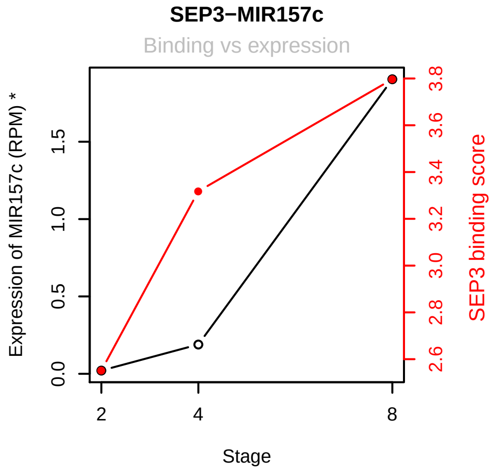

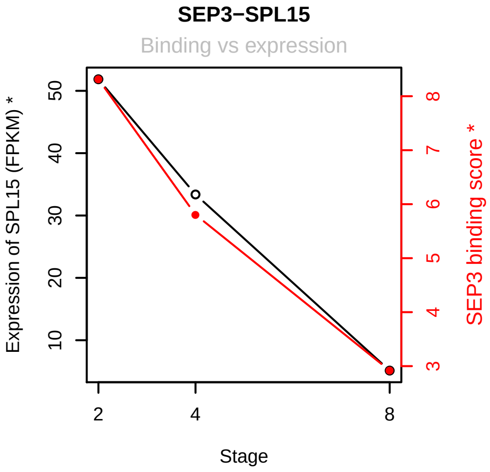

FFL: 69

SEP3−MIR157d−SPL15

Incoherent

Evidence: MIR+BS

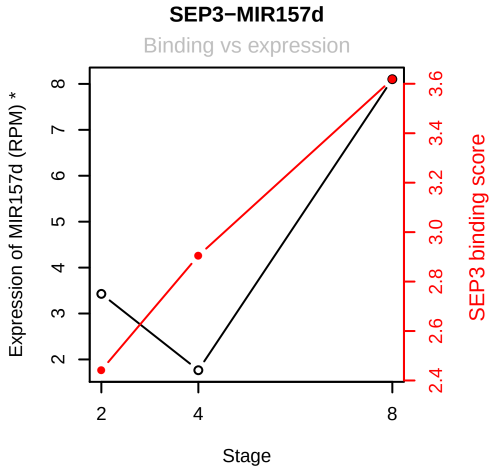

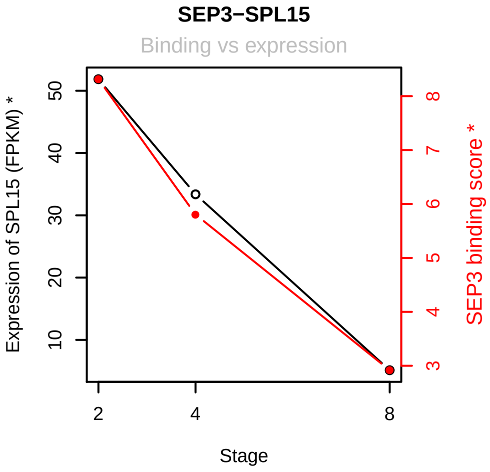

FFL: 70

SEP3−MIR156a−SPL2

Incoherent

Evidence:

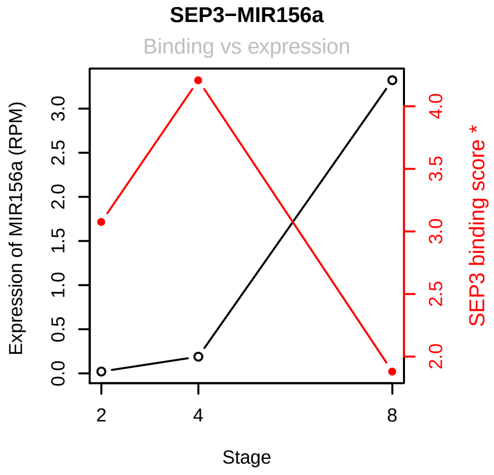

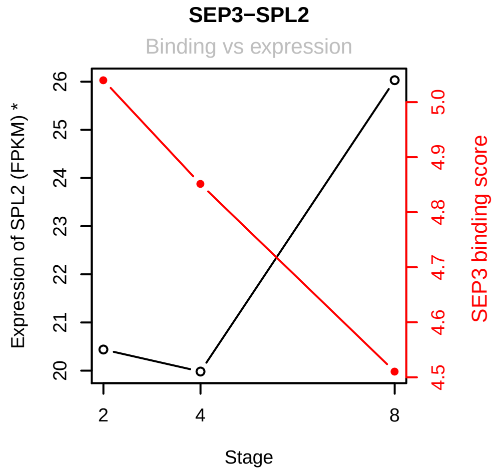

FFL: 71

SEP3−MIR156b−SPL2

Coherent

Evidence:

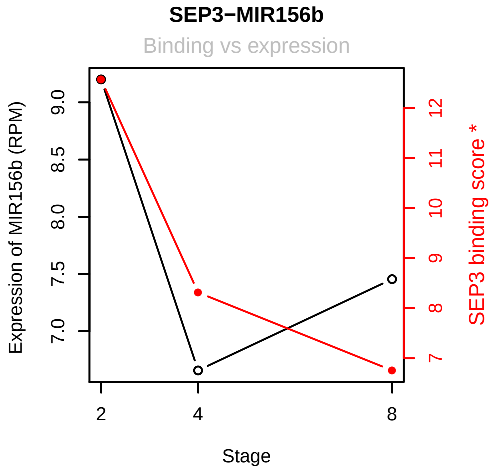

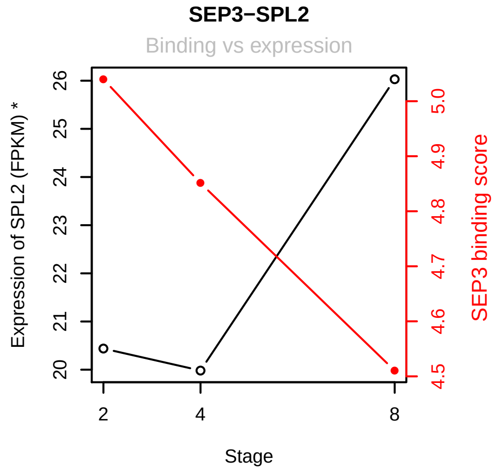

FFL: 72

SEP3−MIR156c−SPL2

Incoherent

Evidence:

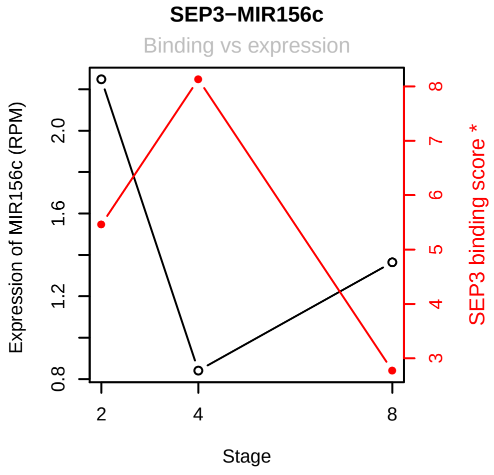

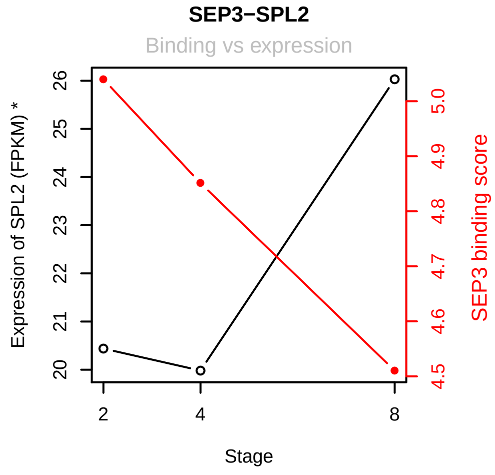

FFL: 73

SEP3−MIR156d−SPL2

Coherent

Evidence:

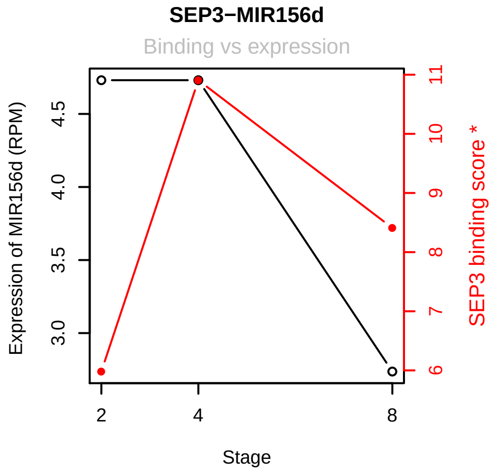

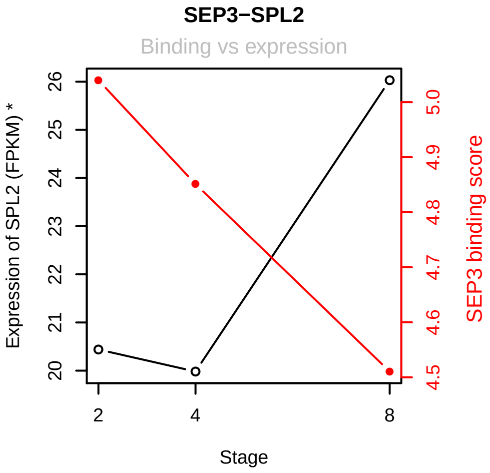

FFL: 74

SEP3−MIR156j−SPL2

Incoherent

Evidence:

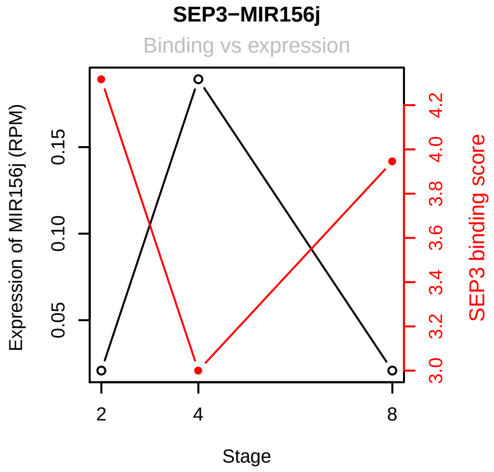

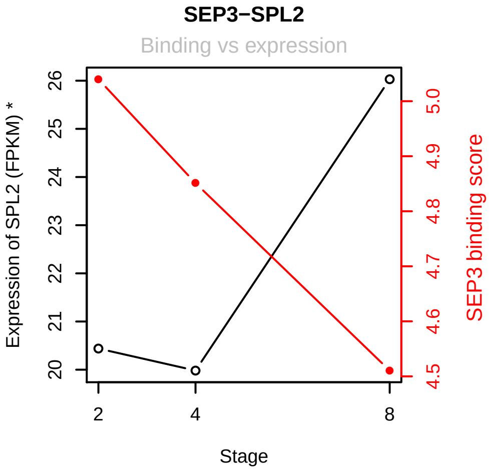

FFL: 75

SEP3−MIR157c−SPL2

Coherent

Evidence:

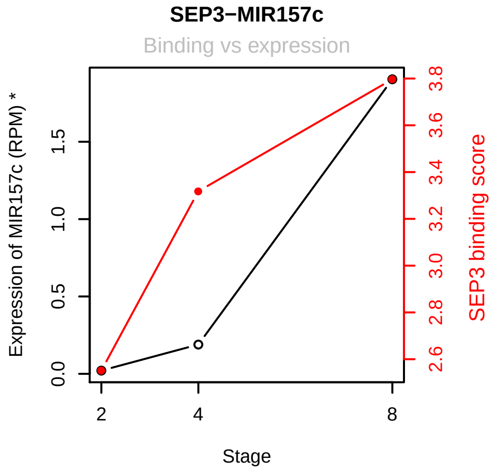

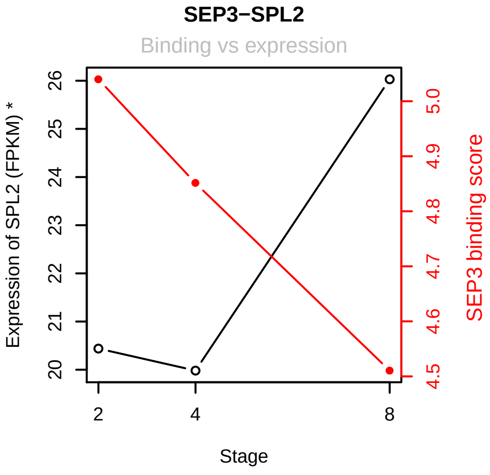

FFL: 76

SEP3−MIR157d−SPL2

Coherent

Evidence: MIR

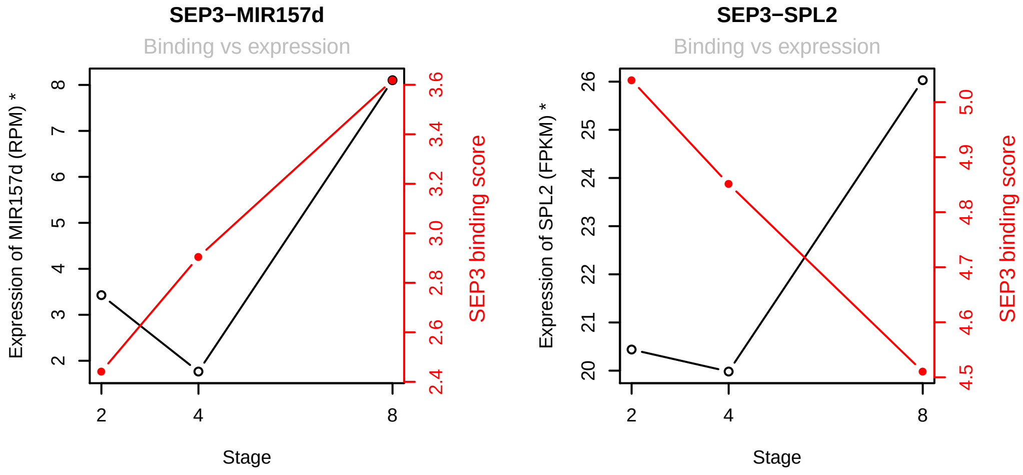

FFL: 77

SEP3−MIR156a−SPL13A

Coherent

Evidence: BS

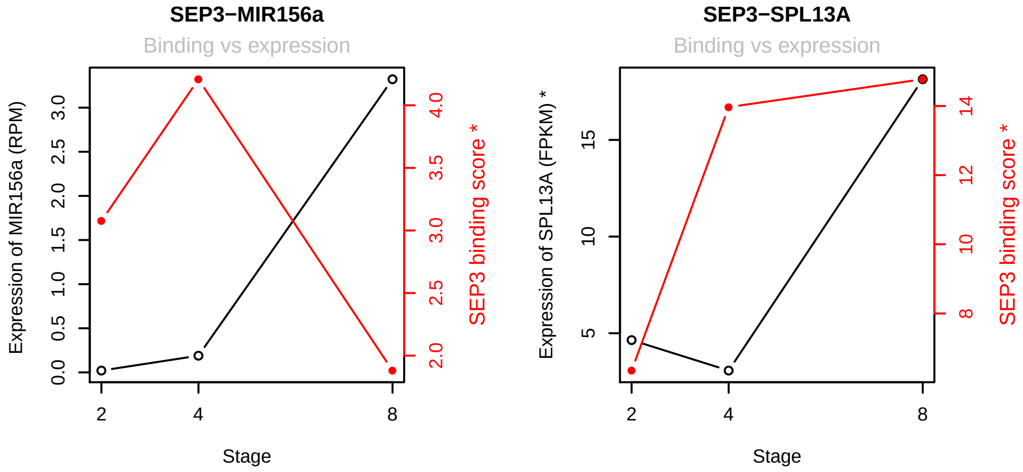

FFL: 78

SEP3−MIR156b−SPL13A

Incoherent

Evidence: BS

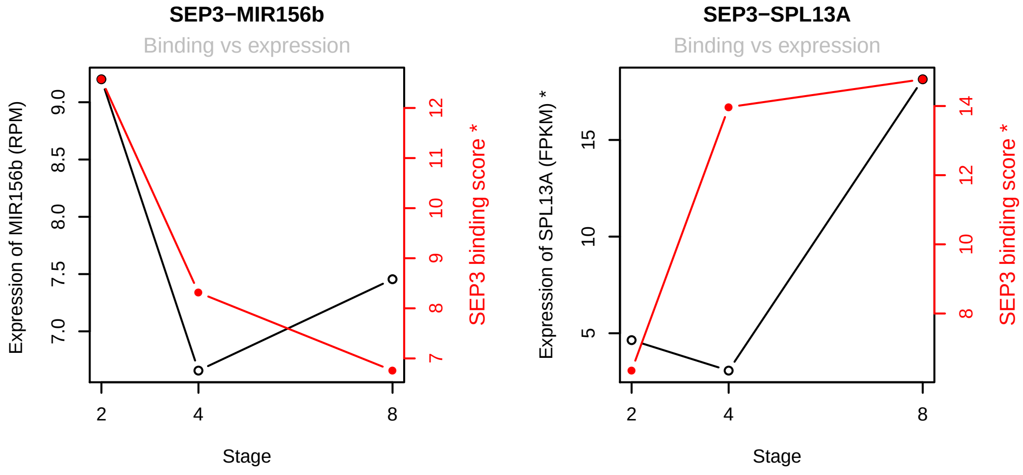

FFL: 79

SEP3−MIR156c−SPL13A

Coherent

Evidence: BS

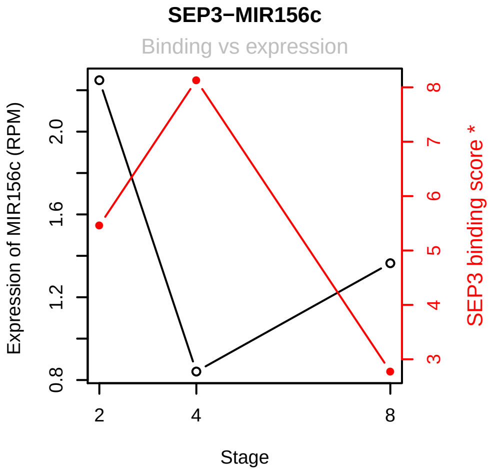

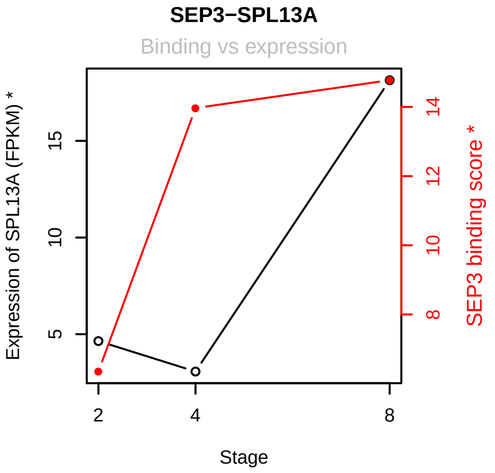

FFL: 80

SEP3−MIR156d−SPL13A

Incoherent

Evidence: BS

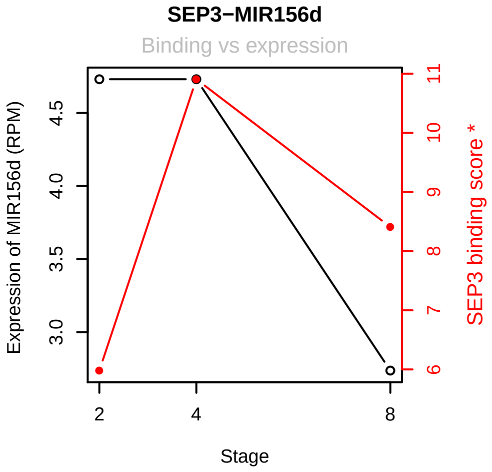

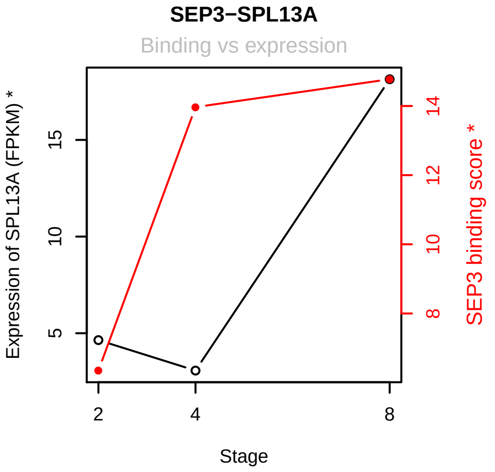

FFL: 81

SEP3−MIR156j−SPL13A

Coherent

Evidence: BS

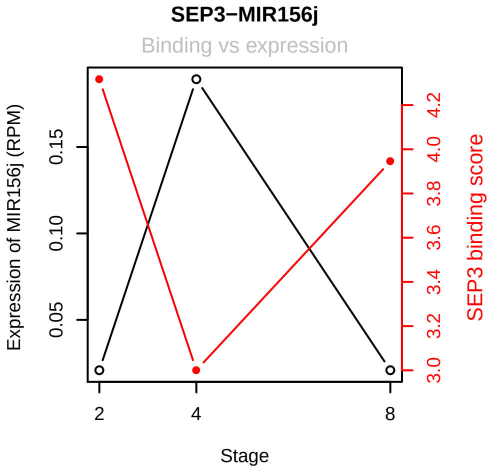

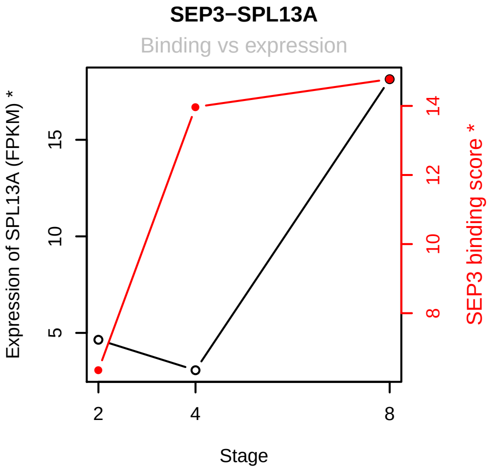

FFL: 82

SEP3−MIR157c−SPL13A

Incoherent

Evidence: BS

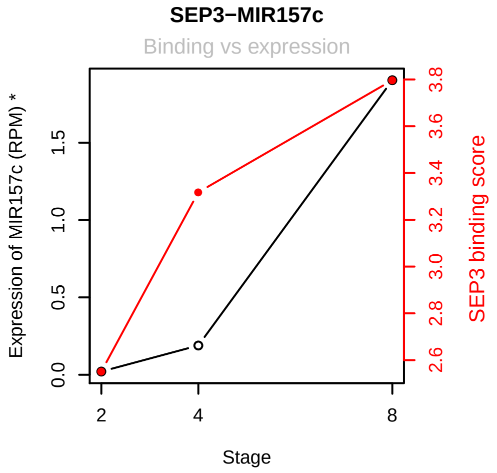

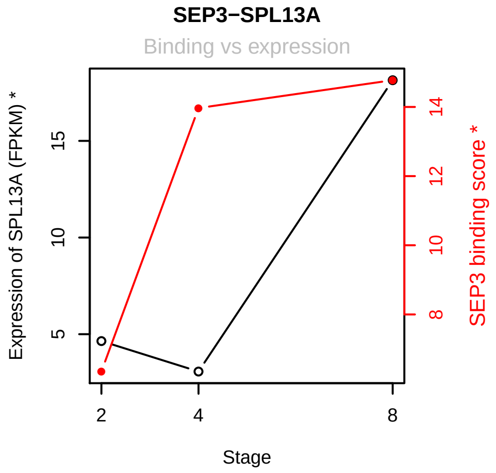

FFL: 83

SEP3−MIR157d−SPL13A

Incoherent

Evidence: MIR+BS

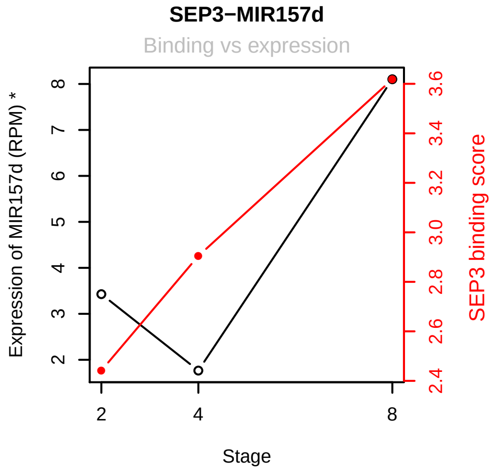

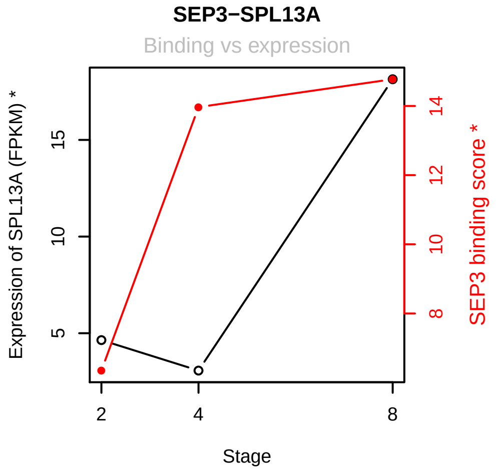

FFL: 84

SEP3−MIR156a−SPL13B

Coherent

Evidence: BS

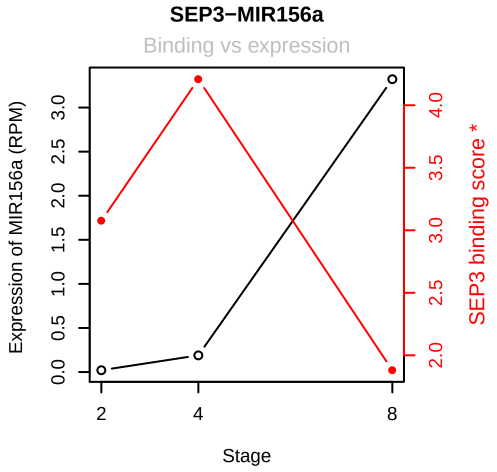

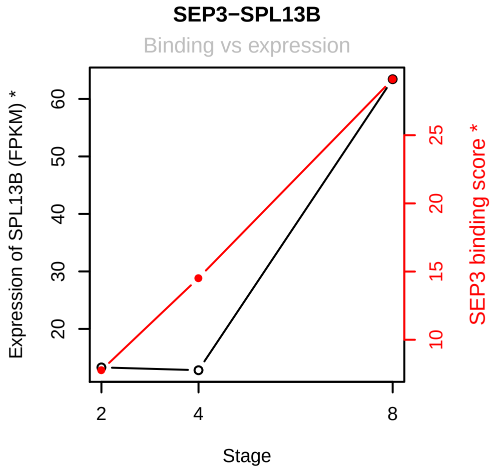

FFL: 85

SEP3−MIR156b−SPL13B

Incoherent

Evidence: BS

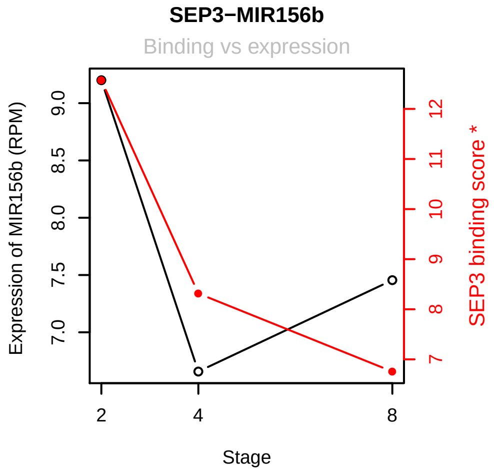

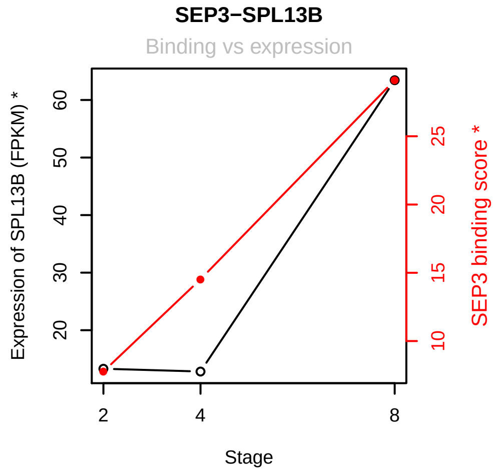

FFL: 86

SEP3−MIR156c−SPL13B

Coherent

Evidence: BS

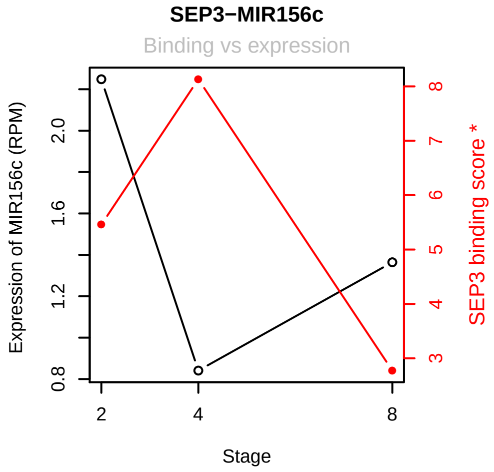

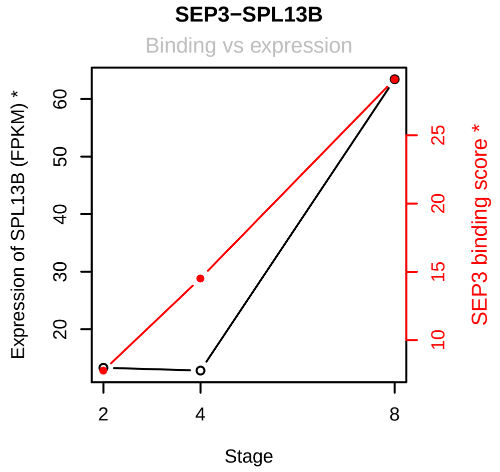

FFL: 87

SEP3−MIR156d−SPL13B

Incoherent

Evidence: BS

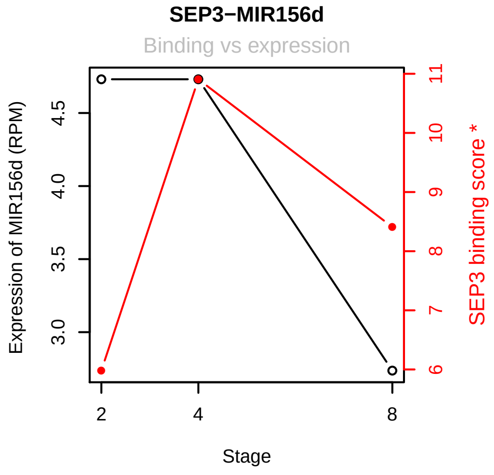

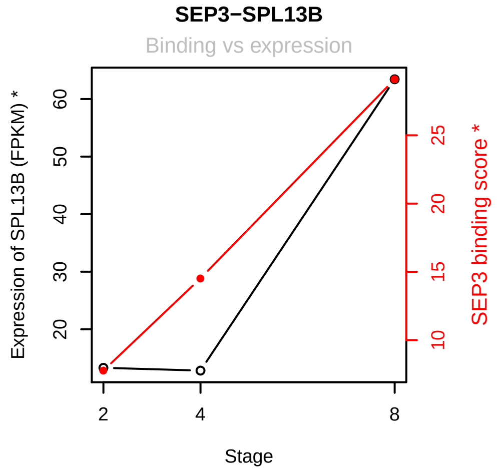

FFL: 88

SEP3−MIR156j−SPL13B

Coherent

Evidence: BS

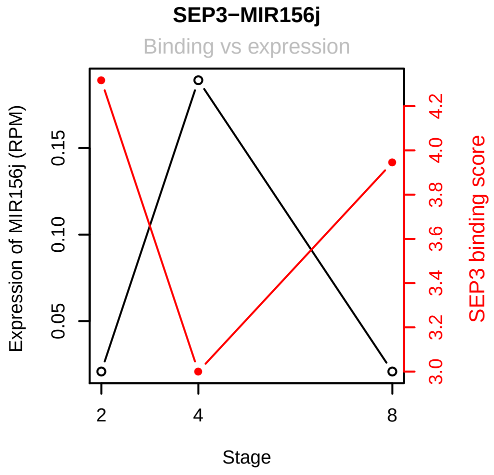

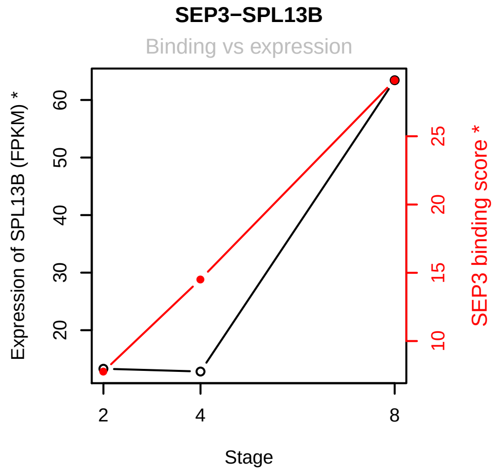

FFL: 89

SEP3−MIR157c−SPL13B

Incoherent

Evidence: BS

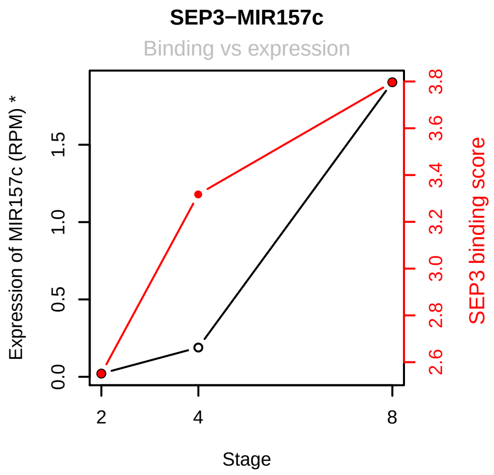

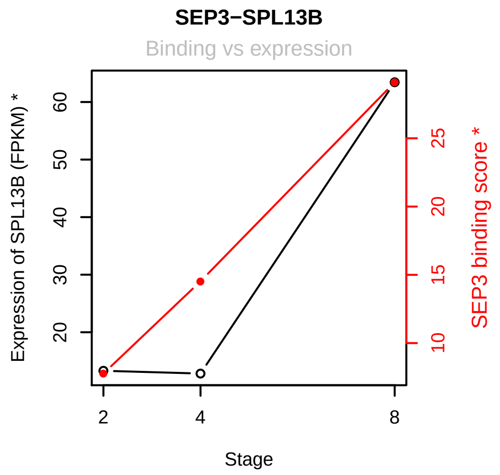

FFL: 90

SEP3−MIR157d−SPL13B

Incoherent

Evidence: MIR+BS

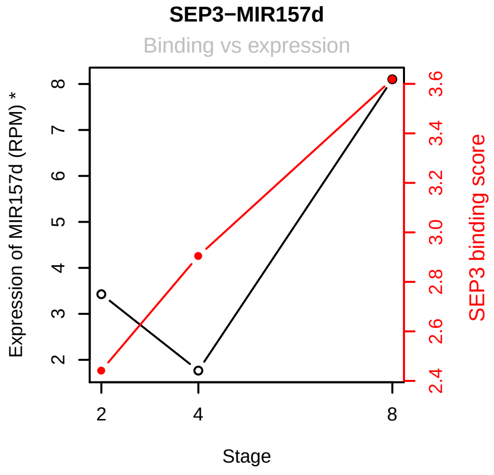

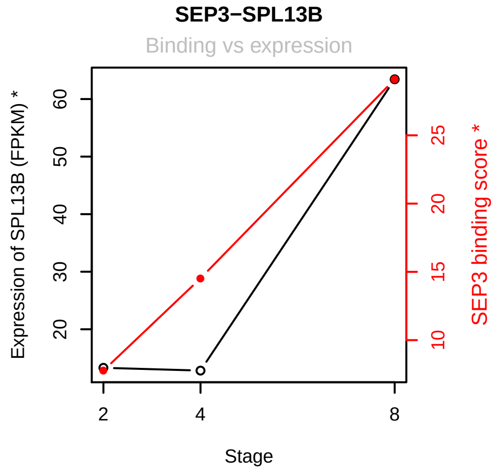

FFL: 91

SEP3−MIR319a−TCP10

Coherent

Evidence: MIR+BS

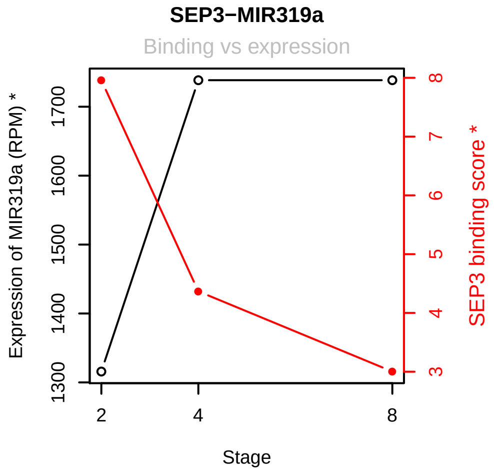

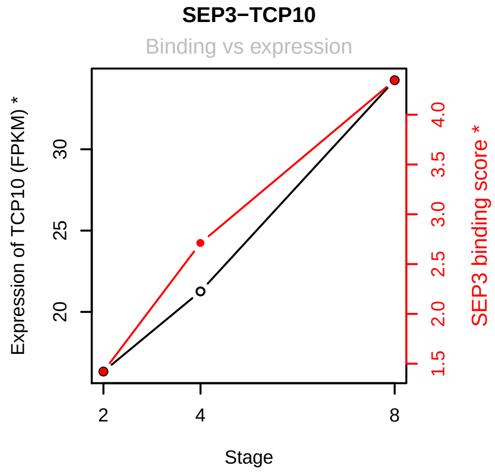

FFL: 92

SEP3−MIR319c−TCP10

Coherent

Evidence: BS

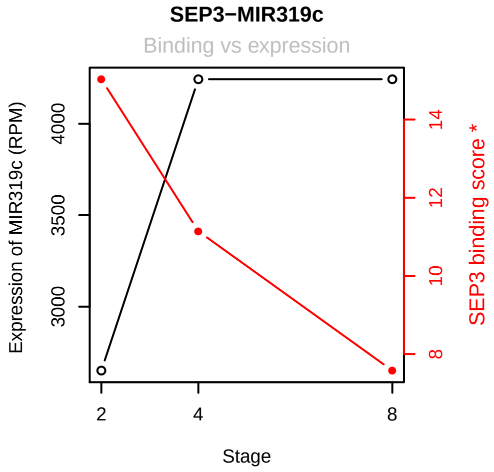

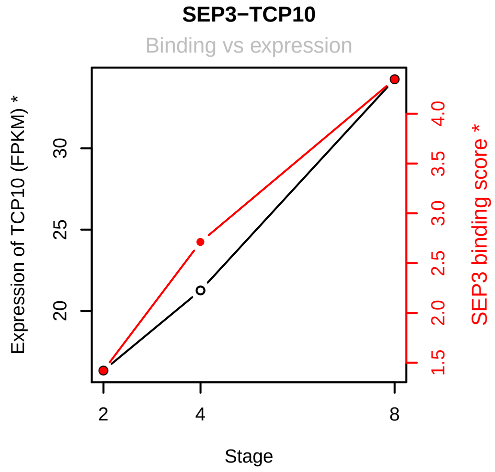

FFL: 93

SEP3−MIR319a−TCP4

Coherent

Evidence: MIR+BS

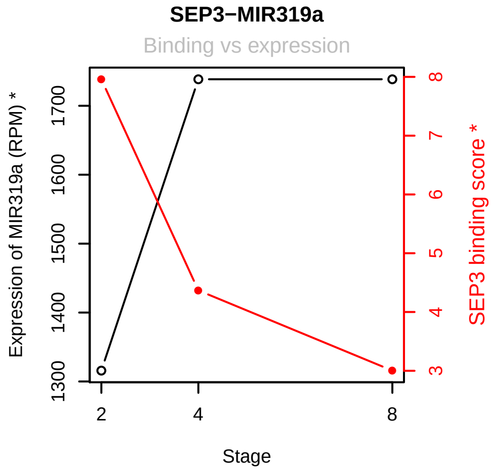

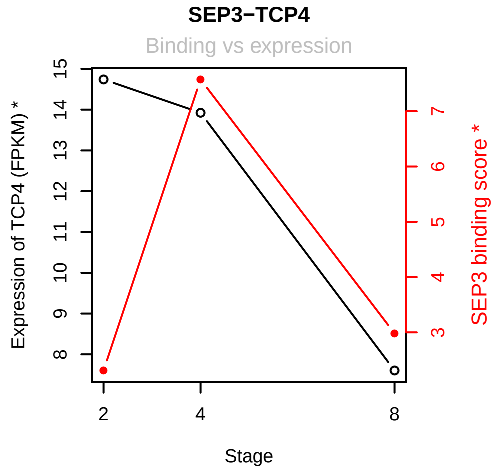

FFL: 94

SEP3−MIR319c−TCP4

Coherent

Evidence: BS

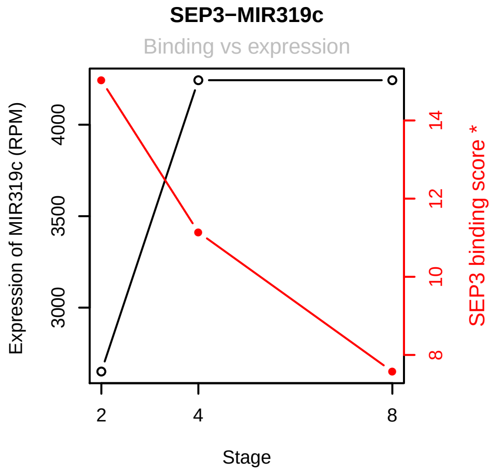

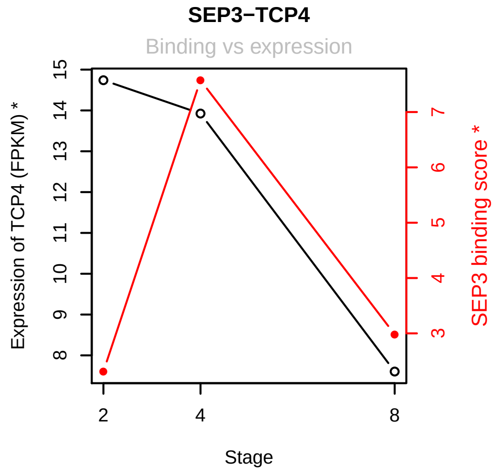

FFL: 95

SEP3−MIR169a−TIFY7

Incoherent

Evidence: BS

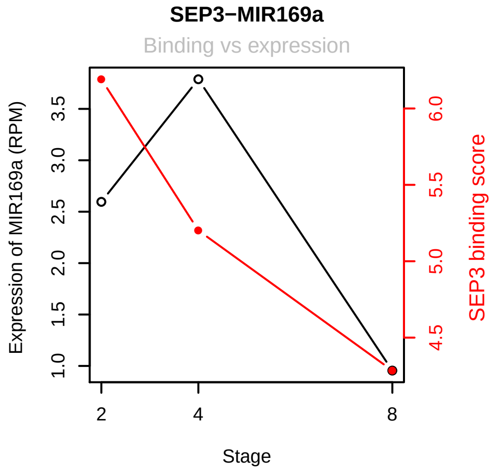

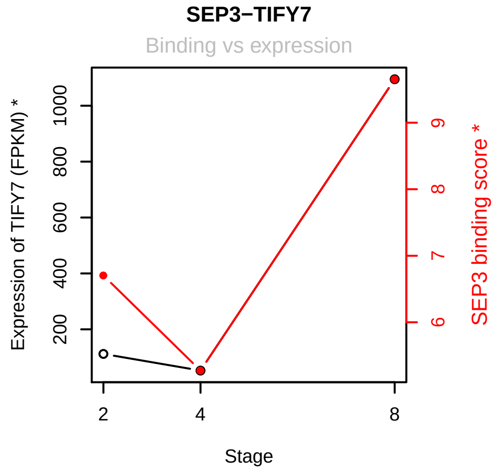

FFL: 96

SEP3−MIR158a−AT4G26120

Incoherent

Evidence:

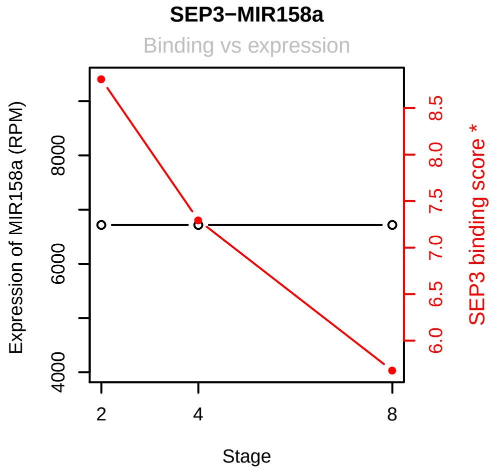

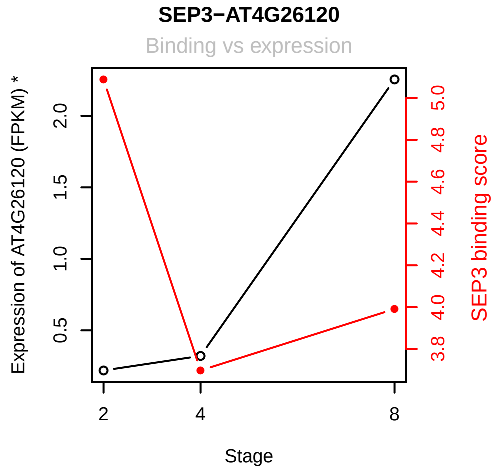

FFL: 97

AP1−MIR172d−TOE1

Coherent

Evidence: MIR

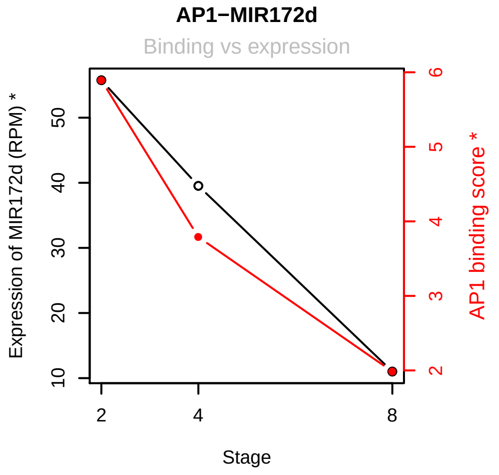

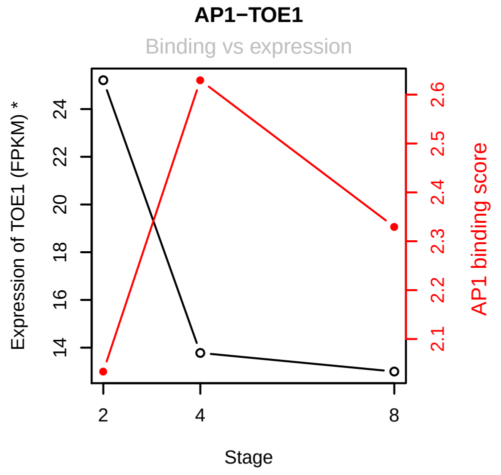

FFL: 98

AP1−MIR172d−SNZ

Coherent

Evidence: MIR+BS

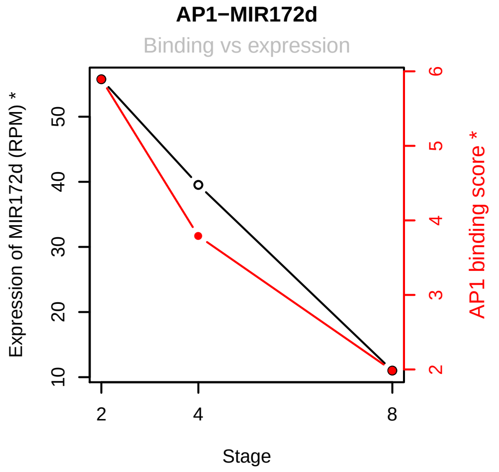

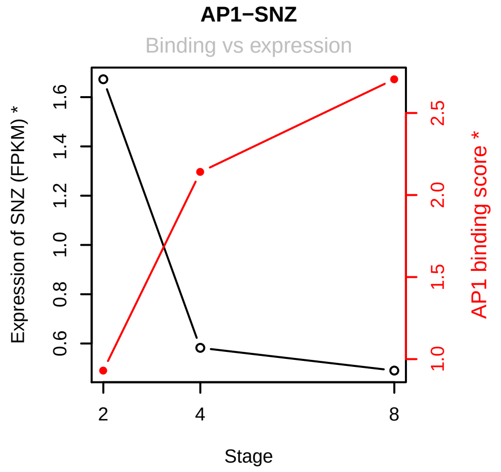

FFL: 99

AP1−MIR172d−AP2

Coherent

Evidence: MIR

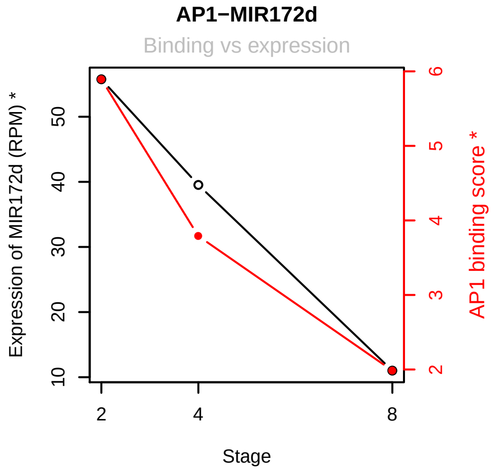

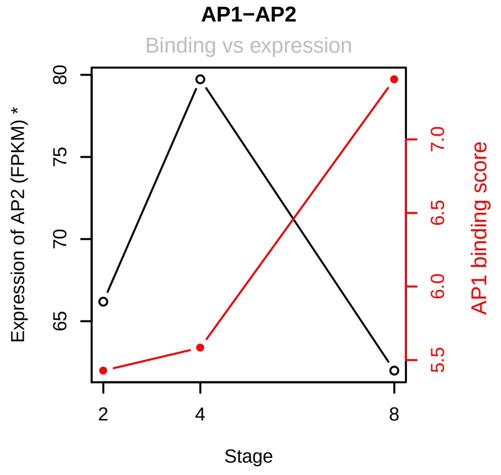

FFL: 100

AP1−MIR172d−TOE3

Incoherent

Evidence: MIR

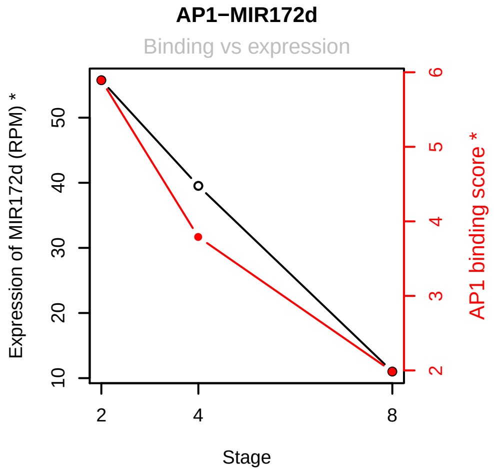

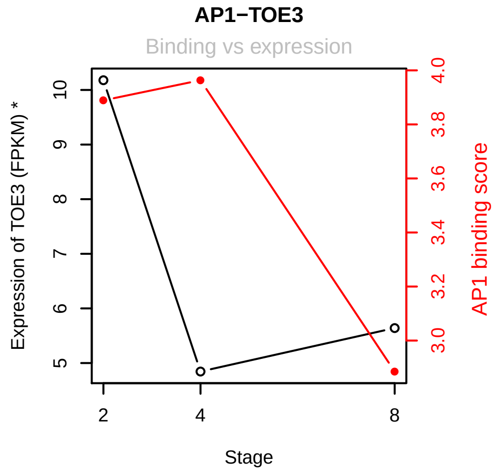

FFL: 101

AP1−MIR160a−ARF10

Coherent

Evidence: BS

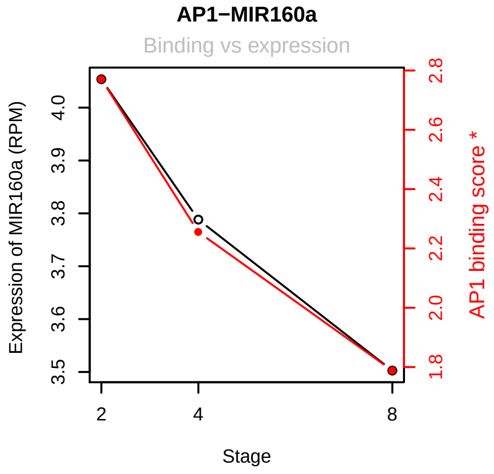

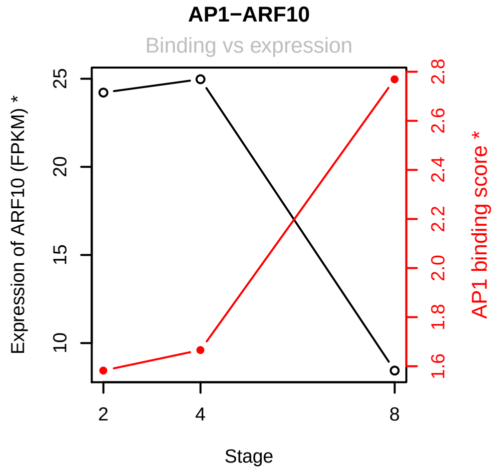

FFL: 102

AP1−MIR160c−ARF10

Incoherent

Evidence: BS

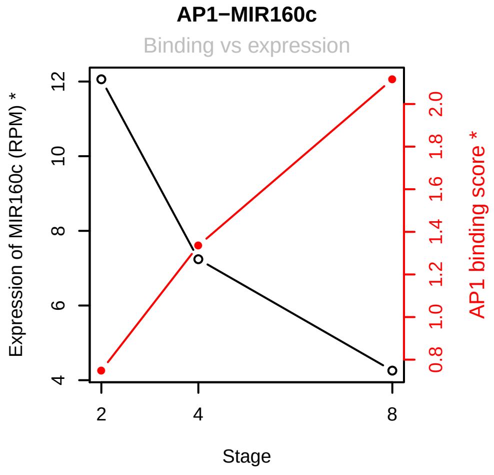

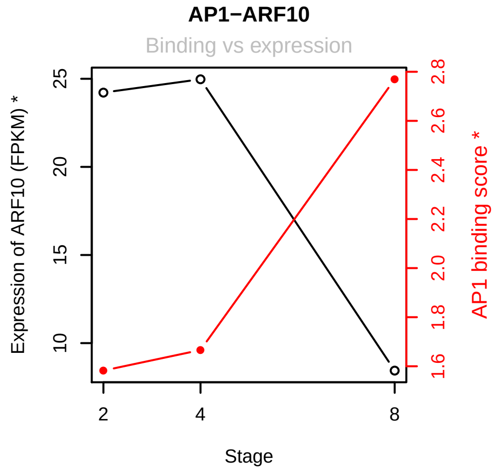

FFL: 103

AP1−MIR847−COL1

Coherent

Evidence: BS

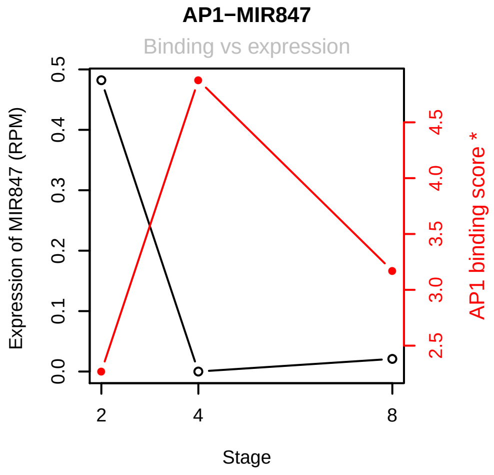

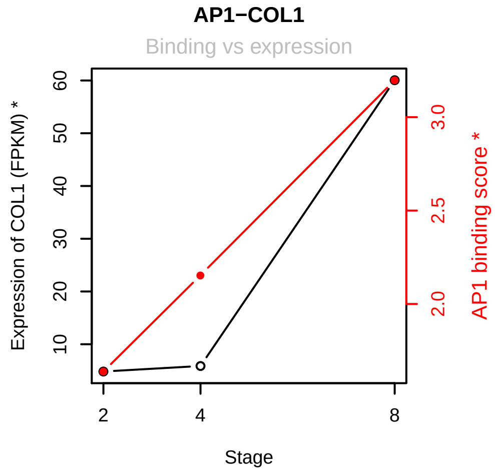

FFL: 104

AP1−MIR169i−NF−YA10

Incoherent

Evidence: BS

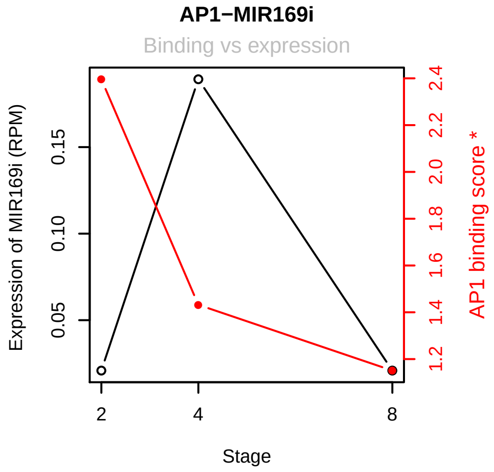

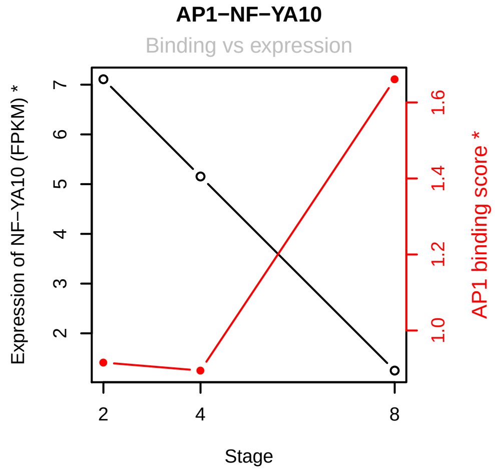

FFL: 105

AP1−MIR165b−PHB−1D

Incoherent

Evidence: BS

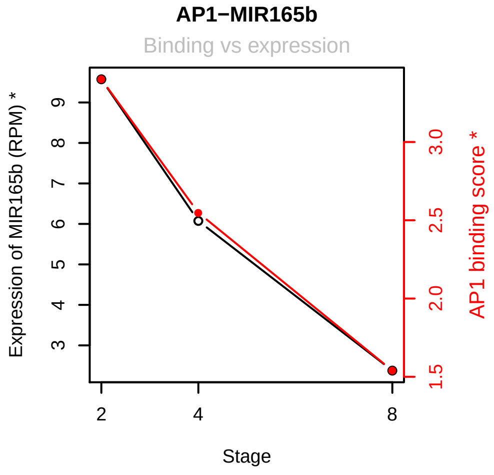

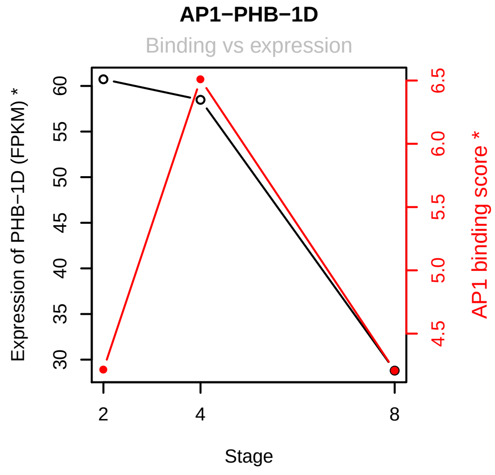

FFL: 106

AP1−MIR166b−PHB−1D

Coherent

Evidence: BS

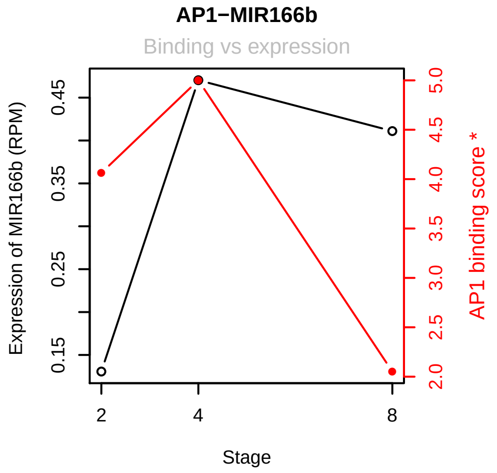

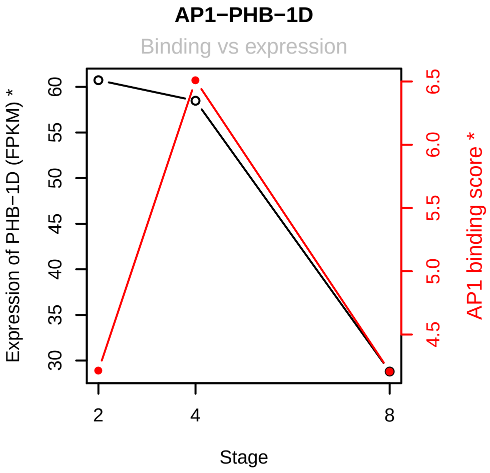

FFL: 107

AP1−MIR166c−PHB−1D

Coherent

Evidence: BS

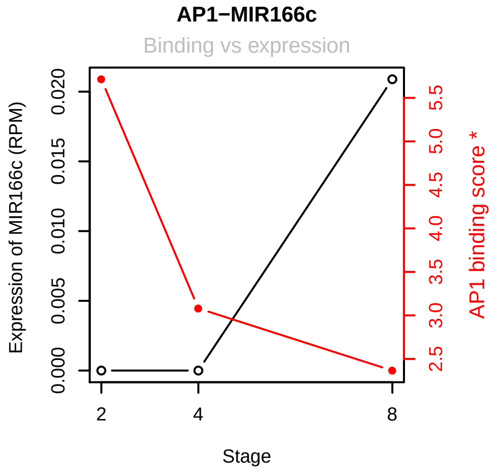

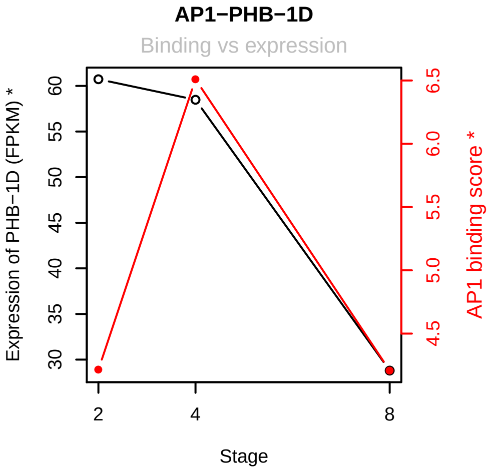

FFL: 108

AP1−MIR159b−SHP1

Coherent

Evidence: BS

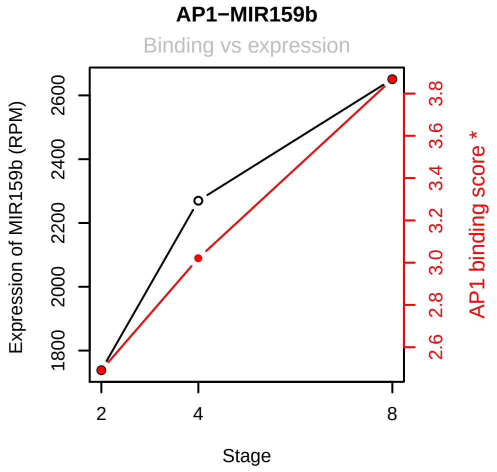

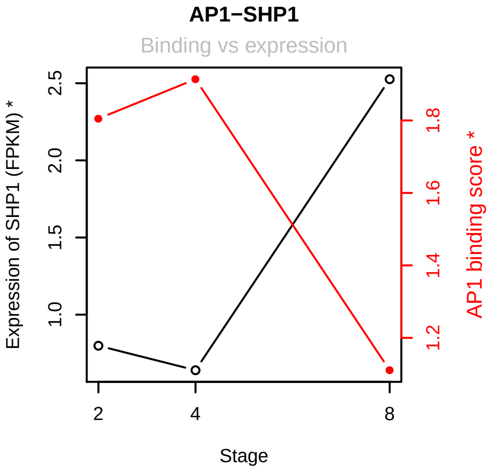

FFL: 109

AP1−MIR156c−SPL3

Incoherent

Evidence:

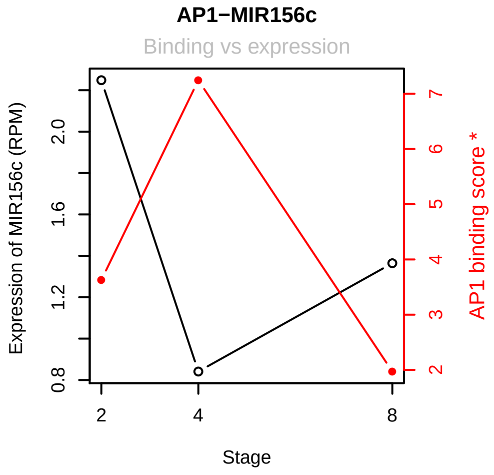

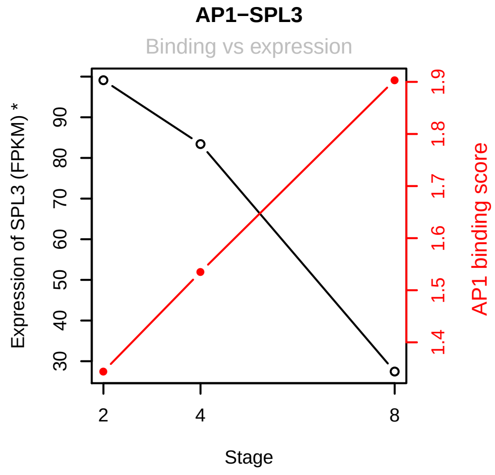

FFL: 110

AP1−MIR156d−SPL3

Incoherent

Evidence:

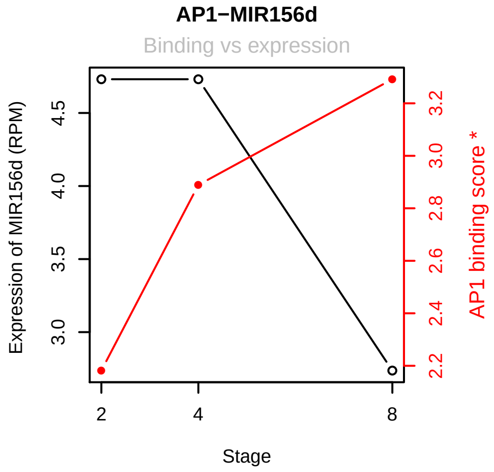

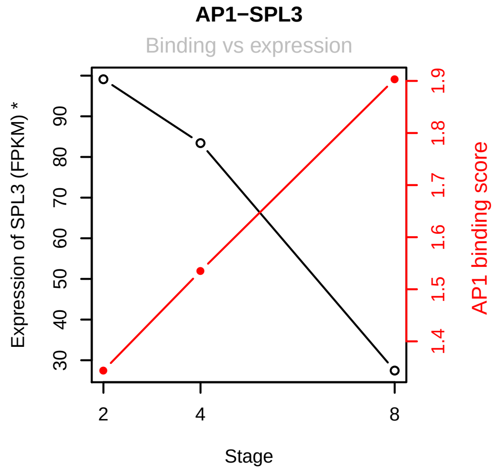

FFL: 111

AP1−MIR156j−SPL3

Incoherent

Evidence:

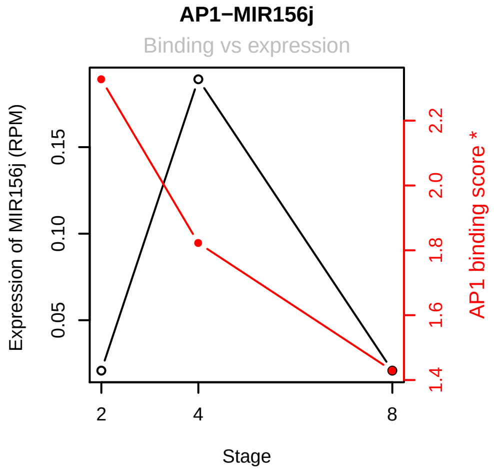

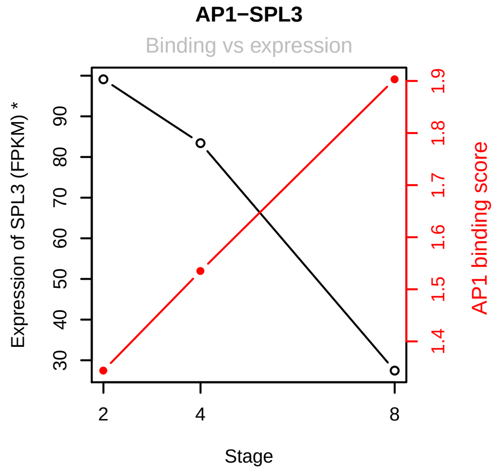

FFL: 112

AP1−MIR156c−SPL9

Coherent

Evidence: BS

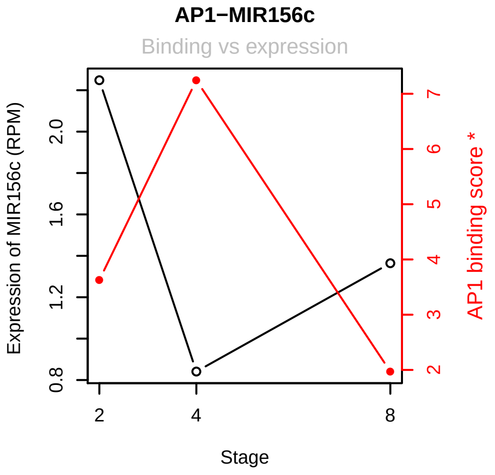

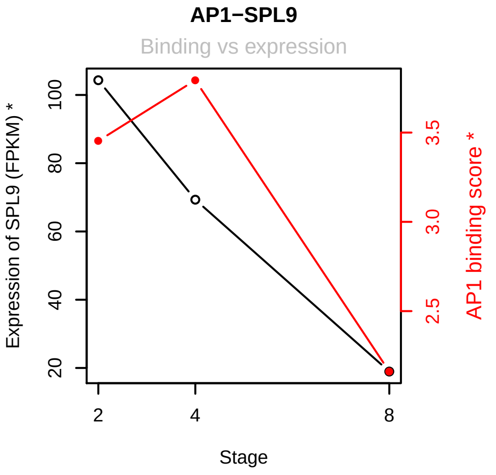

FFL: 113

AP1−MIR156d−SPL9

Coherent

Evidence: BS

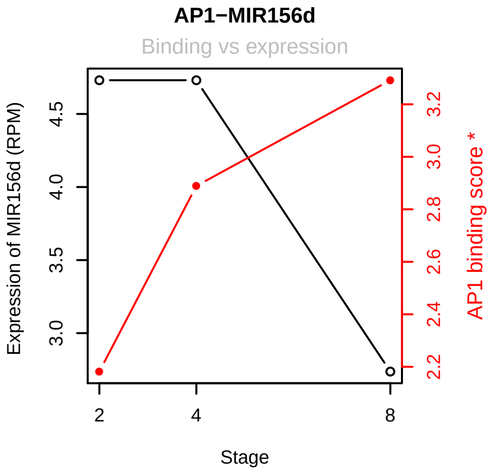

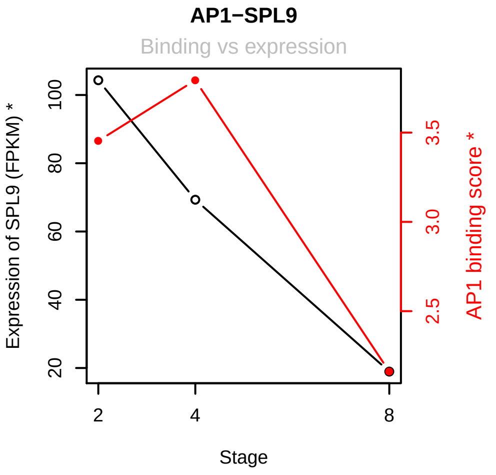

FFL: 114

AP1−MIR156j−SPL9

Coherent

Evidence: BS

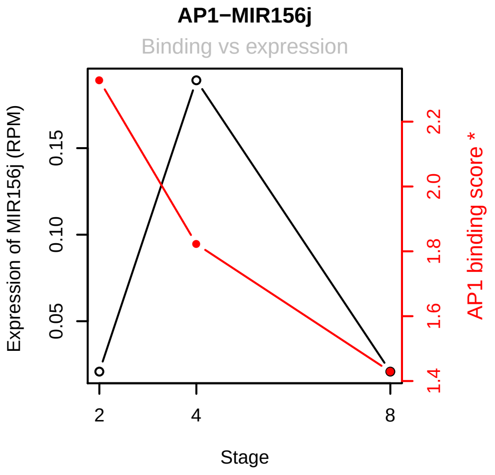

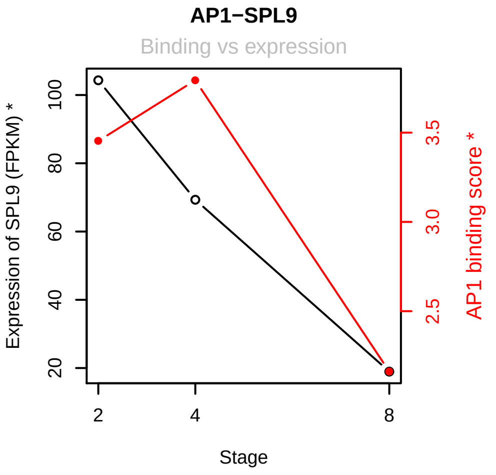

FFL: 115

AP1−MIR157c−SPL9

Coherent

Evidence: BS

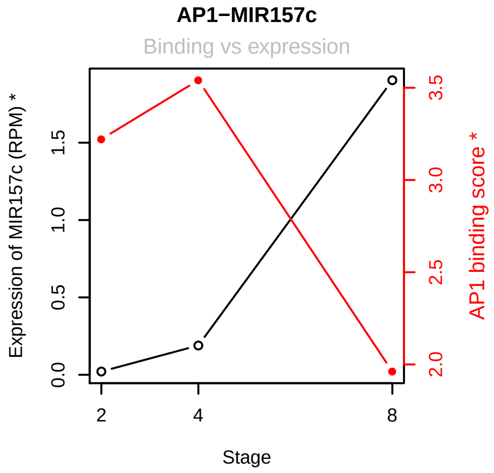

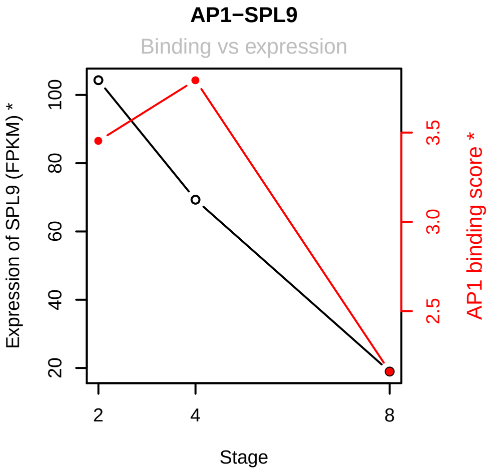

FFL: 116

AP1−MIR157d−SPL9

Coherent

Evidence: MIR+BS

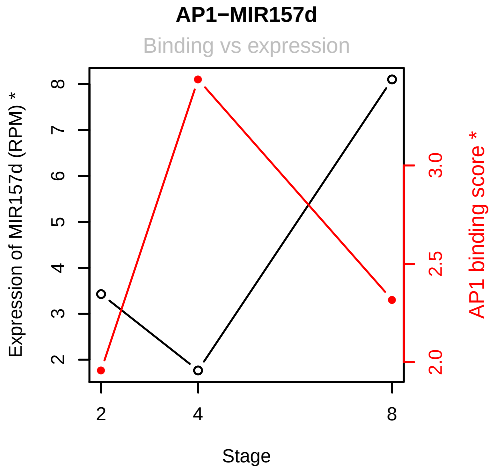

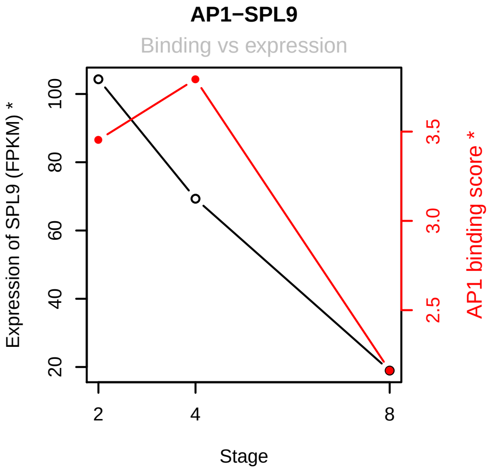

FFL: 117

AP1−MIR156c−SPL5

Coherent

Evidence: BS

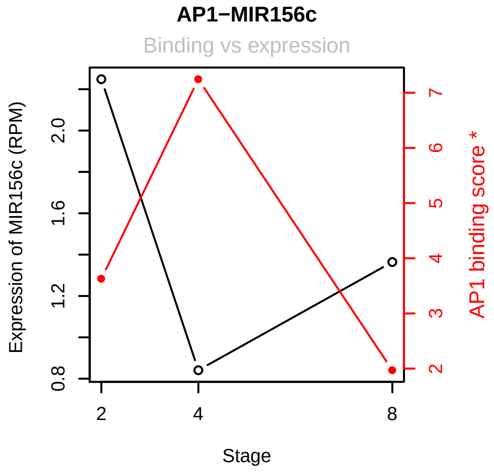

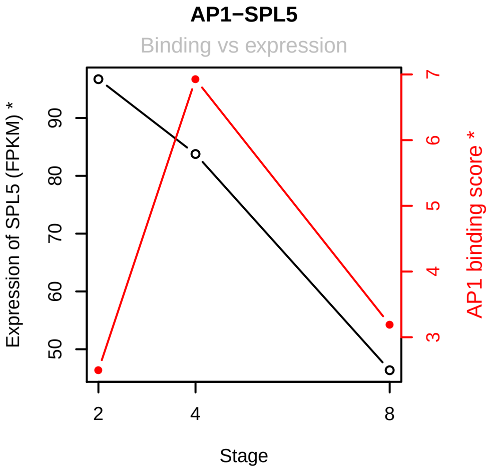

FFL: 118

AP1−MIR156d−SPL5

Coherent

Evidence: BS

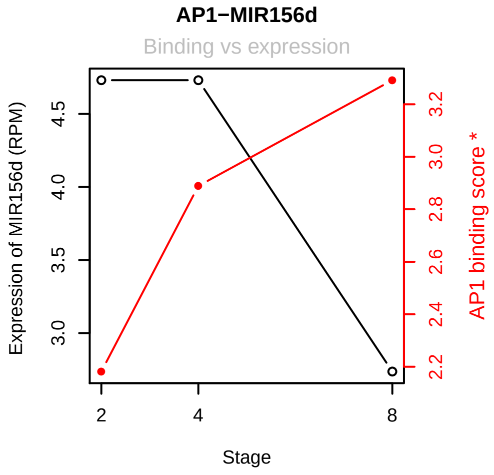

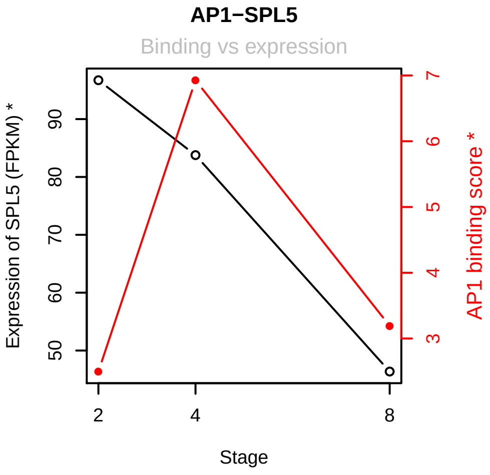

FFL: 119

AP1−MIR156j−SPL5

Coherent

Evidence: BS

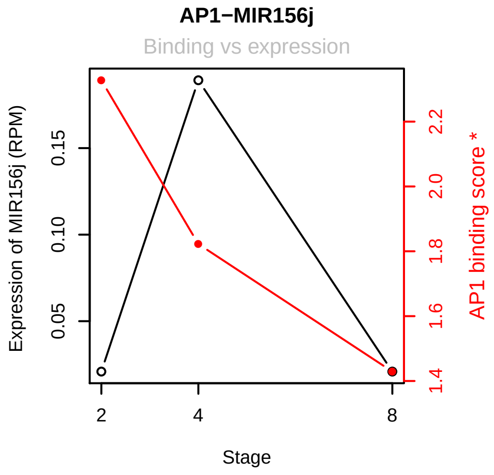

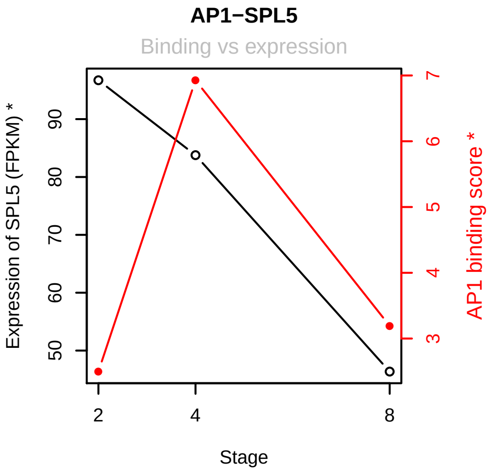

FFL: 120

AP1−MIR157d−SPL5

Coherent

Evidence: MIR+BS

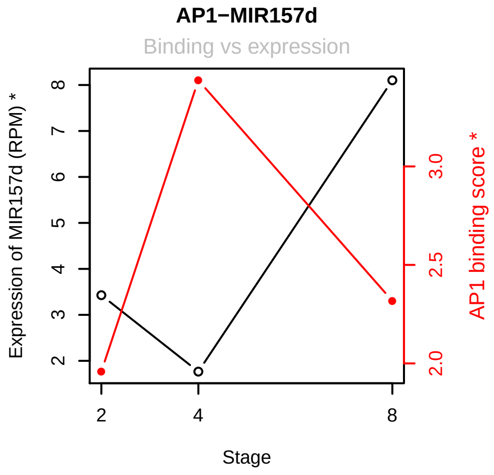

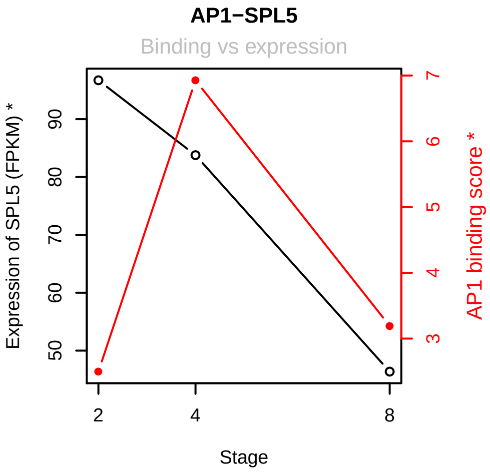

FFL: 121

AP1−MIR156c−SPL15

Coherent

Evidence: BS

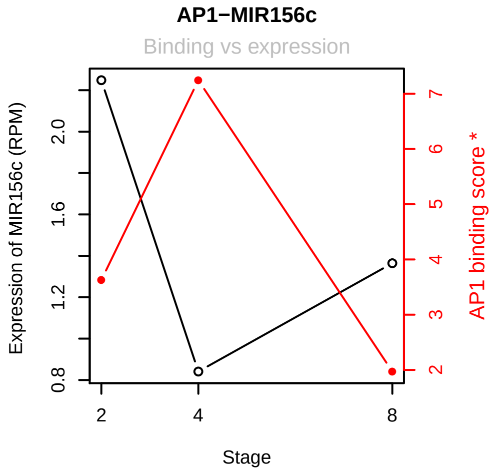

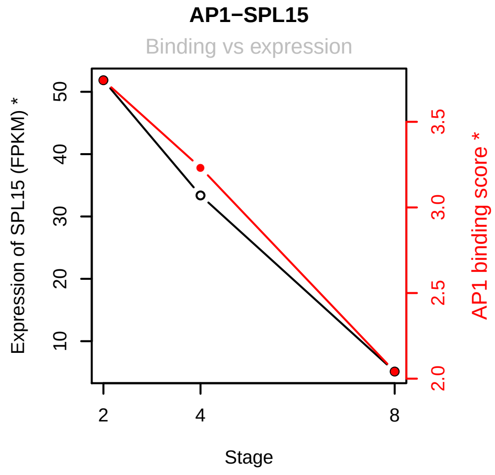

FFL: 122

AP1−MIR156d−SPL15

Coherent

Evidence: BS

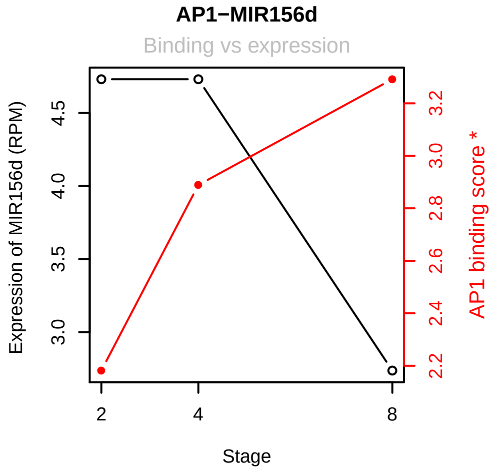

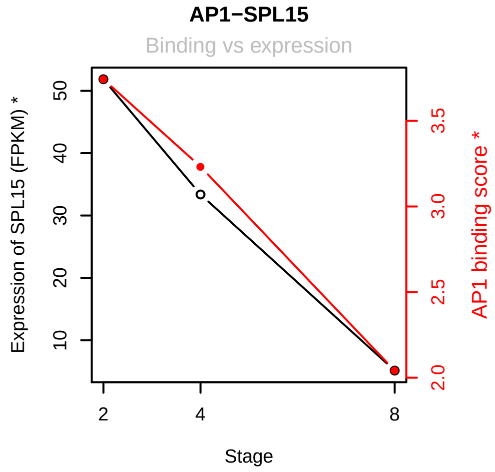

FFL: 123

AP1−MIR156j−SPL15

Coherent

Evidence: BS

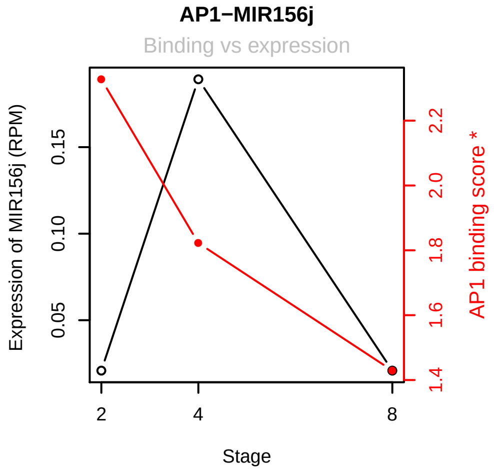

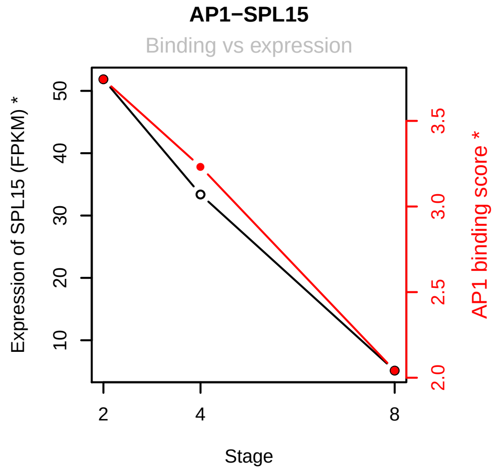

FFL: 124

AP1−MIR157c−SPL15

Coherent

Evidence: BS

AP1−MIR157c

Binding vs expression

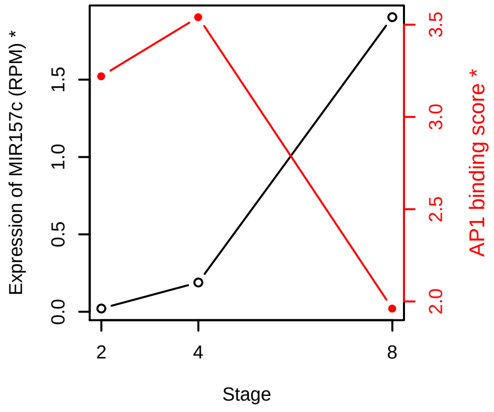

AP1−SPL15

Binding vs expression

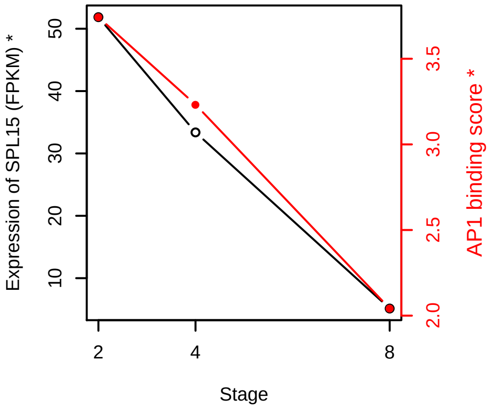

FFL: 125

AP1−MIR157d−SPL15

Coherent

Evidence: MIR+BS

AP1−MIR157d

Binding vs expression

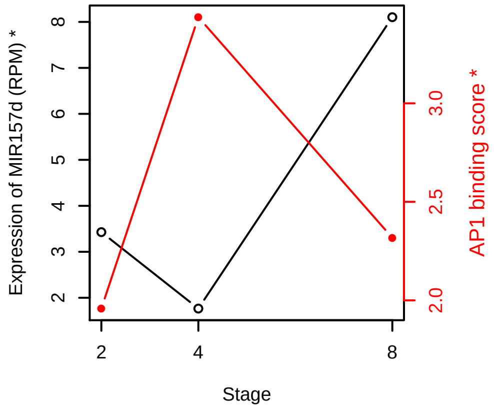

AP1−SPL15

Binding vs expression

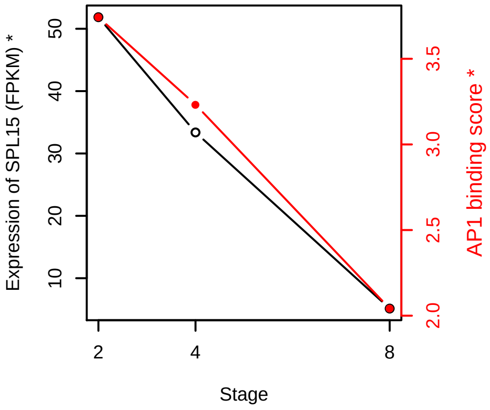

FFL: 126

AP1−MIR156c−SPL2

Coherent

Evidence: BS

AP1−MIR156c

Binding vs expression

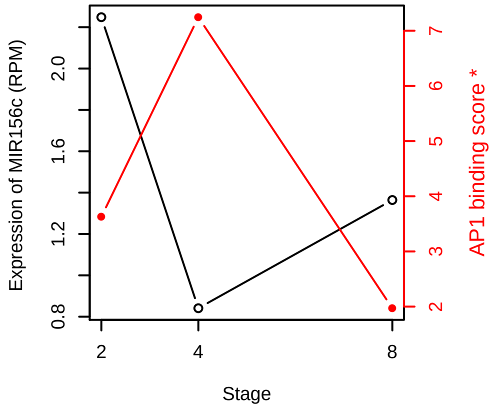

AP1−SPL2

Binding vs expression

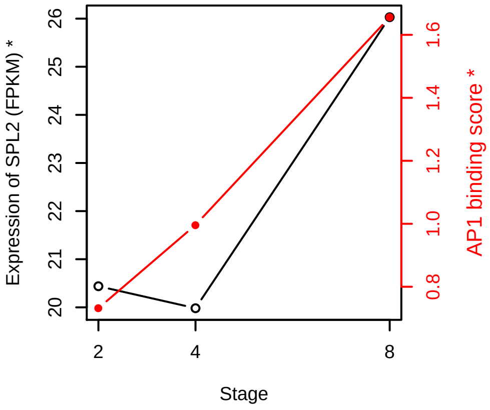

FFL: 127

AP1−MIR156d−SPL2

Coherent

Evidence: BS

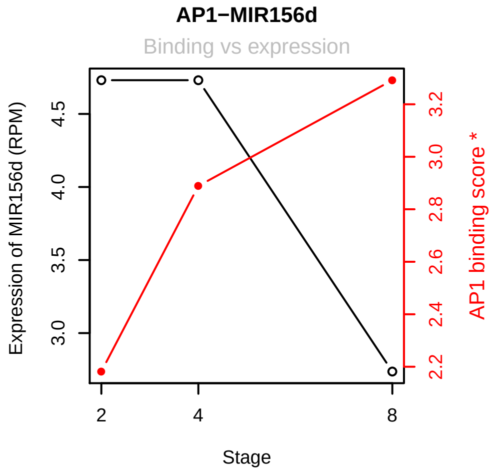

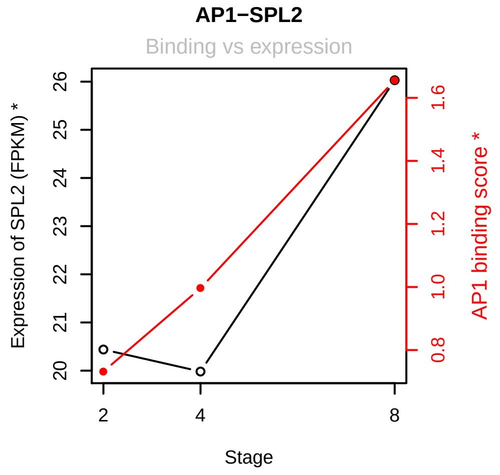

FFL: 128

AP1−MIR156j−SPL2

Coherent

Evidence: BS

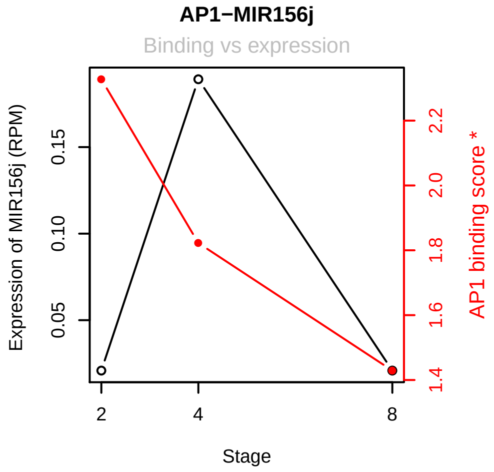

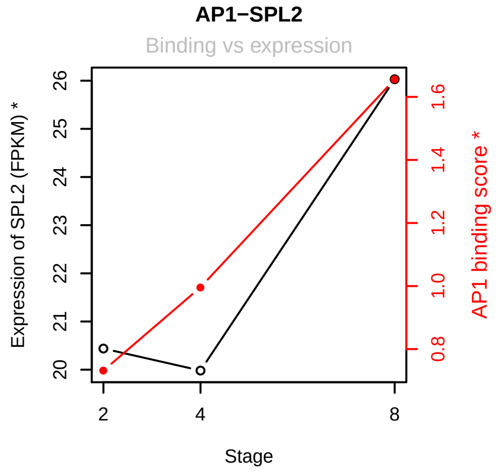

FFL: 129

AP1−MIR157c−SPL2

Coherent

Evidence: BS

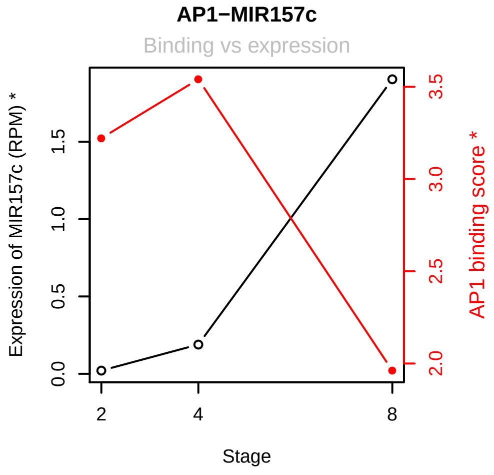

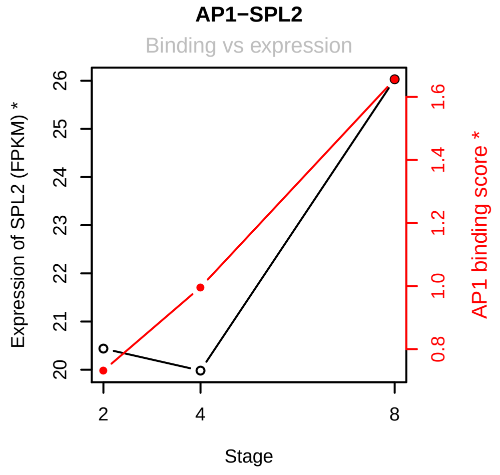

FFL: 130

AP1−MIR157d−SPL2

Coherent

Evidence: MIR+BS

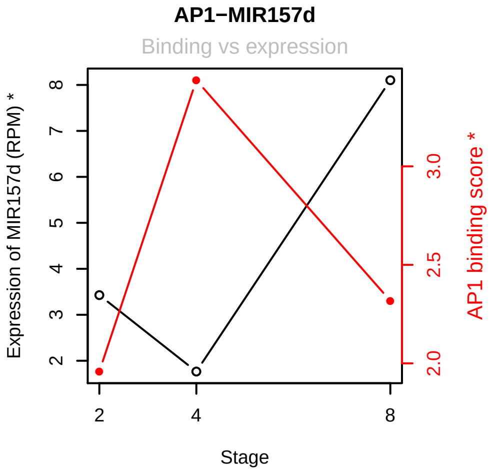

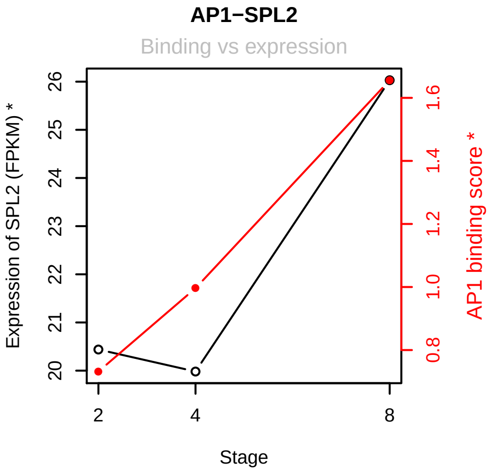

FFL: 131

AP1−MIR319a−TCP10

Coherent

Evidence: MIR+BS

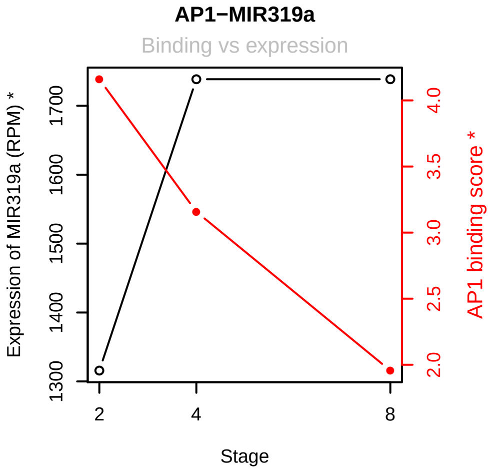

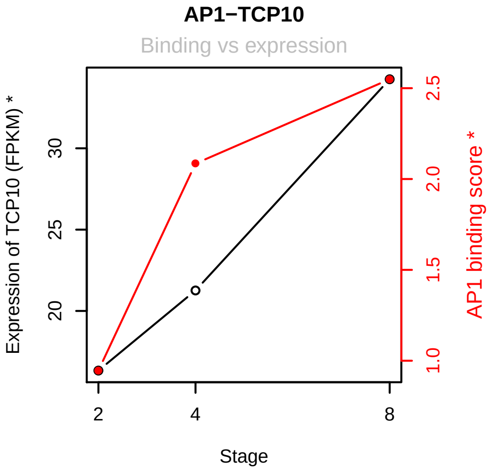

FFL: 132

AP1−MIR319c−TCP10

Incoherent

Evidence: BS

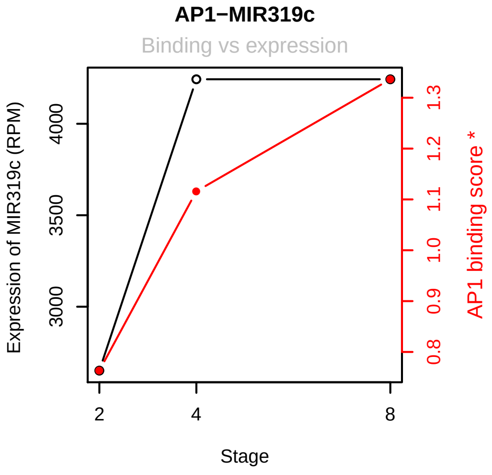

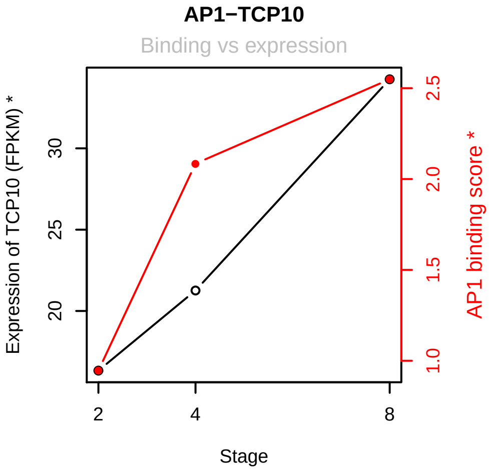

FFL: 133

AP1−MIR319a−TCP4

Coherent

Evidence: MIR+BS

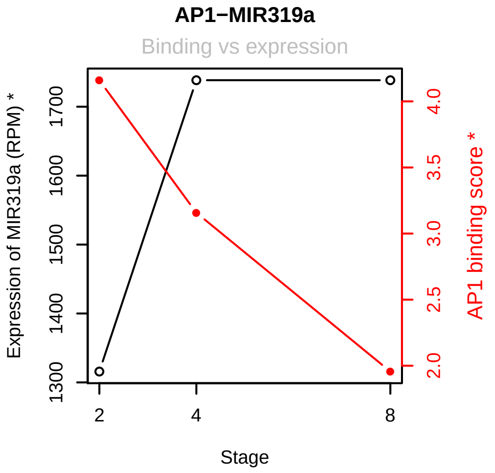

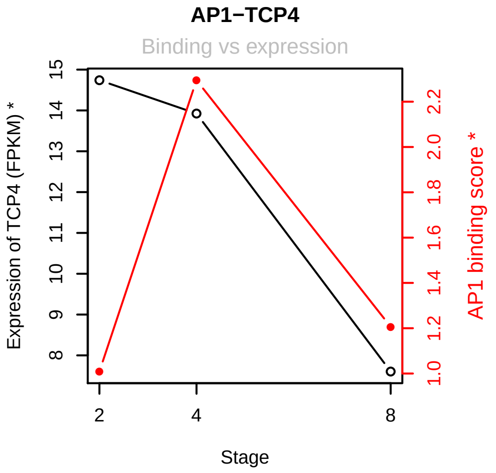

FFL: 134

AP1−MIR319c−TCP4

Incoherent

Evidence: BS

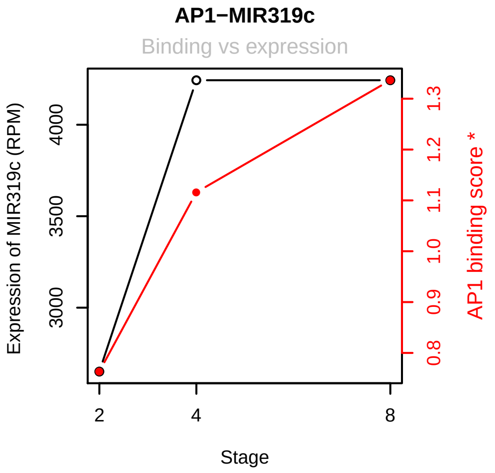

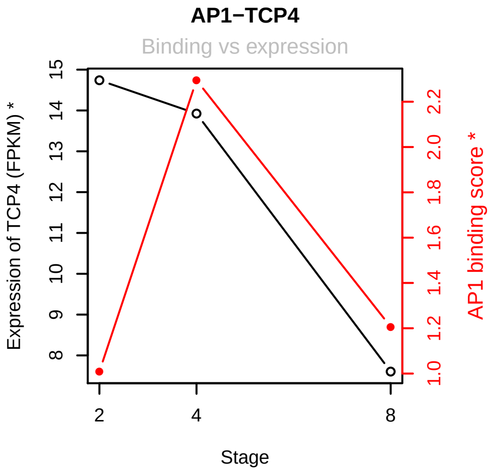

FFL: 135

AP1−MIR169a−TIFY7

Incoherent

Evidence: BS

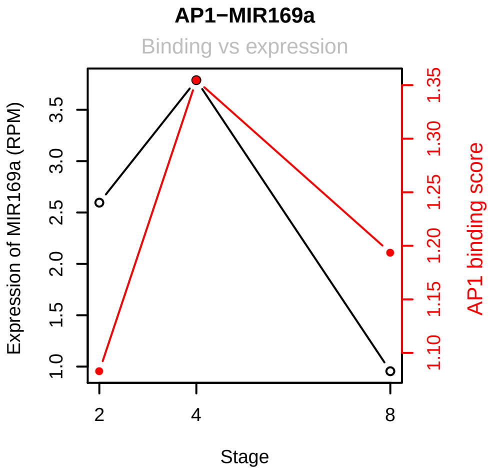

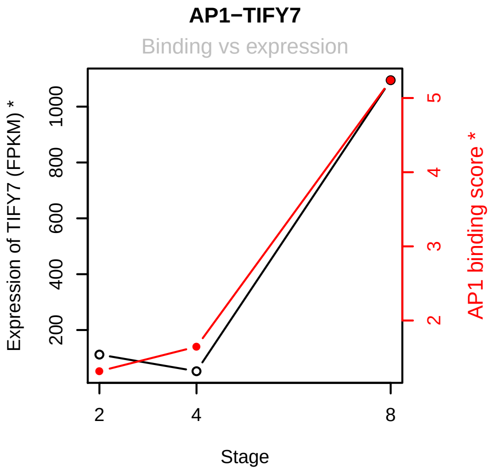

Supplement: Supplementary file 8 — Supplementary Data 6 [file 41467_2018_6772_MOESM8_ESM.pdf]
