# Supplementary material for: Architecture of gene regulatory networks controlling flower development in Arabidopsis thaliana
Source: Nat Commun. 2018 Oct 31;9:4534. doi: 10.1038/s41467-018-06772-3 (PMC6208445; doi:10.1038/s41467-018-06772-3)
Supplement: Supplementary file 11 — Description of Additional Supplementary Files [file 41467_2018_6772_MOESM11_ESM.docx]

**Title:** Supplementary Data 1:
**Description:** ChIP-seq experiments used in this study

**Title:** Supplementary Data 2:
**Description:** Transcription factor binding sites of floral regulators

**Title:** Supplementary Data 3:
**Description:** Target genes by floral regulators

**Title:** Supplementary Data 4:
**Description:** Differentially expressed genes identified by RNA-seq

**Title:** Supplementary Data 5:
**Description:** List of all predicted feed-forward loops (FFLs)

**Title:** Supplementary Data 6:
**Description:** Examples of predicted AP1- or SEP3-regulated FFLs

**Title:** Supplementary Data 7:
**Description:** List of organ-specific genes bound by more than one floral regulators

**Title:** Supplementary Data 8:
**Description:** Primer sequences used in this study
